# Synthesis, Characterization, and In Vitro Cancer Cell Growth Inhibition Evaluation of Novel Phosphatidylcholines with Anisic and Veratric Acids

**DOI:** 10.3390/molecules23082022

**Published:** 2018-08-13

**Authors:** Marta Czarnecka, Marta Świtalska, Joanna Wietrzyk, Gabriela Maciejewska, Anna Gliszczyńska

**Affiliations:** 1 Department of Chemistry, Wrocław University of Environmental and Life Sciences, Norwida 25, 50-375 Wrocław, Poland; 2 Department of Experimental Oncology, Ludwik Hirszfeld Institute of Immunology and Experimental Therapy, Polish Academy of Sciences, Weigla 12, 53-114 Wrocław, Poland; switalska@iitd.pan.wroc.pl (M.Ś.); wietrzyk@iitd.pan.wroc.pl (J.W.); 3 Central Laboratory of the Instrumental Analysis, Wrocław University of Technology, Wybrzeże Wyspiańskiego 27, Wrocław 50-370, Poland; gabriela.maciejewska@pwr.edu.pl

**Keywords:** anisic acid, antiproliferative activity, phenolic acids, phosphatidylcholines, structured phospholipids, veratric acid

## Abstract

Phenolic acids and its methoxy derivatives are known to induce caspase-mediated apoptosis activity and exhibit cytotoxic effect towards various cancer cell lines. However, their low stability and poor bioavailability in the human organism extensively restrict the utility of this group of compounds as anticancer and health-promoting agents. In this report, a series of eight novel phosphatidylcholines (**3a-b**, **5a-b**, **7a-b**, **8a-b**) containing anisic or veratric acids (**1a-b**) at *sn*-1 and/or *sn*-2 positions were synthesized. The phenoylated phospholipids were obtained in good yields 28–66%. The structures of novel compounds were determined by their spectroscopic data. All synthesized compounds were evaluated for their antiproliferative activity towards six cancer cell lines and normal cell line Balb/3T3. Lipophilization of phenolcarboxylic acids significantly increased their anticancer properties. The asymmetrically substituted phenoylated phosphatidylcholines exhibited higher antiproliferative effect than free acids. Lysophosphatidylcholine (**7b**) effectively inhibited the proliferation of human leukaemia (MV4-11), breast (MCF-7), and colon (LoVo) cancer cell lines at concentrations of 9.5–20.7 µm and was from 19 to 38-fold more active than corresponding free veratric acid. The conjugation of anisic/veratric acids with the phosphatidylcholine have proved the anticancer potential of these phenolcarboxylic acids and showed that this type of lipophilization is an effective method for the production of active biomolecules.

## 1. Introduction

Dietary phenolic acids and their methoxy derivatives are one of the most investigated molecules of nutritional interest, since it was discovered that their consumption from natural sources like fruits and vegetables might deliver many health benefits [1,2]. The favorable effects of this group of compounds on human health are mainly due to their antioxidant activities and include the ability to neutralize reactive oxygen species, radical scavenging, and chelating of metal ions [3]. They are reported also as the agents that inhibit cancer cell proliferation in in vitro studies, exhibit anti-inflammatory activity, reduce vascularization, and protect neurons [4,5,6]. Moreover, phenolic acids and their derivatives significantly inhibit intestinal absorption of glucose and increase gut hormone GLP-1, which indicates that they can play also a potential preventative role against type 2 diabetes [7]. Therefore, they are essential to manage these disorders and can be used in the development of new health-promoting food and pharmaceutical ingredients and are effective in the prevention of civilization diseases. 

The main obstacle to the industrial application of phenolic acids and their methoxy derivatives is their low bioavailability in the human organism. It is now well established that these compounds undergo substantial metabolism after being ingested by humans in relevant dietary amount, and that concentration of plasma metabolites after normal dietary intake rarely exceeds nmol/L. A promising strategy to enhance the concentration and beneficial effect of phenolic acids in biological systems is their lipophilization, which modulates the polarity of these bioactive compounds [8]. There are three main lipid carriers that can be used in the process of lipophilization: fatty acids, triacylglycerols, and phospholipids. The use of fatty acids and TAGs has been extensively studied and described in the literature, whereas the contribution of phospholipids to this purpose is innovative [9,10,11,12,13]. Structural modification of phospholipids with phenolic acids is a new topic. Up to now, Yang and co-workers successfully incorporated ferulic acid into the phosphatidylcholine (PC) structure by chemoenzymatic acidolysis catalyzed by lipase Novozym 435, whereas Prasad research group introduced into *sn*-1 position of 1,2-dipalmitoylphosphatidylcholine (DPPC) and egg-yolk PC syringic and vanillic acids, obtaining products with increased antioxidant and antimicrobial activities [14,15]. This convinced us to synthesize the novel phenolic-phospholipids conjugates, especially because in our previous project we proved that it is possible to increase the bioavailability and reduce the active dosage of natural compounds like isoprenoids joined to PC [16,17]. 

We prepared the series of phosphatidylcholines containing the natural methoxyderivatives of benzoic acids: anisic acid (ANISA) and veratric acid (VERA). These acids possess not only antioxidant activity but also a wide range of useful biological properties. Anisic acid (4-methoxybenzoic acid) (ANISA) is the constituent of Chinese star anise (*Illicium verum*) and extract of *Capparis spinose* [18,19]. It was reported that anisic acid protects the liver against toxicity of carbontetrachloride (CCl_4_) and paracetamol (Pcl) [19]. Moreover, it was reported that ANISA exhibits antihepatoxic and antitumor activities [20].

Veratric acid (3,4-dimethoxybenzoic acid) (VERA) is one of the major benzoic derivatives isolated from vegetables and fruits and also occurs naturally in medicinal mushrooms, which have been reported to have anti-inflammatory activities [21]. This compound exhibits also significant inhibitory activity against Gram-positive bacteria such as *Streptococcus pneumonia* with an ID_50_ 64 mg/L [22]. Other documented biological activities of veratric acid indicate that this compound is able to decrease blood pressure by reducing the NO concentration and attenuating oxidative stress in hypertension-induced rats [23]. It is worth noticing that results obtained by Raja showed that oral administration of VERA ameliorates atherogenic diet-induced hyperlipidemia in rats by its free radical scavenging [24].

In our research, we examined the potential anticancer role of dual phenolic-phospholipid biomolecules, and we have tried to determine the correlations between their structure and activity. We synthesized new structured phospholipids containing anisic or veratric acids in *sn*-1 and/or *sn*-2 positions of phosphatidylcholine. This paper makes a substantial contribution to current knowledge from the area of preparation of new biomolecules on the basis of natural compounds that occur in food and evaluates how the structural modifications of active phenolic compounds can increase their bioefficiency and bioavailability in the human organism.

## 2. Results and Discussion

The increasing interest in the phenolic compounds and its derivatives as potential anticancer agents has been triggered by many of studies that have presented their ability to inhibit cancer growth and its progression to advanced stages [25,26]. Although, these studies proved anticancer effect of phenolic acids, they also indicated the biggest drawback, which is their low bioavailability and the need to use higher doses to achieve a therapeutic effect. Therefore, development of method to increase their stability and distribution in the organism is needed. New promising strategy in this context is the synthesis of phenolic compounds covalently bonded to phospholipids (PLs). 

### 2.1. Synthesis of Structured Phospholipids with Anisic and Veratric Acids

**Scheme 1:**
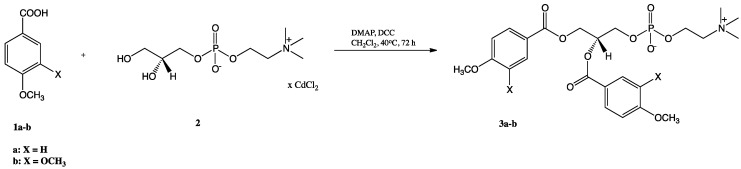
Synthesis of 1,2-diphenoyl-*sn*-glycero-3-phosphocholines (**3a-b**).

Synthesis of structured phosphatidylcholines bearing anisic and veratric acids was achieved via chemoenzymatic approach, and the products were isolated in pure form. We have started the synthetic route from the preparation of 1,2-diphenoyl-*sn*-glycero-3-phosphocholines (**3a-b**) using the cadmium complex of *sn*-glycero-3-phosphocholine (GPC × CdCl_2_) (**2**) and methoxy derivatives of benzoic acid (anisic and veratric acids) as substrates (Scheme 1). Novel products **3a**-**b** were obtained by known Steglich esterification in good yields (49 and 48%, respectively) after 72 h of reaction [27,28,29]. 

**Scheme 2:**
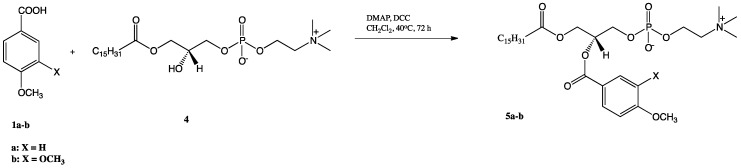
Synthesis of 1-palmitoyl-2-phenoyl-*sn*-glicero-3-phosphocholine (**5a-b**).

The phosphocholines **5a-b** with anisic/veratric acids at the *sn*-2 position were synthesized by reacting respective methoxybenzoic acid with 1-palmitoyl-2-hydroxy-*sn*-glycero-3-phosphocholine (4) in the presence of 4-(*N*,*N*-dimethylamino)pyridine (DMAP) as a catalyst and *N*,*N*′-dicyclohexylcarbodiimide (DCC) as a coupling agent (Scheme 2). The LPC (**4**) was obtained previously by treatment of 1,2-dipalmitoyl-*sn*-glycero-3-phosphocholine with phospholipase A_2_ (PLA_2_) from porcine pancreas [30]. The 1-palmitoyl-2-phenoyl-*sn*-glycero-3-phosphocholines **5a-b** obtained in 28 and 46% yields are new compounds that have not been described in the literature (all data are presented in the Materials and Methods and all spectra are in Supplementary Materials).

**Scheme 3:**
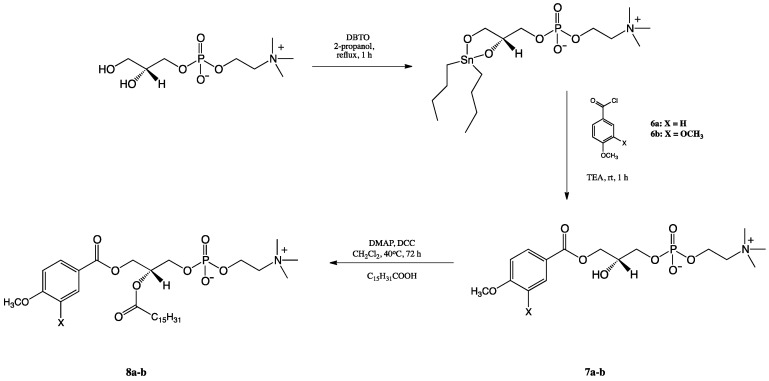
Synthesis of 1-phenoyl-2-palmitoyl-*sn*-glycero-3-phosphocholine (**8a-b**).

Phosphocholines containing anisic/veratric acid at the *sn*-1 position (**8a-b**) were obtained according to the reaction pathway described in Scheme 3. First *sn*-glycerophosphocholine was treated with dibutyltin oxide (DBTO), and the resulting acetal was subjected to the reaction with triethylamine (TEA) and chloride of anisic/veratric acids, which were previously obtained *in-situ* using the procedure described by Mattson [31]. The 1-anisoyl-2-hydroxy-*sn*-glycero-3- phosphocholine (**7a**) was synthesized in 65% yield. 1-Veratroyl-2-hydroxy-*sn*-glycero-3- phosphocholine (**7b**) was obtained in similar 66% yield. The last step of the pathway was esterification reaction of free hydroxy groups in the *sn*-2 position of LPCs (**7a-b**) with palmitic acid (PA) in the presence of DCC and DMAP, leading to products **8a-b** (42 and 52% yields). 

### 2.2. Cytotoxic Activity In Vitro Against Selected Cancer Cell Lines

The antiproliferative activity of conjugates of phosphatidylcholine with anisic and veratric acids (**3a-b**, **5a-b**, **7a-b**, **8a-b**) towards selected cancer cell lines human leukaemia (MV4-11), breast (MCF-7), lung (A549), liver (HepG2), and colon (LoVo) cancer, as well as doxorubicin-resistant colon cancer LoVo/DX (P-gp-dependent, MRP-, LRP-dependent multidrug resistance), were evaluated. In order to determine the degree of toxicity of these new compounds towards healthy cells, the experiments were also carried out against the normal mice fibroblasts (BALB/3T3). Antiproliferative activity of anisic and veratric acids (**1a-b**) was also checked for the comparison. The most widely used chemotherapeutic drugs in clinical trials, cisplatin (cis-diaminedichloroplatinum (II)) and doxorubicin hydrochloride, were applied as positive controls as well. All the results were obtained by cellular viability assessment—sulphorhodamine B (SRB) and 3-(4,5-dimethylthiazol-2-yl)-2,5-diphenyltetrazoliun bromide (MTT) colorimetric assays. The data for the in vitro anticancer activity are reported in Table 1, expressed as the IC_50_ concentration of the compound (in μM) that inhibits proliferation of the cells by 50% compared to the untreated control cells. The IC_50_ values were separately calculated for each experiment, and the mean values ± SD were calculated from at least 3–5 independent experiments. 

As listed in Table 1, almost all synthesized compounds exhibit much higher cytotoxic activity against selected cancer cell lines than anisic or veratric acids used as the control agents. It can be observed that cytotoxic effects of the phenolic-phospholipids strongly depended on their structure and the position occupied by the phenolic residue in the skeleton of PC. An increase in the activity of the obtained compounds was observed in the following order: diphenoyl-PC < monophenoyl-PC < 1-phenoly-LPC. No significant differences in activities of heterosubstituted phosphatidylcholines were observed in this study. It seems that the location of phenolic acids in correlation with palmitic acid does not have bigger influence on the final antiproliferative activity. Interestingly, lysophosphatidylcholines containing anisic or veratric acids in the *sn*-1 position had higher activity than phosphatidylcholones. 1-Anisoyl-2-hydroxy-*sn*-glycero-3-phosphocholine (**7a**) exhibited very strong antiproliferative activity towards leukemia cell line MV4-11 with IC_50_ = 21.1 µm. 1-Veratroyl-2-hydroxy-*sn*-glycero-3-phosphocholine (**7b**) turned out to be the most active compound against leukemia and breast and colon cancer cell lines at concentrations of 9.5 µm, 20.7 µm, and 16.7 µm, respectively. The determined values were from 19 to 38-fold lower than those described for free veratric acid (**1b**). It is also worth noticing that phenolic-phospholipids exhibit the cytotoxic effect towards liver cancer and doxorubicin-resistant colon cancer lines. Studied phenolic acids did not show this activity in the free form but after its incorporation into the PC skeleton.

Phenolic-phospholipids were also able to overcome drug resistance (Table 2), especially 1,2-diveratroyl-*sn*-glycero-3-phosphocholine (**3b**) with RI = 0.42. The most active 1-veratroyl-2-hydroxy-*sn*-glycero-3-phosphocholine (**7b**) had moderate ability to overcome drug resistance (RI = 2.86). Moreover, we did not observe the toxicity of phenolic-phospholipids towards normal mice fibroblasts (BALB/3T3) at the concentration effective towards studied cancer cell lines. Only the most active phenolic-phospholipids **7a** and **7b** exhibited some toxicity against normal fibroblasts.

### 2.3. The Effect of 1-Phenoyl-2-hydroxy-sn-glycero-3-phosphocholines on the Cell Cycle of the MV4-11 Cells

**Figure 1:**
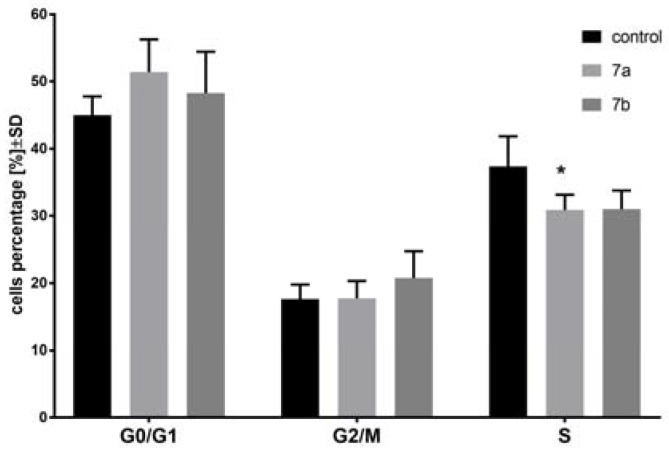
Cell cycle analysis of MV4-11 cells after treatment of 1-anisoyl-2-hydroxy-*sn*-glycero-3- phosphocholine (**7a**; 30 µm) and 1-veratroyl-2-hydroxy-*sn*-glycero-3-phosphocholine (**7b**; 15 µm); * *p* < 0.05 in comparison to control cells, *t*-test, Statistica v.10.

In the next step of the study, two the most active compounds were chosen: 1-anisoyl-2-hydroxy-*sn*-glycero-3-phosphocholine (**7a**) and 1-veratroyl-2-hydroxy-*sn*-glycero-3-phosphocholine (**7b**). Cell cycle of MV4-11 cells was analyzed after 72 h treatment of leukemia cells with **7a** compound in concentration 30 µm and with **7b** compound in concentration 15 µm (Figure 1). These compounds had weak influence on the cell cycle, but it was noticed that **7a** arrested cell cycle in G0/G1 phase (which was statistically insignificant in comparison to the control cells, *p* = 0.062) and lowered percentage of cells in S phase (which was statistically significant in comparison to control cells; *p* < 0.05). After treatment with compound **7b,** a decrease in the number of cells in S phase was reported as well (which was statistically insignificant in comparison to control cells, *p* = 0.053). We observed no or very little influence of these two compounds on the cell cycle; however, the inhibition of cell proliferation was significant. In the cell cycle analysis, we used compounds **7a** and **7b** in concentration similar to IC_50_. The lack of influence on the cell arrest suggests that these compounds were cell-cycle nonspecific agents, which acted during any phases of the cell cycle.

### 2.4. The Effect of 1-Phenoyl-2-hydroxy-sn-glycero-3-phosphocholines on the Mitochondrial Membrane Potential, Cell Death, and Caspase-3/7 Activity of the MV4-11 Cells

**Figure 2:**
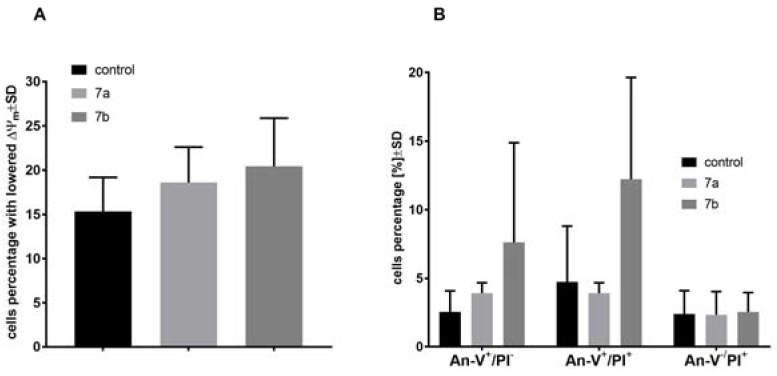
Mitochondrial membrane potential (ΔΨ_m_) (**A**) and cell death analysis (**B**) of MV4-11 cells after treatment of 1-anisoyl-2-hydroxy-*sn*-glycero-3-phosphocholine (**7a**; 30 µm) and 1-veratroyl-2-hydroxy-*sn*- glycero-3-phosphocholine (**7b**; 15 µm).

Mitochondrial membrane potential (ΔΨ_m_), using of JC-1 staining, and cell death, using Annexin-V and PI staining, of MV4-11 cells was analyzed after treatment with **7a** (30 µm) and **7b** (15 µm) compounds. After 72 h of treatment, less influence on mitochondrial membrane potential (Figure 2a) or cell death (Figure 2b) was observed. Compound **7b** had some potential to increase number of cells with lowered ΔΨ_m_ and to increase number of early apoptotic (An-V^+^/PI^−^) and apoptotic cells (An-V^+^/PI^+^) in comparison to control group. No influence of **7a** and **7b** compounds on caspase-3/7 activity was observed (data not shown). 

Compounds **7a** and **7b** were used in concentration of its IC_50_ in both types of analyses: cell cycle distribution and apoptosis. Based on these results, we can observe that compound **7a** was able to arrest cell cycle in G0/G1 phase, leading to a decrease of the subpopulation in the S phase that did not induce apoptosis. The blockage of the cells exposed to **7a** at G0/G1 implies an inhibition of DNA synthesis and resulted in the cell proliferation inhibition. On the other hand, compound **7b** was less potent as cell cycle inhibitor (decrease of the subpopulation in the S phase), but its ability to induce apoptosis was visible. It may suggest that compound **7a** acts rather as a cytostatic, whereas compound **7b** plays a role of a cytotoxic agent. However, further studies are needed to explore exact mechanisms of their action. 

## 3. Materials and Methods 

### 3.1. Materials

Anisic acid (ANISA) (**1a**) and veratric acid (VERA) (**1b**) were purchased from Sigma-Aldrich Chemical Co (St. Louis, MO, USA). *sn*-Glycero-3-phosphocholine (GPC) in the enantiomerically pure form was purchased from Bachem and converted to the cadmium chloride complex (GPC × CdCl_2_) using described earlier method [16]. Phospholipase A_2_ (Lecitase 10L; 10,000 LEU/mL) was a gift from Novozymes (Bagsvaered, Denmark). *N,N’-*Dicyclohexylcarbodiimide (DCC), 4-(*N,N*-dimethylamino)pyridine (DMAP), DBTO, TEA, palmitic acid, oxalyl chloride, dioctyl sulfosuccinate sodium salt (AOT), Dowex^®^ 50WX8 H^+^ form (an ion-exchange resin) were purchased from Sigma–Aldrich, as well as chloroform, methanol, and dichloromethane that were used in reactions and for column and thin-layer chromatography and isooctane for enzymatic hydrolysis. All of the solvents used in liquid chromatography were of HPLC grade (LiChrosolv Reagents) and were purchased from Merck (Kenilworth, NJ, USA).

### 3.2. Methods of Analysis

Analytical thin-layer chromatography (TLC) was performed on Merck Kieselgel 60 F_254_ plates (0.2 mm silica gel with fluorescent indicator UV254, Merck, Kenilworth, NJ, USA) with mixtures of CHCl_3_/CH_3_OH/H_2_O (65:25:4, *v*/*v*/*v*). The compounds were detected by spraying the plates with 0.05% primuline solution acetone/H_2_O (8:2, *v*/*v*), followed by UV (365 nm) visualization, or a solution of 10 g of Ce(SO_4_)_2_ and 20 g of phosphoromolibdenic acid in 1 L of 10% H_2_SO_4_ followed by heating.

Column chromatography was performed on silica gel 60 with 0.1% of Ca (230–400 mesh ASTM, Merck, Kenilworth, NJ, USA) using a solvent mixture of CHCl_3_/CH_3_OH/H_2_O (65:25:4, *v*/*v*/*v*). 

All the NMR spectra were recorded on a Bruker Avance II 600 MHz spectrometer (Brüker, Billerica, MA, USA). 

High resolution mass spectra (HRMS) were obtained using electron spray ionization (ESI) technique on Waters ESI-Q-TOF Premier XE spectrometer (Waters Corporation, Milford, MA, USA).

### 3.3. Methods of Synthesis

#### 3.3.1. Synthesis of 1,2-Diphenoyl-*sn*-glycero-3′-phosphatidylcholines

The GPC × CdCl_2_ complex (**2**) was prepared according to the procedure described before [16]. In the next step, cadmium complex of GPC was acetylated with methoxybenzoic acids in positions *sn*-1 and *sn*-2. To a stirred suspension of GPC × CdCl_2_ complex (100 mg, 0.23 mmol) in 6 mL of anhydrous CH_2_Cl_2_ acids **1a** or **1b** (0.92 mmol), DMAP (56 mg, 0.46 mmol) and, finally, DCC (200 mg, 0.97 mmol) dissolved in 6 mL of the anhydrous CH_2_Cl_2_ were added. The mixture was stirred at 40 °C under a nitrogen atmosphere for 72 h. Next (TLC), the formed precipitate was filtered off using the Schott funnel. The ion-exchange resin (DOWEX 50W X8, H^+^ form) was added to the filtrate in order to dislodge DMAP. The solution was strongly stirred for 30 min; after this time, the resin was filtered off under reduced pressure, and the solvent was evaporated in vacuo. The crude product was purified by column chromatography.

*1,2-Dianisoyl-sn-glycero-3-phosphocholine* (**3a**): Colourless greasy solid (49% yield, *R*_f_ 0.36); ^1^H NMR (600 MHz, CDCl_3_/CD_3_OD 2:1 (*v/v*)), *δ*: 2.92 (s, 9H, -N(CH_3_)_3_), 3.31 (m, 2H, CH_2_-β), 3.60, 3.61 (two s, 6H, 2 × -OCH_3_), 3.96-3.98 (m, 4H, CH_2_-3′, CH_2_-α), 4.30 (dd, *J* = 12.0, 6.6 Hz, 1H, one of CH_2_-1′), 4.42 (dd, *J* = 12.0, 3.5 Hz, 1H, one of CH_2_-1′), 5.32 (m, 1H, H-2′), 6.66, 6.68 (two m, 4H, H-3′′*_sn_*_-1_, H-5′′*_sn_*_-1,_ H-3′′*_sn_*_-2_, H-5′′*_sn_*_-2_), 7.68, 7.73 (two m, 4H, H-2′′*_sn_*_-1_, H-6′′*_sn_*_-1,_ H-2′′*_sn_*_-2_, H-6′′*_sn_*_-2_); ^13^C NMR (150 MHz, CDCl_3_/CD_3_OD 2:1 (*v*/*v*)) *δ*: 53.49, 53.51, 53.53 (-N(CH_3_)_3_), 54.79, 54.81 (2 × -OCH_3_), 58.69 (C-α), 62.55 (C-1′), 63.46 (C-3′), 65.93 (C-β), 70.69 (C-2′), 113.29, 113.31 (C-3′′*_sn_*_-1_, C-5′′*_sn_*_-1_, C-3′′*_sn_*_-2_, C-5′′*_sn_*_-2_), 121.34, 121.38 (C-1′′*_sn_*_-1_, C-1′′*_sn_*_-2_), 131.20, 131.32 (C-2′′*_sn_*_-1_, C-2′′*_sn_*_-2_, C-6′′*_sn_*_-1_, C-6′′*_sn_*_-2_), 163.38, 163.48 (C-4′′*_sn_*_-1_, C-4′′*_sn_*_-2_), 165.60, 165.98 (C-1*_sn_*_-1_, C-1*_sn_*_-2_); ^31^P NMR (121 MHz, CDCl_3_/CD_3_OD 2:1 (*v/v*)) *δ*: −0.70; HRMS (ESI): *m/z* calcd. for C_24_H_32_NO_10_P [M + H]^+^ 526.1842; found 526.1842.

*1,2-Diveratroyl-sn-glycero-3-phosphocholine* (**3b**): Colourless greasy solid (48% yield, *R*_f_ 0.35); ^1^H NMR (600 MHz, CDCl_3_/CD_3_OD 2:1 (*v/v*)), *δ*: 2.97 (s, 9H, -N(CH_3_)_3_), 3.39 (m, 2H, CH_2_-β), 3.60, 3.64, 3.68, 3.69 (four s, 12H, 4 × -OCH_3_), 4.00 (m, 2H, CH_2_-3′), 4.06 (m, 2H, CH_2_-α), 4.33 (dd, *J* = 12, 7.2 Hz, 1H, one of CH_2_-1′), 4.47 (dd, *J* = 12, 2.6 Hz, 1H, one of CH_2_-1′), 5.37 (m, 1H, H-2′), 6.67, 6.69 (two d, *J* = 8.4 Hz, 2H, H-5′′*_sn_*_-1_, H-5′′*_sn_*_-2_), 7.23, 7.28 (four s, 2H, H-2′′*_sn_*_-1_, H-2′′*_sn_*_-2_), 7.42 (two d, *J* = 8.4 Hz, 2H, H-6′′*_sn_*_-1_, H-6′′*_sn_*_-2_); ^13^C NMR (150 MHz, CDCl_3_/CD_3_OD 2:1 (*v/v*)) δ: 53.53 (-N(CH_3_)_3_), 55.29, 55.41, 55.45 (4 × -OCH_3_), 58.96 (C-α), 62.70 (C-1′), 63.65 (C-3′), 65.83 (C-β), 70.74 (C-2′), 110.08, 110.10 (C-5′′*_sn_*_-1_, C-5′′*_sn_*_-2_), 111.64, 111.76 (C-2′′*_sn_*_-1_, C-2′′*_sn_*_-2_), 121.40, 121.43 (C-1′′*_sn_*_-1_, C-1′′*_sn_*_-2_), 123.48, 123.64 (C-6′′*_sn_*_-1_, C-6′′*_sn_*_-2_), 148.19, 148.28, 152.99, 153.16 (C-3′′*_sn_*_-1_, C-3′′*_sn_*_-2_, C-4′′*_sn_*_-1_, C-4′′*_sn_*_-2_), 165.64, 165.94 (C-1*_sn_*_-1_, C-1*_sn_*_-2_); ^31^P NMR (121 MHz, CDCl_3_/CD_3_OD 2:1 (*v/v*)) *δ*: −3.40; HRMS (ESI): *m/z* calcd. for C_26_H_36_NO_12_P [M + H]^+^ 586.2053; found 586.2058.

#### 3.3.2. Synthesis of 1-palmitoyl-2-phenoyl-*sn*-glycero-3′-phosphatidylcholines

Methoxybenzoic acid **1a** or **1b** (0.604 mmol), solution of DMAP ((74 mg, 0.6 mmol) in 3 mL of CH_2_Cl_2_ and DCC (267 mg, 1.3 mmol) in 3 mL of CH_2_Cl_2_, were added to the solution of previously obtained 1-palmitoyl-2-hydroxy-*sn*-3-glycero-phosphocholine (**4**) (150 mg, 0.302 mmol in 4 mL of anhydrous CH_2_Cl_2_). The reaction was carried out for 72 h in a nitrogen atmosphere in the dark at 40 °C. Next, the product was extracted according to the procedure described for compound **3a** and **3b,** and was purified by column chromatography.

*1-Palmitoyl-2-anisoyl-sn-glycero-3-phosphocholine* (**5a**): Colourless greasy solid (28% yield, *R*_f_ 0.17); ^1^H NMR (600 MHz, CDCl_3_/CD_3_OD 2:1 (*v/v*)), δ: 0.64 (t, *J* = 7.2 Hz, 3H, CH_3_(CH_2_)_13_CH_2_C(O)), 1.02-1.13 (m, 24H, CH_3_(CH_2_)_12_CH_2_CH_2_C(O)), 1.30 (m, 2H, CH_3_(CH_2_)_12_CH_2_CH_2_C(O)), 2.05 (t, *J* = 7.5 Hz, 2H, CH_3_(CH_2_)_13_CH_2_C(O)), 2.97 (s, 9H, -N(CH_3_)_3_), 3.39 (m, 2H, CH_2_-β), 3.63 (s, 3H, -OCH_3_), 3.90 (m, 2H, CH_2_-3′), 4.04–4.32 (3 m, 4H, CH_2_-α, CH_2_-1′), 5.20 (m, 1H, H-2′), 6.71 (m, 2H, H-3′′, H-5′′), 7.73 (m, 2H, H-2′′, H-6′′); ^13^C NMR (150 MHz, CDCl_3_/CD_3_OD 2:1 (*v/v*)) *δ*: 13.50 (CH_3_(CH_2_)_13_CH_2_C(O)), 22.24 (CH_3_CH_2_(CH_2_)_12_CH_2_C(O)), 24.46 (CH_3_(CH_2_)_12_CH_2_CH_2_C(O)), 28.67, 28.85, 28.88, 28.93, 29.02, 29.05, 29.07, 29.21, 29.22, 29.26, 31.50 (CH_3_CH_2_(CH_2_)_11_CH_2_CH_2_C(O)), 33.68 (CH_3_(CH_2_)_13_CH_2_C(O)), 53.67 ((-N(CH_3_)_3_), 55.01 (-OCH_3_), 59.00 (C-α), 62.26 (C-1′), 63.68 (C-3′), 65.90 (C-β), 70.63 (C-2′), 113.45 (C-3′′, C-5′′), 121.29 (C-1′′), 131.42 (C-2′′, C-6′′), 163.60 (C-4′′), 165.63, 173.71 (C-1, (CH_3_(CH_2_)_13_CH_2_C(O)); ^31^P NMR (121 MHz, CDCl_3_/CD_3_OD 2:1 (*v/v*)) *δ*: −2.76; HRMS (ESI): *m/z* calcd. for C_32_H_56_NO_9_P [M + H]^+^ 630.3771; found 630.3776.

*1-Palmitoyl-2-veratroyl-sn-glycero-3-phosphocholine* (**5b**): Colourless greasy solid (46% yield, *R*_f_ 0.19); ^1^H NMR (600 MHz, CDCl_3_/CD_3_OD 2:1 (*v/v*)), *δ*: 0.65 (t, *J* = 6.7 Hz, 3H, CH_3_(CH_2_)_13_CH_2_C(O)), 0.98–1.07 (m, 24H, CH_3_(CH_2_)_12_CH_2_CH_2_C(O)), 1.33 (m, 2H, CH_3_(CH_2_)_12_CH_2_CH_2_C(O)), 2.08 (t, *J* = 7.5 Hz, 2H, CH_3_(CH_2_)_13_CH_2_C(O)), 2.98 (s, 9H, -N(CH_3_)_3_), 3.39 (m, 2H, CH_2_-β), 3.70, 3.72 (two s, 6H, 2 × -OCH_3_), 3.91 (m, 2H, CH_2_-3′), 4.05 (m, 2H, CH_2_-α), 4.17 (dd, *J* = 12.0, 7.2 Hz, 1H, one of CH_2_-1′), 4.22 (dd, *J* = 12.0, 3.0 Hz, 1H, one of CH_2_-1′), 5.22 (m, 1H, H-2′), 6.73 (d, *J* = 8.4 Hz, 1H, H-5′′), 7.30 (s, 1H, H-2′′), 7.45 (d, *J* = 8.4 Hz, 1H, H-6′′); ^13^C NMR (150 MHz, CDCl_3_/CD_3_OD 2:1 (*v/v*)) δ: 13.39 (CH_3_(CH_2_)_13_CH_2_C(O)), 22.17 (CH_3_CH_2_(CH_2_)_12_CH_2_C(O)), 24.41 (CH_3_(CH_2_)_12_CH_2_CH_2_C(O)), 28.61, 28.79, 28.86, 28.95, 29.13, 29.15, 29.18, 31.43 (CH_3_CH_2_(**C**H_2_)_11_CH_2_CH_2_C(O)), 33.62 (CH_3_(CH_2_)_13_CH_2_C(O)), 53.53 ((-N(CH_3_)_3_), 55.48 (2 × -OCH_3_), 58.92 (C-α), 62.11 (C-1′), 63.59 (C-3′), 65.84 (C-β), 70.76 (C-2′), 110.08 (C-5′′), 111.79 (C-2′′), 121.39 (C-1′′), 123.65 (C-6′′), 148.33, 153.19 (C-3′′, C-4′′), 165.63, 173.66 (C-1, (CH_3_(CH_2_)_13_CH_2_C(O)); ^31^P NMR (121 MHz, CDCl_3_/CD_3_OD 2:1 (*v/v*)) *δ*: −3.66; HRMS (ESI): *m/z* calcd. for C_33_H_58_NO_10_P [M + H]^+^ 660.3876; found 660.3882.

#### 3.3.3. Synthesis of 1-Phenoyl-2-hydroxy-*sn*-glycero-3′-phosphatidylcholines

*sn*-Glycero-3-phosphocholine (GPC) (257 mg, 1 mmol) and dibutyltin oxide (DBTO) (249 mg, 1 mmol) were suspended in 12 mL of anhydrous propan-2-ol and refluxed for 1 h. The solution was cooled to the room temperature and TEA (242 mg, 2.4 mmol) was added followed by chloride of methoxybenzoic acid (2.4 mmol). The solution was stirred for 1 h, and then mixture was filtrated using diatomaceous earth (Celite^®^ 545), and the solvent was evaporated. The crude product was purified by silica gel chromatography. 

*1-Anisoyl-2-hydroxy-sn-glycero-3-phosphocholine* (**7a**): Colourless greasy solid (65% yield, *R*_f_ 0.15); ^1^H NMR (600 MHz, CDCl_3_/CD_3_OD 2:1 (*v/v*)), *δ*: 3.02 (s, 9H, -N(CH_3_)_3_), 3.49 (m, 2H, CH_2_-β), 3.63 (s, 3H, -OCH_3_), 3.77–3.78 (2 m, 3H, CH_2_-3′, H-2′), 3.91 (m, 1H, -OH), 4.07–4.14 (two m, 4H, CH_2_-1′, CH_2_-α,), 6.70–6.72 (m, 2H, H-3′′, H-5′′), 7.75–7.77 (m, 2H, H-2′′, H-6′′); ^13^C NMR (151 MHz, CDCl_3_/CD_3_OD 2:1 (v/v)) *δ*: 53.48–53.56 ((-N(CH_3_)_3_), 54.78 (-OCH_3_), 59.04 (C-α), 64.77 (C-1′), 65.71 (C-β), 66.56 (C-3′), 67.38 (C-2′), 113.25 (C-3′′, C-5′′), 121.39 (C-1′′), 131.19 (C-2′′, C-6′′), 163.37 (C-4′′), 166.27 (C-1); ^31^P NMR (243 MHz, CDCl_3_/CD_3_OD 2:1 (*v/v*)) *δ*: −4.00; HRMS (ESI): *m/z* calcd. for C_16_H_26_NO_8_P [M + H]^+^ 392.1474; found 392.1477.

*1-Veratroyl-2-hydroxy-sn-glycero-3-phosphocholine* (**7b**): Colourless greasy solid (66% yield, *R*_f_ 0.14); ^1^H NMR (600 MHz, CDCl_3_/CD_3_OD 2:1 (*v/v*)), *δ*: 3.02 (s, 9H, -N(CH_3_)_3_), 3.49 (m, 2H, CH_2_-β), 3.68, 3.70 (2 s, 6H, 2 × -OCH_3_), 3.76–3.81 (2 m, 3H, CH_2_-2′, CH_2_-3′), 3.92 (m, 1H, -OH), 4.07–4.22 (2 m, 4H, CH_2_-α, CH_2_-1′), 6.70 (d, *J* = 8.5 Hz, 1H, H-5′′), 7.31 (s, 1H, H-2′′), 7.47 (2 d, *J* = 8.5 Hz, 1H, H-6′′); ^13^C NMR (150 MHz, CDCl_3_/CD_3_OD 2:1 (*v/v*)) *δ*: 53.48 ((-N(CH_3_)_3_), 55.34 (-OCH_3_), 59.08 (C-α), 65.00 (C-1′), 65.72 (C-β), 66.58 (C-3′), 68.07 (C-2′), 110.02 (C-5′′), 111.70 (C-2′′), 121.62 (C-1′′), 123.46 (C-6′′), 148.21 (C-3′′), 152.96 (C-4′′), 166.26 (C-1); ^31^P NMR (121 MHz, CDCl_3_/CD_3_OD 2:1 (*v/v*)) *δ*: −4.00; HRMS (ESI): *m/z* calcd. for C_17_H_28_NO_9_P [M + H]^+^ 422.1580; found 422.1576.

#### 3.3.4. Synthesis of 1-Phenoyl-2-palmitoyl-sn-glycero-3-phosphatidylcholines

Lysophosphatidylcholines (**7a**-**7b**) (0.14 mmol) were dissolved in 1 mL of anhydrous CH_2_Cl_2_. Palmitic acid (0.56 mmol) in 2 mL of CH_2_Cl_2_, DMAP (0.28 mmol in 2 mL of CH_2_Cl_2_) and, finally, a solution of DCC (0.58 mmol in 3 mL) were added to the mixture. The reaction was carried out for 72 h in the dark at 40 °C. Then, the product was extracted according to the method described for compounds **3a** and **3b**. The crude product was purified by silica gel chromatography. 

*1-Anisoyl-2-palmitoyl-sn-glycero-3-phosphocholine* (**8a**): Colourless greasy solid (42% yield, *R*_f_ 0.27); ^1^H NMR (600 MHz, CDCl_3_/CD_3_OD 2:1 (*v/v*)), *δ*: 0.65 (t, *J* = 7.1 Hz, 3H, CH_3_(CH_2_)_13_CH_2_C(O)), 0.98–1.03 (m, 24H, CH_3_(CH_2_)_12_CH_2_CH_2_C(O)), 1.35 (m, 2H, CH_3_(CH_2_)_12_CH_2_CH_2_C(O)), 2.10 (t, *J* = 7.8 Hz, 2H, CH_3_(CH_2_)_13_CH_2_C(O)), 3.02 (s, 9H, -N(CH_3_)_3_), 3.45 (m, 2H, CH_2_-β), 3.64 (s, 3H, -OCH_3_), 3.86 (m, 2H, CH_2_-3′), 4.09 (m, 2H, CH_2_-α), 4.18 (dd, *J* = 12.0, 7.1 Hz, 1H, one of CH_2_-1′), 4.32 (dd, *J* = 12.0, 3.0 Hz, 1H, one of CH_2_-1′), 5.15 (m, 1H, H-2′), 6.71 (m, 2H, H-3′′, H-5′′), 7.72 (m, 2H, H-2′′, H-6′′); ^13^C NMR (151 MHz, CDCl_3_/CD_3_OD 2:1 (*v/v*)) *δ*: 13.26 (CH_3_(CH_2_)_13_CH_2_C(O)), 22.08 (CH_3_CH_2_(CH_2_)_12_CH_2_C(O)), 24.40 (CH_3_(CH_2_)_12_CH_2_CH_2_C(O)), 28.51, 28.75, 28.77, 28.88, 29.07, 29.10, 31.35 (CH_3_CH_2_(CH_2_)_11_CH_2_CH_2_C(O)), 33.68 (CH_3_(CH_2_)_13_CH_2_C(O)), 53.42 ((-N(CH_3_)_3_), 54.77 (-OCH_3_), 59.02 (C-α), 62.45 (C-1′), 63.55 (C-3′), 65.71 (C-β), 69.84 (C-2′), 113.23, 113.29 (C-3′′, C-5′′), 121.14 (C-1′′), 131.18 (C-2′′, C-6′′), 163.44 (C-4′′), 165.87, 173.22 (C-1, (CH_3_(CH_2_)_13_CH_2_C(O)); ^31^P NMR (243 MHz, CDCl_3_/CD_3_OD 2:1 (*v/v*)) *δ*: −3.43; HRMS (ESI): *m/z* calcd. for C_32_H_56_NO_9_P [M + H]^+^ 630.3771; found 630.3778.

*1-Veratroyl-2-palmitoyl-sn-glycero-3-phosphocholine* (**8b**): Colourless greasy solid (52% yield, *R*_f_ 0.29); ^1^H NMR (600 MHz, CDCl_3_/CD_3_OD 2:1 (*v/v*)), *δ*: 0.64 (t, *J* = 7.0 Hz, 3H, CH_3_(CH_2_)_13_CH_2_C(O)), 0.97–1.02 (m, 24H, CH_3_(CH_2_)_12_CH_2_CH_2_C(O)), 1.33 (m, 2H, CH_3_(CH_2_)_12_CH_2_CH_2_C(O)), 2.10 (t, *J* = 7.5 Hz, 2H, CH_3_(CH_2_)_13_CH_2_C(O)), 3.02 (s, 9H, -N(CH_3_)_3_), 3.46 (m, 2H, CH_2_-β), 3.69, 3.71 (two s, 6H, 2 × -OCH_3_), 3.86 (m, 2H, CH_2_-3′), 4.09 (m, 3H, CH_2_-α, one of CH_2_-1′), 4.33 (dd, *J* = 12.0, 7.2 Hz, 1H, one of CH_2_-1′), 5.19 (m, 1H, H-2′), 6.70 (d, *J* = 8.5 Hz, 1H, H-5′′), 7.27 (s, 1H, H-2′′), 7.42 (d, *J* = 8.5 Hz, 1H, H-6′′); ^13^C NMR (151 MHz, CDCl_3_/CD_3_OD 2:1 (*v/v*)) *δ*: 13.27 (CH_3_(CH_2_)_13_CH_2_C(O)), 22.08 (CH_3_CH_2_(CH_2_)_12_CH_2_C(O)), 24.43 (CH_3_(CH_2_)_12_CH_2_CH_2_C(O)), 28.54, 28.76, 28.77, 28.89, 29.07, 29.11, 31.35 (CH_3_CH_2_(CH_2_)_11_CH_2_CH_2_C(O)), 33.71 (CH_3_(CH_2_)_13_CH_2_C(O)), 53.48 ((-N(CH_3_)_3_), 55.34 (2 × -OCH_3_), 58.97 (C-α), 62.80 (C-1′), 63.54 (C-3′), 65.75 (C-β), 69.84 (C-2′), 110.00 (C-5′′), 111.62 (C-2′′), 121.35 (C-1′′), 123.43 (C-6′′), 148.22, 153.00 (C-3′′, C-4′′), 165.84, 173.18 (C-1, (CH_3_(CH_2_)_13_CH_2_C(O)); ^31^P NMR (243 MHz, CDCl_3_/CD_3_OD 2:1 (*v/v*)) *δ*: −2.95; HRMS (ESI): *m/z* calcd. for C_33_H_58_NO_10_P [M + H]^+^ 660.3876; found 660.3881.

### 3.4. Biological Studies

#### 3.4.1. Cell Line

Established in vitro human cell lines MV4-11 (human biphenotypic B myelomonocytic leukemia), A549 (non-small cell lung cancer), MCF-7 (breast cancer), LoVo (colon cancer), LoVo/DX (colon cancer drug resistant), HepG2 (liver cancer), and BALB/3T3 (normal mice fibroblasts) were used. All lines were obtained from American Type Culture Collection (Rockville, Maryland, USA) and are being maintained at the Institute of Immunology and Experimental Therapy, Wroclaw, Poland. MV4-11 cells were cultured in the RPMI 1640 medium (IIET, Wrocław, Poland) supplemented with 1.0 mM sodium pyruvate and 10% fetal bovine serum (all from Sigma Aldrich, Chemie Gmbh Munich, Germany). A549, LoVo, and LoVo/DX cells were cultured in the mixture of RPMI 1640 + OptiMEM (1:1) medium (IIET, Poland) supplemented with 5% fetal bovine serum, 1.0 mM sodium pyruvate (LoVo and LoVo/DX cells) (all from Sigma Aldrich, Chemie Gmbh Munich, Germany), and 0.1 µg/mL doxorubicin chloride (Accord) (only LoVo/DX cells). MCF-7 cells were cultured in the Eagle’s medium (IIET, Poland) supplemented with 10% fetal bovine serum (Sigma Aldrich, Chemie Gmbh Munich, Germany). HepG2 and BALB/3T3 cells were cultured in DMEM medium (Gibco) supplemented with 10% fetal bovine serum and 1.0 mM sodium pyruvate (BALB/3T3) (all from Sigma Aldrich, Chemie Gmbh Munich, Germany). All culture mediums were supplemented with 2 mM L-glutamine, 100 units/mL penicillin (Sigma Aldrich, Chemie Gmbh Munich, Germany), and 100 µg/mL streptomycin (Polfa Tarchomin S.A., Warszawa, Poland). All cell lines were grown at 37 °C with 5% CO_2_ humidified atmosphere.

#### 3.4.2. Cytotoxicity Assay In Vitro

Test solutions of the compounds tested (50 mM) were prepared by dissolving the substances in DMSO (Sigma Aldrich, Chemie Gmbh Munich, Germany). Afterwards, the tested compounds were diluted in culture medium to reach the final concentrations of 625, 125, 25, and 5 μM.

24 h prior to the addition of the tested compounds, the cells were plated in 96-well plates (Sarstedt, Germany) at a density of 1 × 10^4^ cells per well. The assay was performed after 72 h of exposure to varying concentrations of the tested agents. The in vitro cytotoxic effect of all agents was examined using the MTT (MV4-11) or SRB assay [16]. The results were calculated as IC_50_ (inhibitory concentration 50%) of the concentration of tested agent, which is cytotoxic for 50% of cancer cells. IC values were calculated for each experiment separately, and mean values ± SD are presented in Table 1. Each compound in each concentration was tested in triplicate in a single experiment, which was repeated 3–5 times.

#### 3.4.3. MTT Assay

This technique was applied for the cytotoxicity screening against leukemia cells growing in suspension culture. An assay was performed after 72 h exposure to varying concentrations of the tested agents. For the last 4 h of incubation, 20 µL of MTT solution were added to each well (MTT: 3-(4,5-dimethylthiazol-2-yl)-2,5-diphenyl tetrazolium bromide; stock solution: 5 mg/mL, Sigma Aldrich, Germany). The mitochondria of viable cells reduce the pale yellow MTT to a navy-blue formazan: the more viable cells are present in well, the more MTT will be reduced to formazan. When incubation time was completed, 80 µL of the lysing mixture was added to each well (lysing mixture: 225 mL dimethylformamide, POCh, Gliwice, Poland, 67.5 g sodium dodecyl sulfate, Sigma Aldrich, Germany, and 275 mL of distilled water). After 24 h, when formazan crystals had been dissolved, the optical densities of the samples were read on Synergy H4 photometer (BioTek Instruments, Winooski, VT, USA) at 570 nm wavelength. The background optical density was measured in the wells filled with culture medium, without the cells.

#### 3.4.4. SRB Assay

This technique was applied for the cytotoxicity screening against cells growing in adherent culture. The details of this technique were described by Skehan [33]. The cytotoxicity assay was performed after 72 h exposure of the cultured cells to varying concentrations of the tested agents. The cells attached to the plastic were fixed by gently layering cold 50% TCA (trichloroacetic acid, Sigma Aldrich, Chemie Gmbh Munich, Germany) on the top of the culture medium in each well. The plates were incubated at 4 °C for 1 h and then washed five times with tap water. The cellular material fixed with TCA was stained with 0.1% sulforhodamine B (SRB, Sigma Aldrich, Chemie Gmbh Munich, Germany) dissolved in 1% acetic acid (POCh, Gliwice, Poland) for 30 min. Unbound dye was removed by rinsing (4×) with 1% acetic acid. The protein-bound dye was extracted with 10 mM unbuffered Tris base (Sigma Aldrich, Chemie Gmbh Munich, Germany) for determination of optical density (at 540 nm) on Synergy H4 photometer (BioTek Instruments, Winooski, VT, USA). The background optical density was measured in the wells filled with culture medium, without the cells.

#### 3.4.5. Cell Cycle Analysis 

The MV4-11 cells were seeded at a density of 1 × 10^5^ cells/ml of culture medium on 24-well plates (Sarstedt, Nümbrecht, Germany) to the final volume of 2 mL. The cells were exposed to the test compounds **7a** and **7b** at concentrations of 30 µm (**7a**) or 15 µm (**7b**) for 72 h. After incubation, the cells were collected, and 1 × 10^6^ of cells were washed twice in cold PBS and fixed for 24 h in 70% ethanol at −20 °C. Then, the cells were washed twice in PBS and incubated with RNAse (8 μg/mL, Fermentas, St. Leon-Rot, Germany) at 37 °C for 1 h. The cells were stained for 30 min. with propidium iodide (50 μg/mL, Sigma Aldrich, Chemie Gmbh Munich, Germany) at 4 °C, and the cellular DNA content was analyzed by flow cytometry using BD LSRFortessa cytometer (BD Bioscience, San Jose, CA, USA). Compounds at each concentration were tested at least three times independently. Obtained results were analyzed using ModFit 3.2 software (Verity Software, Topsham, ME, USA).

#### 3.4.6. Apoptosis Determination by Annexin V Staining

The MV4-11 cells were seeded at the density of 1 × 10^5^ cells/ml of culture medium on 24-well plates (Sarstedt, Nümbrecht, Germany) to the final volume of 2 mL. The cells were exposed to the test compounds **7a** and **7b** at concentrations 30 µm (**7a**) or 15 µm (**7b**) for 72 h. After incubation, the cells were collected, and 2 × 10^5^ of cells were washed twice with PBS. APC-Annexin V (BD Pharmingen, San Jose, CA, USA) was dissolved to the concentration of 1 mg/mL in a binding buffer (Hepes buffer: 10 mM HEPES/NaOH, pH 7.4, 150 mM NaCl, 5 mM KCl, 1 mM MgCl_2_, 1.8 mM CaCl_2,_ (IIET, Wrocław, Poland), and the cells were suspended in 200 µL of this 1 mg/mL solution (freshly prepared each time). After 15 min of the incubation in the dark at room temperature, the propidium iodide (PI) solution (1 mg/mL) was added prior to the analysis to give a final concentration of 0.1 mg/mL. Data acquisition was performed by flow cytometry using BD LSRFortessa cytometer (BD Bioscience, San Jose, CA, USA). Compounds at each concentration were tested at least three times independently. Results were analyzed using Flowing software 2.5.1 (Turku, Finland). The data were displayed as a two-color dot plot with an APC-Annexin V vs. PI. Double-negative cells were live cells, PI+/Annexin V+ were late apoptotic or necrotic cells, and PI-/Annexin V+ were early apoptotic cells. 

#### 3.4.7. Mitochondrial Membrane Potential Determination

The mitochondrial injury was assessed by JC-1 (Sigma Aldrich, Chemie Gmbh Munich, Germany) staining. This dye, existing in the cytosol as a monomer, remained unprocessed due to the breakdown of the mitochondrial membrane potential and fluoresced green. JC-1 can assume a dimeric configuration in the mitochondria and fluorescent red in the reaction driven by the mitochondrial transmembrane potential. The MV4-11 cells were seeded at a density of 1 × 10^5^ cells/ml of culture medium on 24-well plates (Sarstedt, Nümbrecht, Germany) to the final volume of 2 mL. The cells were exposed to the test compounds **7a** and **7b** at concentrations 30 µm (**7a**) or 15 µm (**7b**) for 72 h. After incubation, the cells were collected, and 5 × 10^5^ cells were washed in PBS containing 2% FBS. The pelleted cells were resuspended in 100 µL of the warm cultured medium with the addition of 10 µL of JC-1 (the final concentration of JC-1 was 3 µg/mL) and incubated for 30 min at 37 °C. Next, the cells were washed with 1 mL of PBS + 2% FBS and resuspended in 300 µL of PBS + 2% FBS. The mitochondrial membrane potential was analysed by flow cytometry using BD LSRFortessa cytometer (BD Bioscience, San Jose, CA, USA). Compounds at each concentration were tested at least three times independently. Results were analyzed using Flowing software 2.5.1.

#### 3.4.8. Caspase-3/7 Activity Determination

The MV4-11 cells were seeded at the density of 1 × 10^5^ cells/ml of culture medium on 24-well plates (Sarstedt, Germany) to the final volume of 2 ml. The cells were exposed to the test compounds **7a** and **7b** at concentrations 30 µm (**7a**) or 15 µm (**7b**) for 72 h. After 72 h of the incubation, the cells were collected and centrifuged (5 min, 4 °C, 250× *g*). Cells were suspended in 50 µL of ice-cold lysis buffer (50 mM HEPES, 10% (*w/v*) sucrose, 150 mM NaCl, 2 mM EDTA, 1% (*v/v*) Triton X-100, and pH 7.3, IIET, Poland) and incubated for 30 min. at 4 °C. After the incubation, 40 µL of each sample was transferred to a white, 96-well plate (Corning, NY, USA) containing 160 µL of the reaction buffer (20 mM HEPES, 10% sucrose, 100 mM NaCl, 1 mM EDTA, 10 mM DTT, and 0.02% Trition X-100, pH 7.3) (IIET, Wroclaw, Poland) with 9 µm Ac-DEVD-ACC fluorogenic substrate (λ_ex_ = 360 nm, λ_em_ = 460 nm). The fluorescence increase correlated with the caspase-3/7 level was continuously recorded at 37 °C for 90 min using a Biotek Synergy H4 (Biokom, Warsaw, Poland). Compounds at each concentration were tested in triplicates in single experiment, and each experiment was repeated at least three times independently. Results were normalized to the number of cells in each well and are reported as mean relative caspase-3/7 activity compared to untreated control sample ± SD.

#### 3.4.9. Statistical Analysis

Statistical analysis was performed in Statsoft Statistica 10. All datasets were analyzed using *t*-test. *p*-Values lower than 0.05 were considered as statistically significant. 

## 4. Conclusions

In the series of tested phenolic-phospholipids it can be reported that its cytotoxic or cytostatic effects depend on their chemical structure. The highest antiproliferative activity was observed for 1-phenoly-LPC, whereas the lowest was observed for diphenoyl-PC. The location of phenolic acids in correlation with palmitic acid does not have a significant influence on antiproliferative activity. Two compounds, **7a** and **7b,** turned out to have the highest antiproliferative activity against tested cancer cell lines. The active concentrations of these compounds, which inhibit the proliferation of leukaemia cells, were 12- to 38-fold lower than those determined for free corresponding phenolcarboxylic acids. Compound **7a** arrested the cell cycle in G0/G1 phase and decreased the subpopulation of cells in the S phase, followed by proliferation inhibition via cytostatic action, while derivative **7b** induced apoptosis (cytotoxic action). To sum up the results, it can be concluded that the chemical structure of the phenolic unit in the synthesized conjugates determines their mechanism of antiproliferative activity; however, further studies are necessary to find out molecular targets of tested compounds.

## Figures and Tables

**Table 1:** Antiproliferative activity of phenolic-phospholipids against human cancer cell lines.

Compounds	Acyl Residue		Cell Lines IC_50_ [μm]						
	*sn*-1	*sn*-2	MV4-11	A-549	MCF-7	LoVo	LoVo/DX	HepG2	BALB/3T3
Palmitic acid	-	-	161 ± 78.9	56.4 ± 1.6	122.6 ± 63.4	42.1 ± 4.6	86.3 ± 21.3	275.1 ± 85.2	60.4 ± 4
DPPC	PA	PA	178.7 ± 30.3	229.7 ± 31.3	398.2 ± 1 93.9	274.2 ± 17.1	n.a.	n.a.	48.8 ± 2.1
**1-PA-LPC**	PA	-	64 ± 0.9	62.3 ± 6.5	73 ± 4.6	52.4 ± 4.3	58 ± 3.7	282.7 ± 12.5	176.2 ± 11.3
**1a (ANISA)**	-	-	264.6 ± 49.2	n.a.	483 ± 60.1	312.5 ± 29.6	n.a.	n.a.	n.a.
**3a**	ANISA	ANISA	404.3 ± 163.7 ′′	n.a.	n.a.	319.2 ± 24.2 ′′	n.a.	n.a.	n.a.
**5a**	PA	ANISA	59.6 ± 6.3 *^′′	56.1 ± 1.3 ′′	73.1 ± 8.4 *′′	52 ± 2.6 *^′′	57.7 ± 0.1 ′′	289.7 ± 8.2 ′′	121 ± 25.9 ′′
**7a**	ANISA	-	21.1 ± 4.9 *^^#&^	198.4 ± 14.1 ^#&^	48.4 ± 2.4 *^#&^	43.5 ± 3.2 *^^#&^	70.1 ± 2.9 ^#&^	82.8 ± 9.6 ^#&^	52.9 ± 8.4 ^#&^
**8a**	ANISA	PA	79.5 ± 6.4 *^^#^′′	59.2 ± 0.2 ′′	79.4 ± 8.1 *′′	59.3 ± 4.6 *^′′	59.1 ± 0.9 ′′	275.9 ± 12′′	201.7 ± 22.4 ^#^′′
**1b (VA)**	-	-	366.3 ± 70.8	n.a.	578.6 ± 28.9	316.4 ± 30	n.a.	n.a.	n.a.
**3b**	VA	VA	463.7 ± 38.4 ′′	n.a.	610.5 ± 5.7 ′′	285.5 ± 43.6 ′′	120.4 ± 49.9	51.4 ± 5.8 ′′	354.6 ± 60.2 ′′
**5b**	PA	VA	156.7 ± 66.1 *^′′	68.8 ± 1	118.6 ± 24.5 *^′′	58.8 ± 9.5 *^′′	59.9 ± 0.9 ′′	274.4 ± 21.7 ^′′	251.5 ± 4.3 ^′′
**7b**	VA	-	9.5 ± 2.1 *^^#&^	65.4 ± 5	20.7 ± 4.3 *^^#&^	16.7 ± 1.9 *^^#&^	47.8 ± 3.9 ^#&^	53.1 ± 7.2 ^#&^	33.2 ± 3.4 ^^#&^
**8b**	VA	PA	164.1 ± 67.1 *^′′	82.4 ± 16	86.2 ± 8.8 *^′′	63.1 ± 6.5 *^′′	65.6 ± 0.7 ^#^′′	289.6 ± 7.5 ^′′	253.1 ± 11.8 ^′′
Cisplatin			1.3 ± 0.47	8.6 ± 0.7	8.1 ± 0.03	2.56 ± 0.35	3.17 ± 0.2	2.38 ± 0.64	4.2 ± 1.1
Doxorubicin				-	-	0.117 ± 0.012	6.53 ± 0.93	-	-

n.a.—no activity in concentration of 5, 25, 125, 625 μM. IC_50_—compound concentration leading to 50% inhibition of cell proliferation. Data are presented as mean ± standard deviation (SD) calculated using Prolab-3 system based on Cheburator 0.4 software [32]. *—results within column that are significantly different in comparison to ANISA or VA, respectively; *p* < 0.05. ^—results within column that are significantly different in comparison to **3a** or **3b**, respectively; *p* < 0.05. ^#^—results within column which are significantly different in comparison to **5a** or **5b**, respectively; *p* < 0.05. ′′—results within column which are significantly different in comparison to **7a** or **7b**, respectively; *p* < 0.05. ^&^ — results within column which are significantly different in comparison to **8a** or **8b**, respectively; *p* < 0.05. Statistical analysis was performed using STATISTICA version 10 (StatSoft Inc., Palo Alto, CA, USA). *t*-test was used in the analysis.

**Table 2:** Resistance index (RI) values of phenolic-phospholipids.

Compounds	Acyl Residue		
	*sn*-1	*sn*-2	RI
**1a (ANISA)**	-	-	-
**3a**	ANISA	ANISA	-
**5a**	PA	ANISA	1.11
**7a**	ANISA	-	1.61
**8a**	ANISA	PA	0.99
**1b (VA)**	-	-	-
**3b**	VA	VA	0.42
**5b**	PA	VA	1.02
**7b**	VA	-	2.86
**8b**	VA	PA	1.04
DOX	-	-	55.81

DOX—doxorubicin. **RI** was calculated according to the formula RI = (IC_50_ estimated against resistant cell line) / (IC_50_ estimated against non-resistant cell line); values range: 0 < RI < 2—indicate that the tested compound is able to overcome drug resistance; 2 < RI < 10—defines the moderate ability of the compound to overcome drug resistance; RI > 10—defines no influence on the drug resistance phenomenon.

## Supplementary Materials

The following are available online, Figures S1–S40.
