# Supplementary material for: Synthesis, Characterization, and In Vitro Cancer Cell Growth Inhibition Evaluation of Novel Phosphatidylcholines with Anisic and Veratric Acids
Source: Molecules. 2018 Aug 13;23(8):2022. doi: 10.3390/molecules23082022 (PMC6222722; doi:10.3390/molecules23082022)

Supplementary Materials

Synthesis, characterization and in vitro cancer cell growth inhibition evaluation of novel phosphatidylcholines with anisic and veratric acids

Marta Czarnecka [1,*], Marta Świtalska [2], Joanna Wietrzyk [2], Gabriela Maciejewska [3], Anna Gliszczyńska [1,*]

[1] Department of Chemistry, Wrocław University of Environmental and Life Sciences, Norwida 25, 50-375 Wrocław, Poland
[2] Ludwik Hirszfeld Institute of Immunology and Experimental Therapy, Polish Academy of Sciences, Department of Experimental Oncology, Weigla 12, 53-114 Wrocław, Poland
[3] Central Laboratory of the Instrumental Analysis, Wrocław University of Technology, Wybrzeże Wyspiańskiego 27, Wrocław 50-370, Poland

* Correspondence: anna.gliszczynska@wp.pl, marta.b.czarnecka@gmail.com; Tel.: +48-71-320-5183

Content

Figure S1: ^{1}H NMR spectrum of 3a. 4
Figure S2: ^{13}C NMR spectrum of 3a. 5
Figure S3: ^{31}P NMR spectrum of 3a. 6
Figure S4: ^{1}H – ^{1}H COSY spectrum of 3a. 7
Figure S5: HSQC spectrum of 3a. 8
Figure S6: ^{1}H NMR spectrum of 3b. 10
Figure S7: ^{13}C NMR spectrum of 3b. 11
Figure S8: ^{31}P NMR spectrum of 3b. 12
Figure S9: ^{1}H – ^{1}H COSY spectrum of 3b. 13
Figure S10: HSQC spectrum of 3b. 14
Figure S11: ^{1}H NMR spectrum of 5a. 16
Figure S12: ^{13}C NMR spectrum of 5a. 17
Figure S13: ^{31}P NMR spectrum of 5a. 18
Figure S14: ^{1}H – ^{1}H COSY spectrum of 5a. 19
Figure S15: HSQC spectrum of 5a. 20
Figure S16: ^{1}H NMR spectrum of 5b. 22
Figure S17: ^{13}C NMR spectrum of 5b. 23
Figure S18: ^{31}P NMR spectrum of 5b. 24

Figure S19: ^{1}H – ^{1}H COSY spectrum of 5b. 25
Figure S20: HSQC spectrum of 5b. 26
Figure S21: ^{1}H NMR spectrum of 7a. 28
Figure S22: ^{13}C NMR spectrum of 7a. 29
Figure S23: ^{31}P NMR spectrum of 7a. 30
Figure S24: ^{1}H – ^{1}H COSY spectrum of 7a. 31
Figure S25: HSQC spectrum of 7a. 32
Figure S26: ^{1}H NMR spectrum of 7b. 34
Figure S27: ^{13}C NMR spectrum of 7b. 35
Figure S28: ^{31}P NMR spectrum of 7b. 36
Figure S29: ^{1}H – ^{1}H COSY spectrum of 7b. 37
Figure S30: HSQC spectrum of 7b. 38
Figure S31: ^{1}H NMR spectrum of 8a. 40
Figure S32: ^{13}C NMR spectrum of 8a. 41
Figure S33: ^{31}P NMR spectrum of 8a. 42
Figure S34: ^{1}H – ^{1}H COSY spectrum of 8a. 43
Figure S35: HSQC spectrum of 8a. 44
Figure S36: ^{1}H NMR spectrum of 8b. 46
Figure S37: ^{13}C NMR spectrum of 8b. 47
Figure S38: ^{31}P NMR spectrum of 8b. 48
Figure S39: ^{1}H – ^{1}H COSY spectrum of 8b. 49
Figure S40: HSQC spectrum of 8b. 50

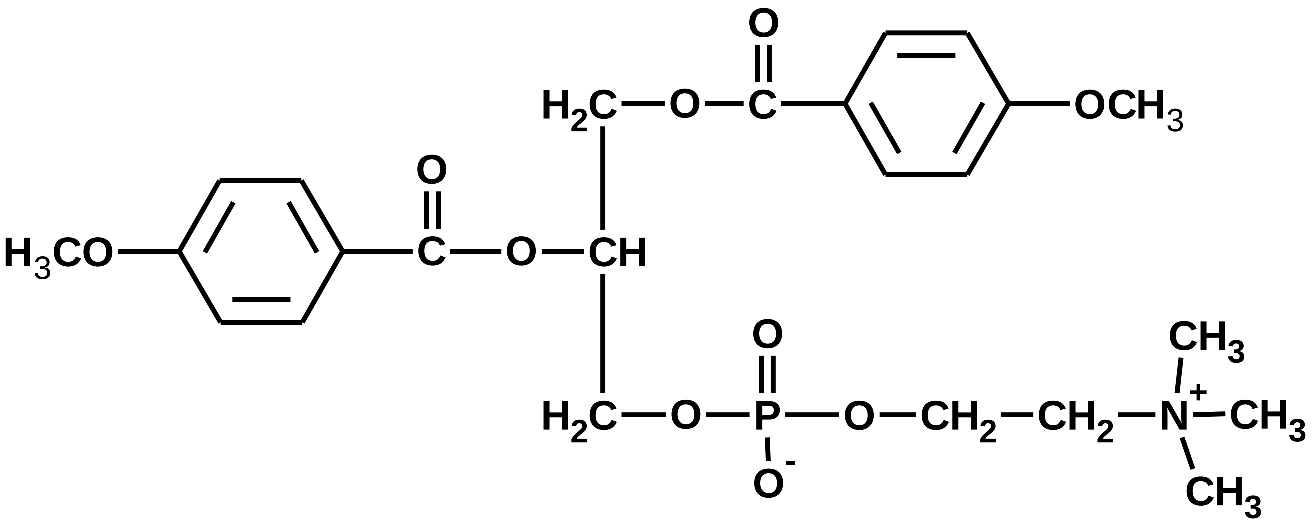

1,2-dianisoyl-*sn*-glycero-3-phosphocholine (**3a**)

Figure S1: ^{1}H NMR spectrum of 3a.

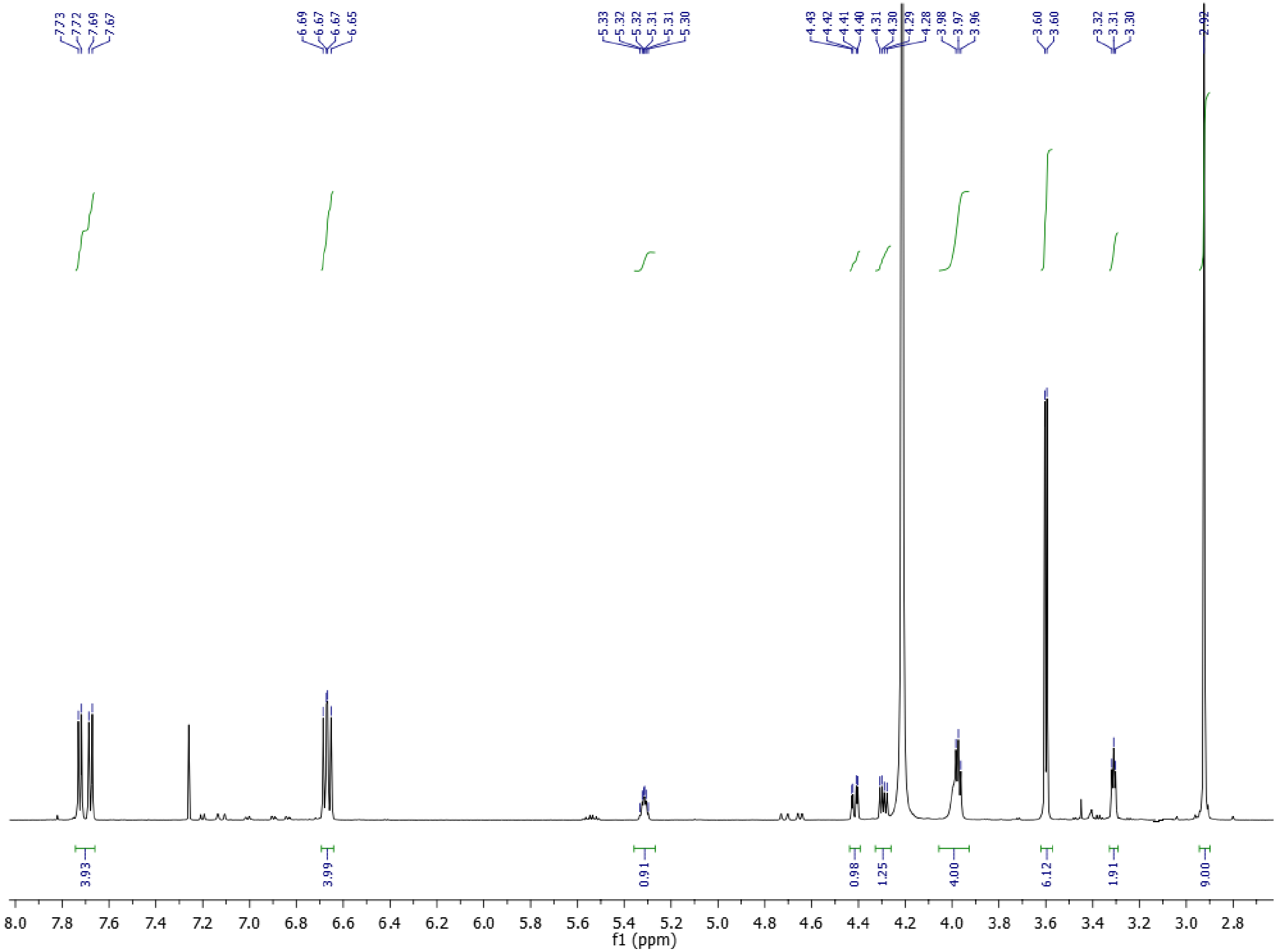

Figure S2: ^{13}C NMR spectrum of 3a.

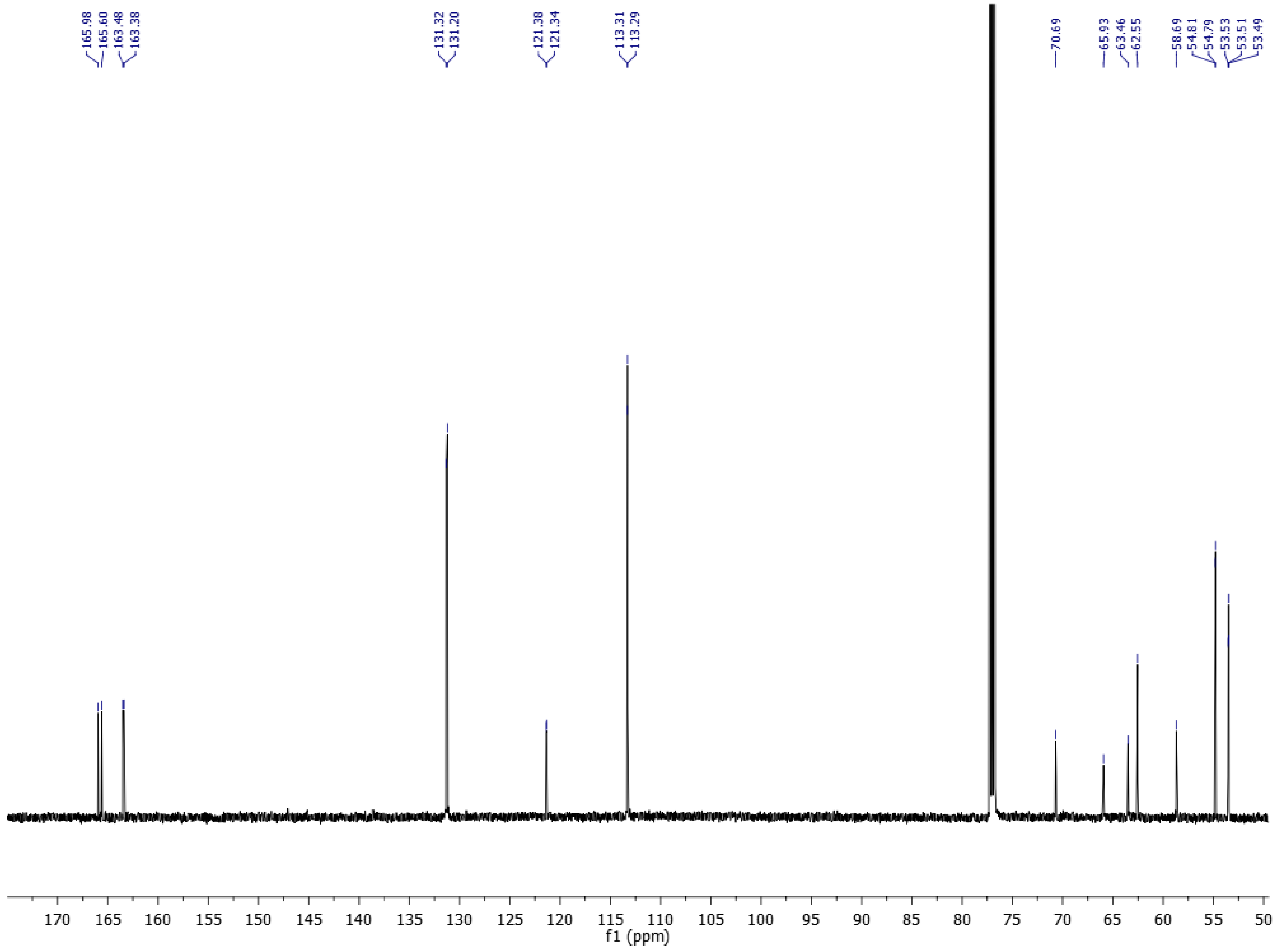

Figure S3: ^{31}P NMR spectrum of 3a.

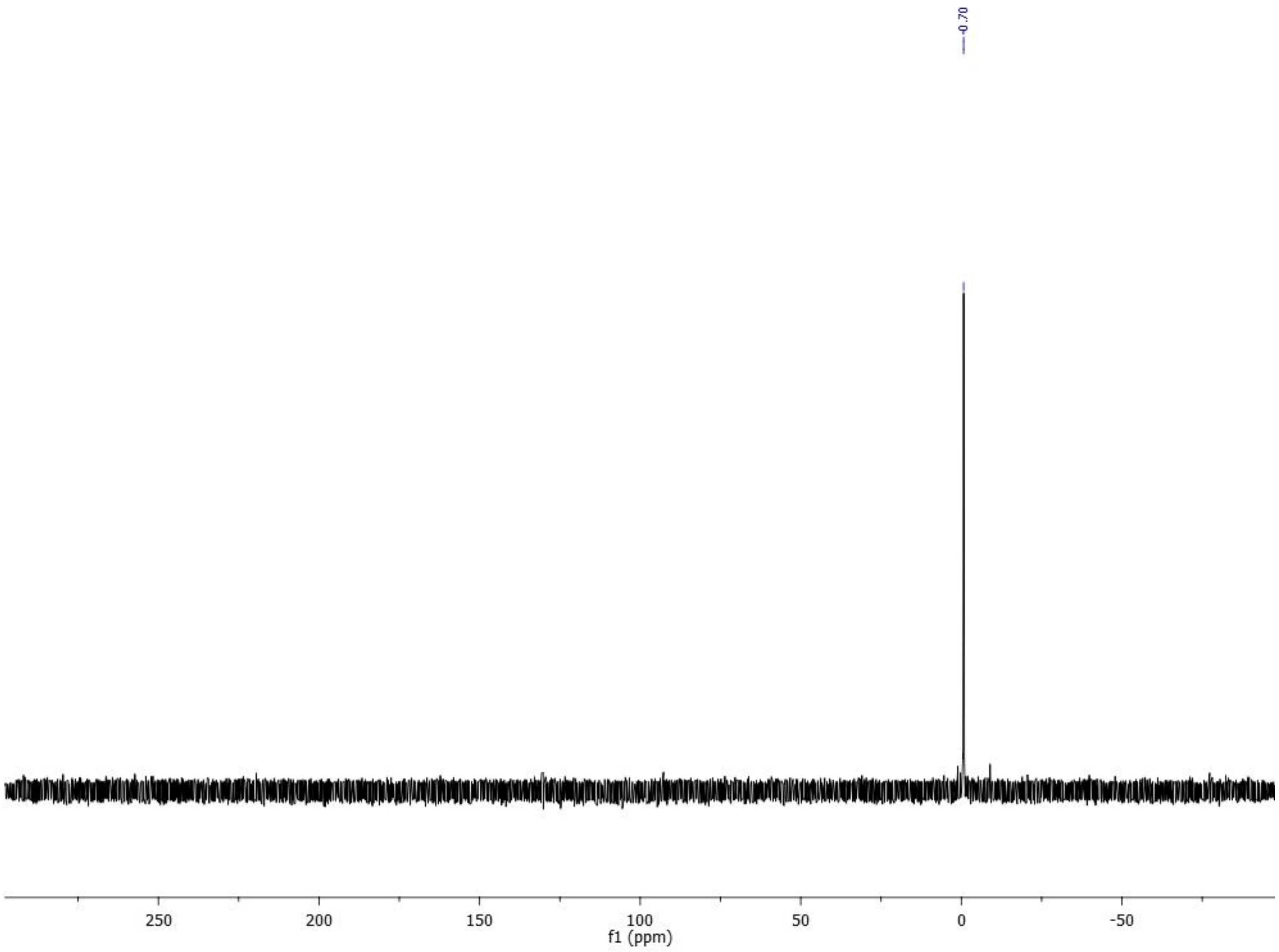

Figure S4: ^{1}H – ^{1}H COSY spectrum of 3a.

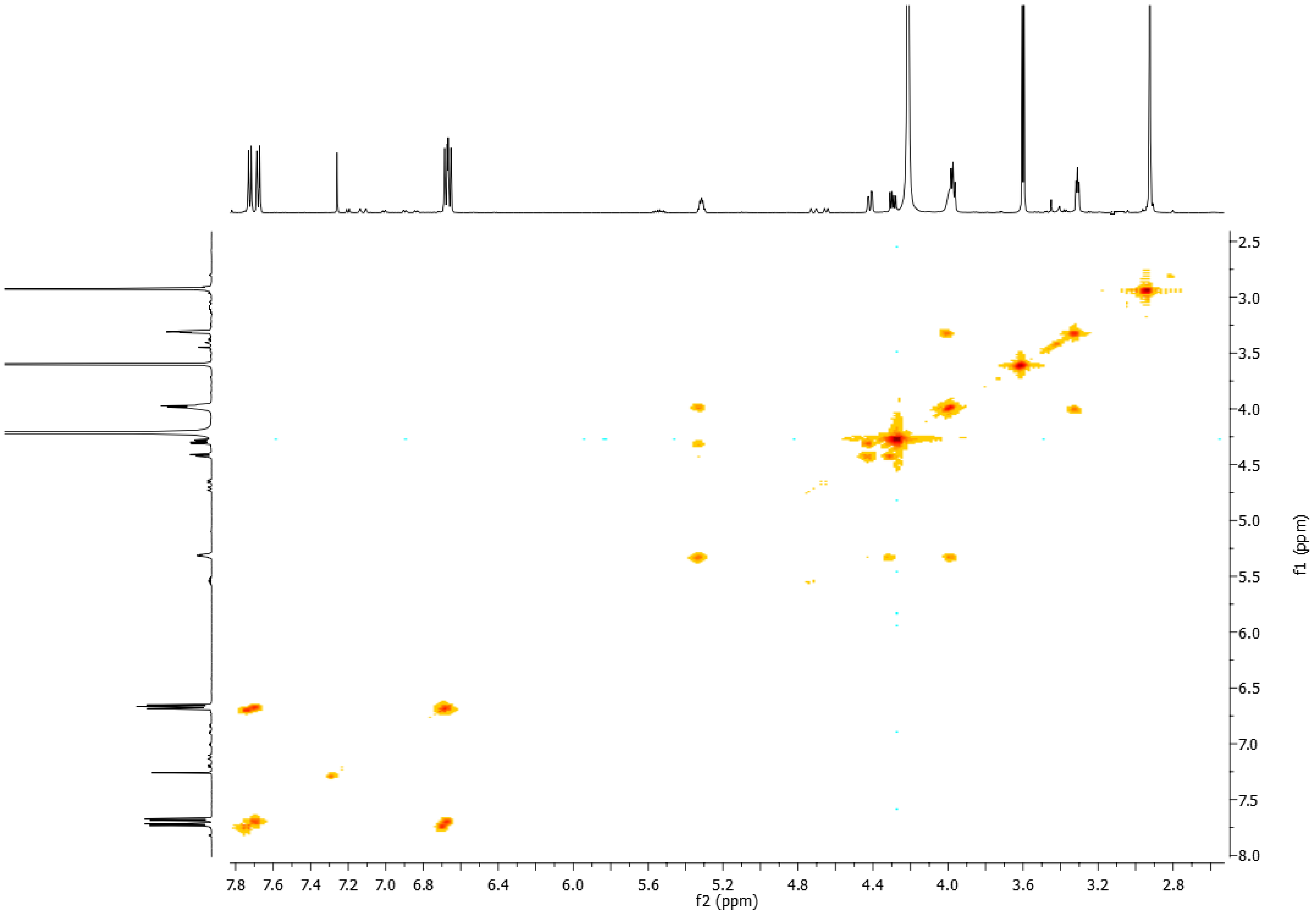

Figure S5: HSQC spectrum of 3a.

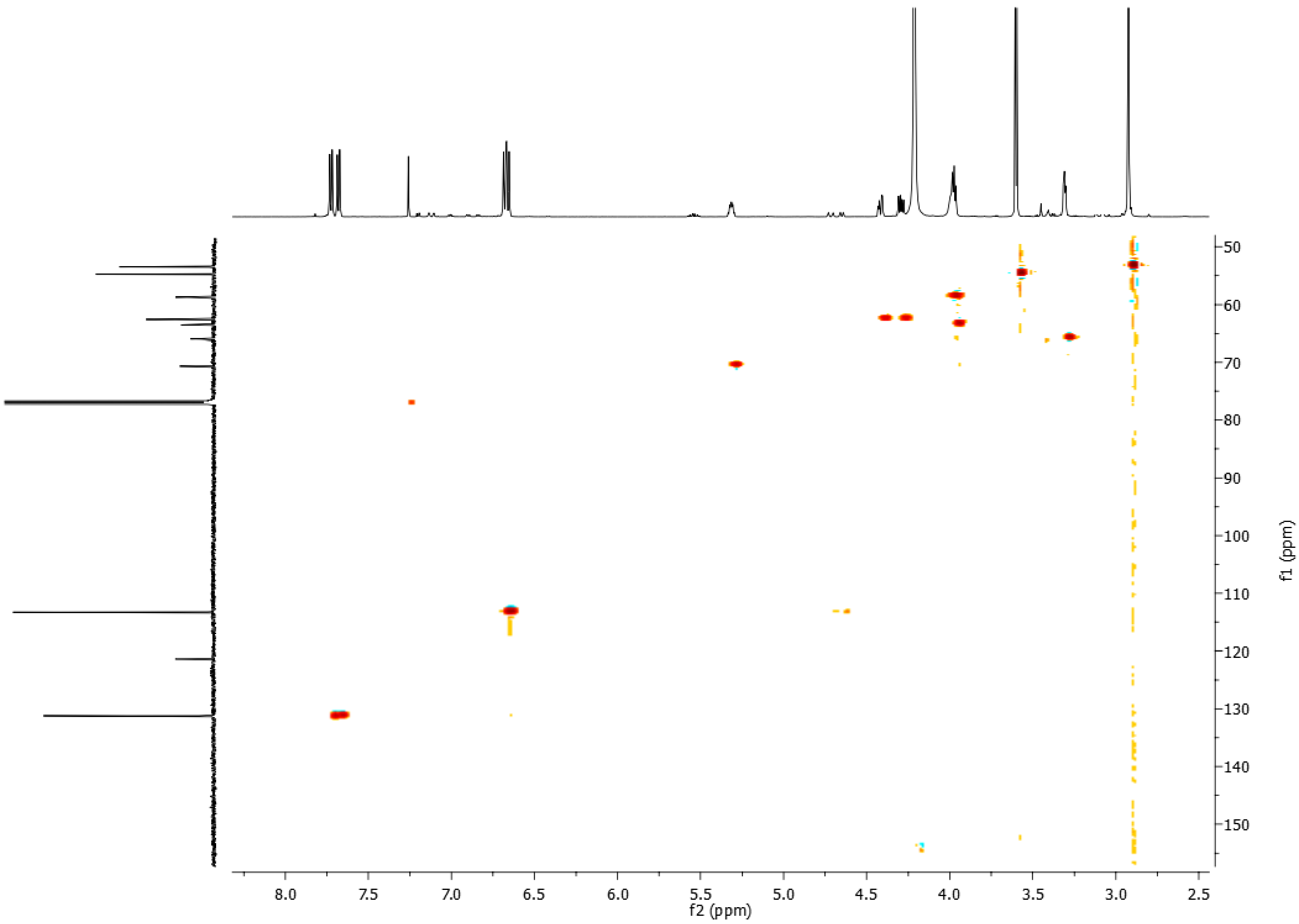

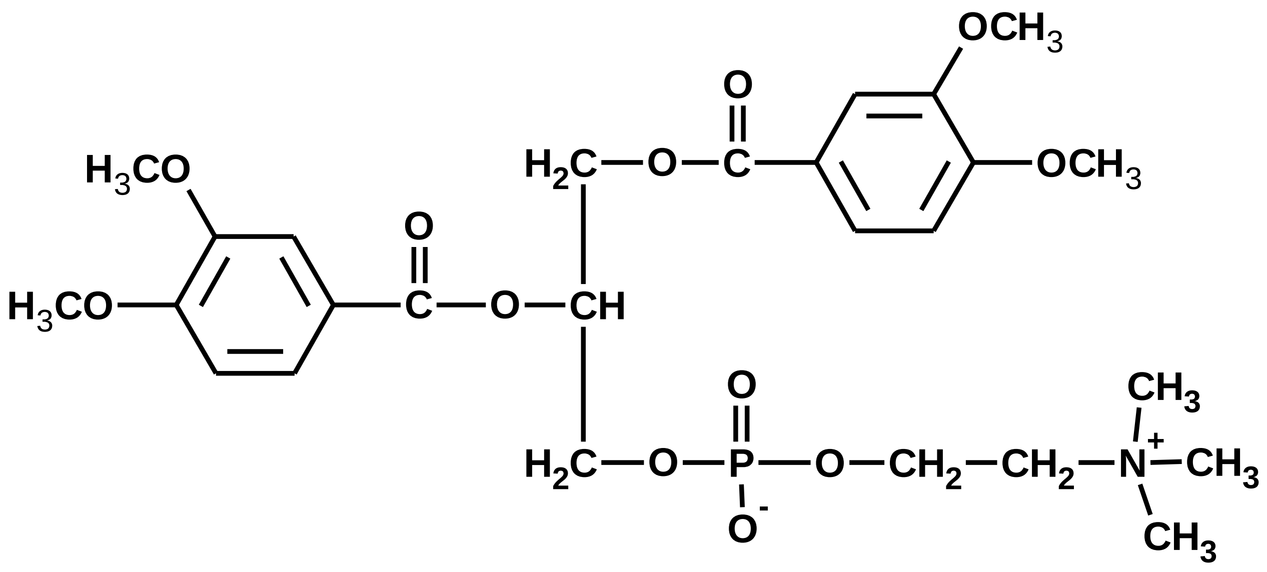

1,2-diveratroyl-*sn*-glycero-3-phosphocholine (**3b**)

Figure S6: ^{1}H NMR spectrum of 3b.

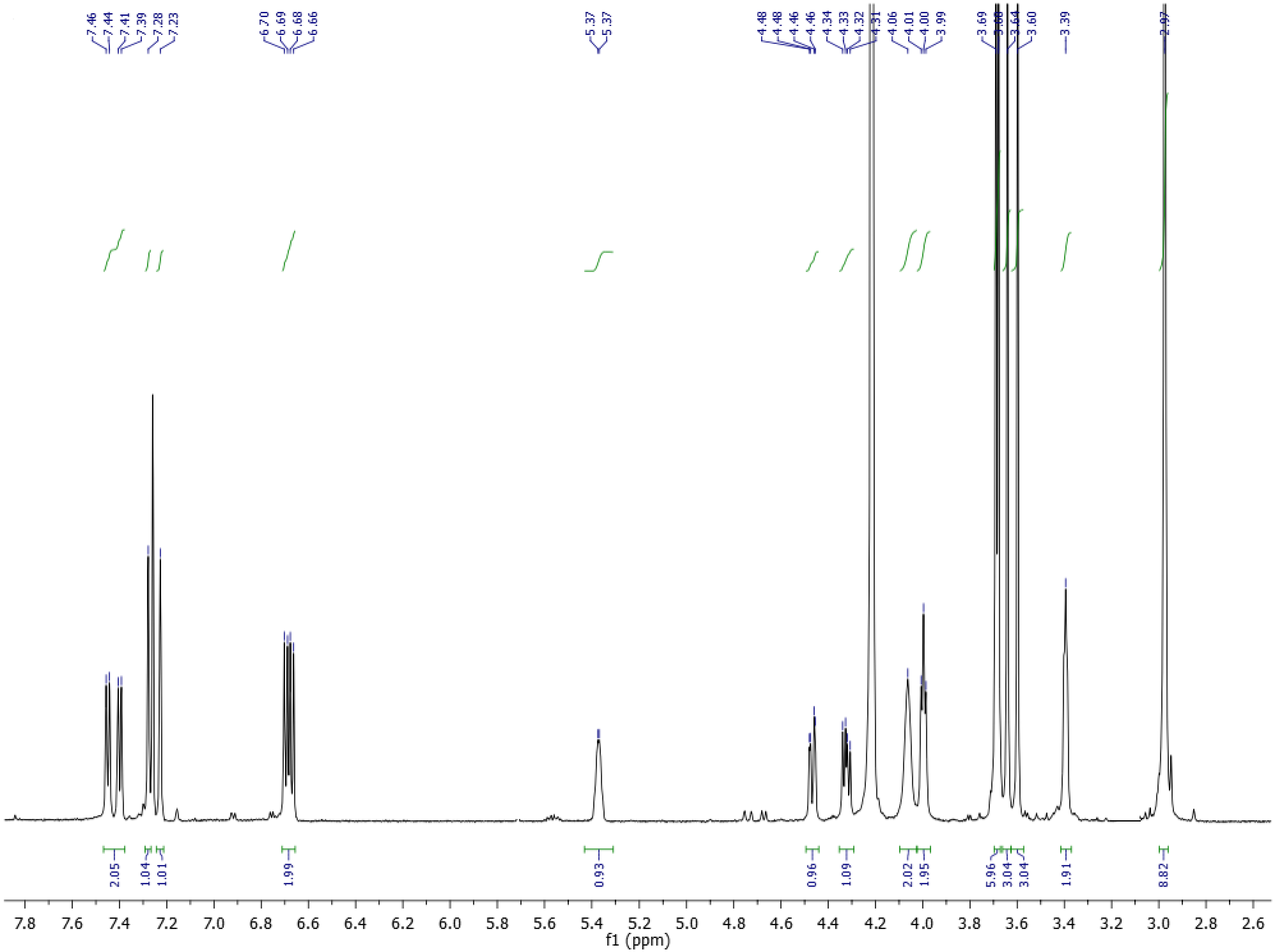

Figure S7: ^{13}C NMR spectrum of 3b.

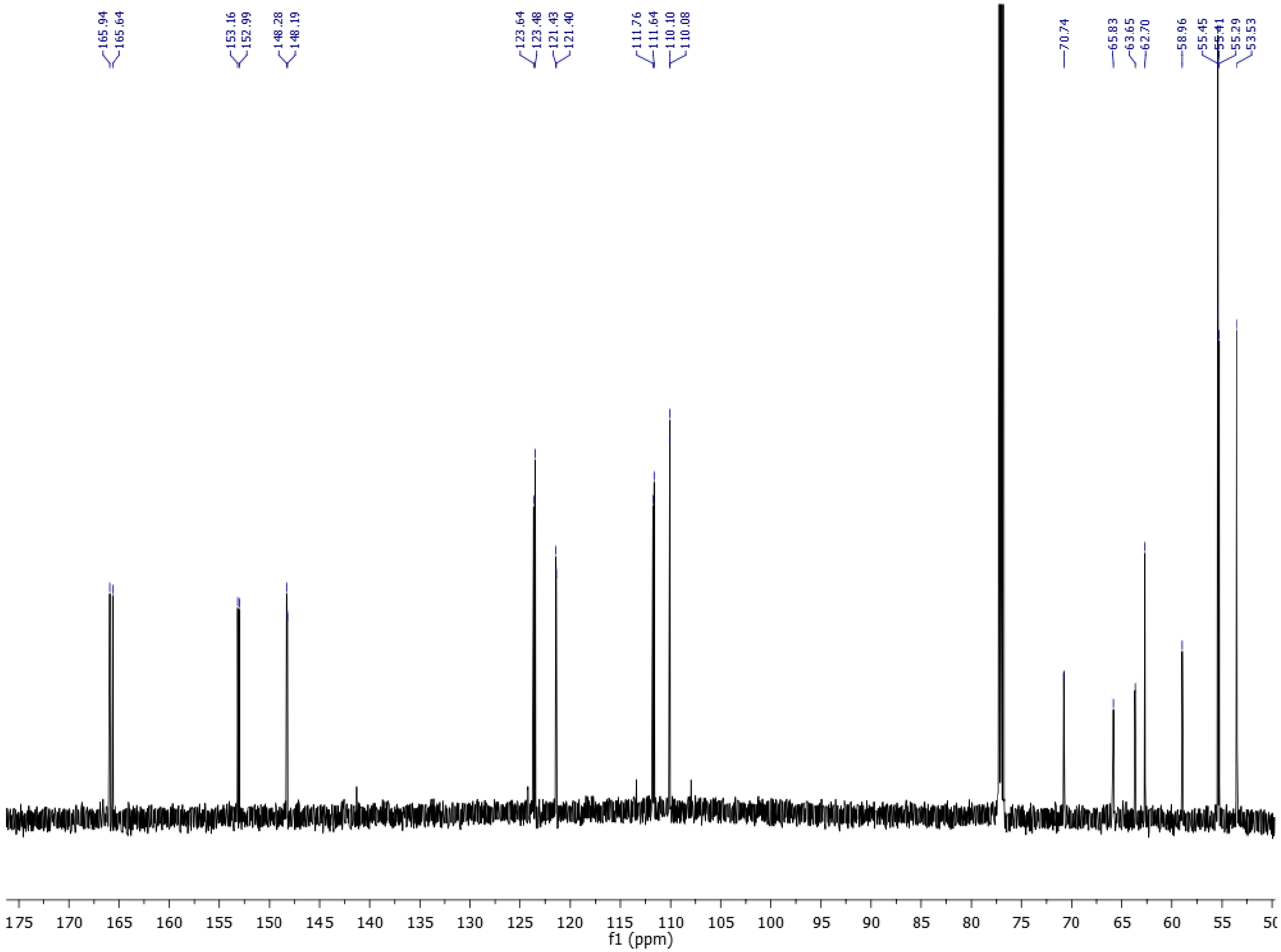

Figure S8: ^{31}P NMR spectrum of 3b.

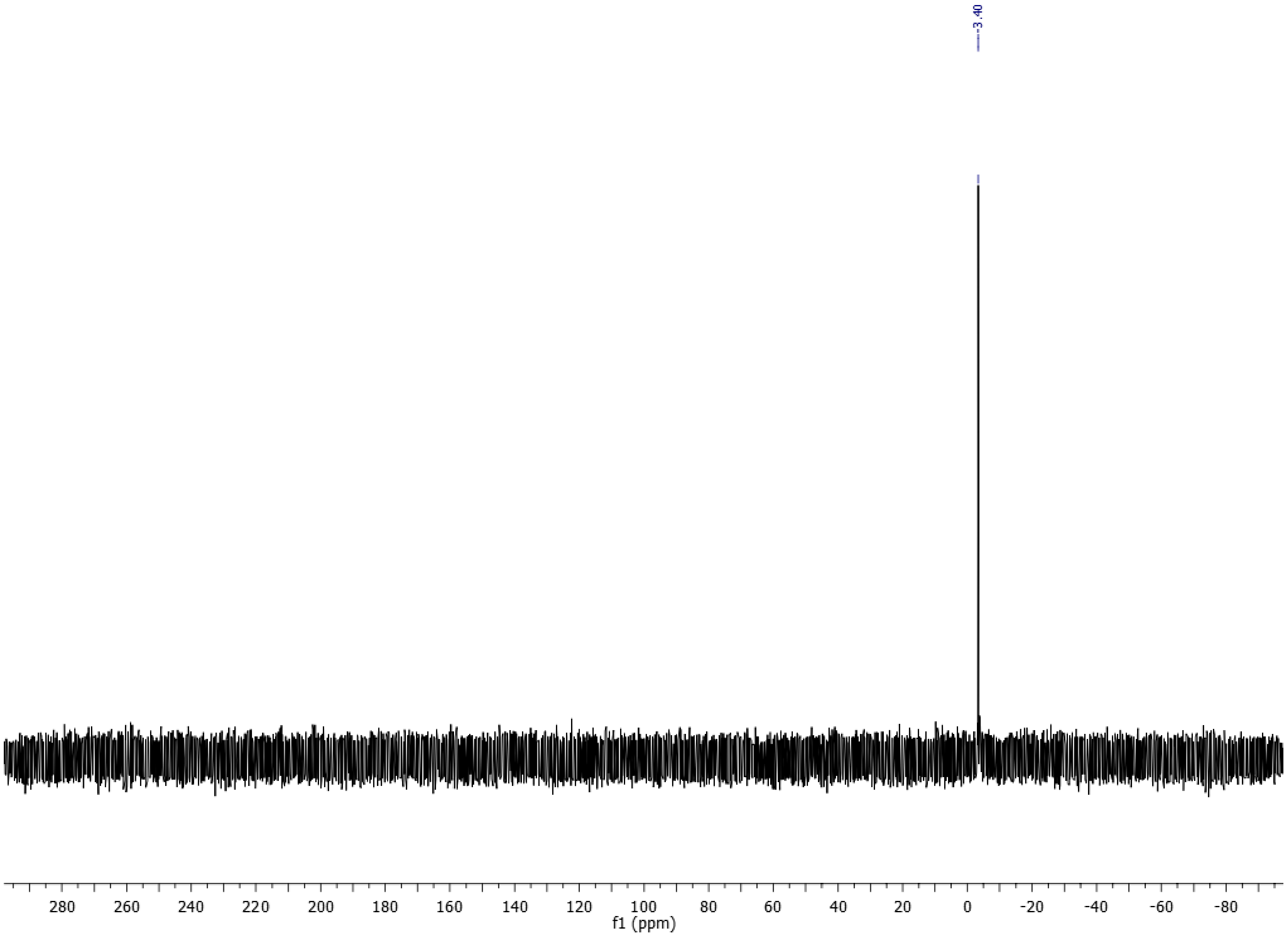

Figure S9: ^{1}H – ^{1}H COSY spectrum of 3b.

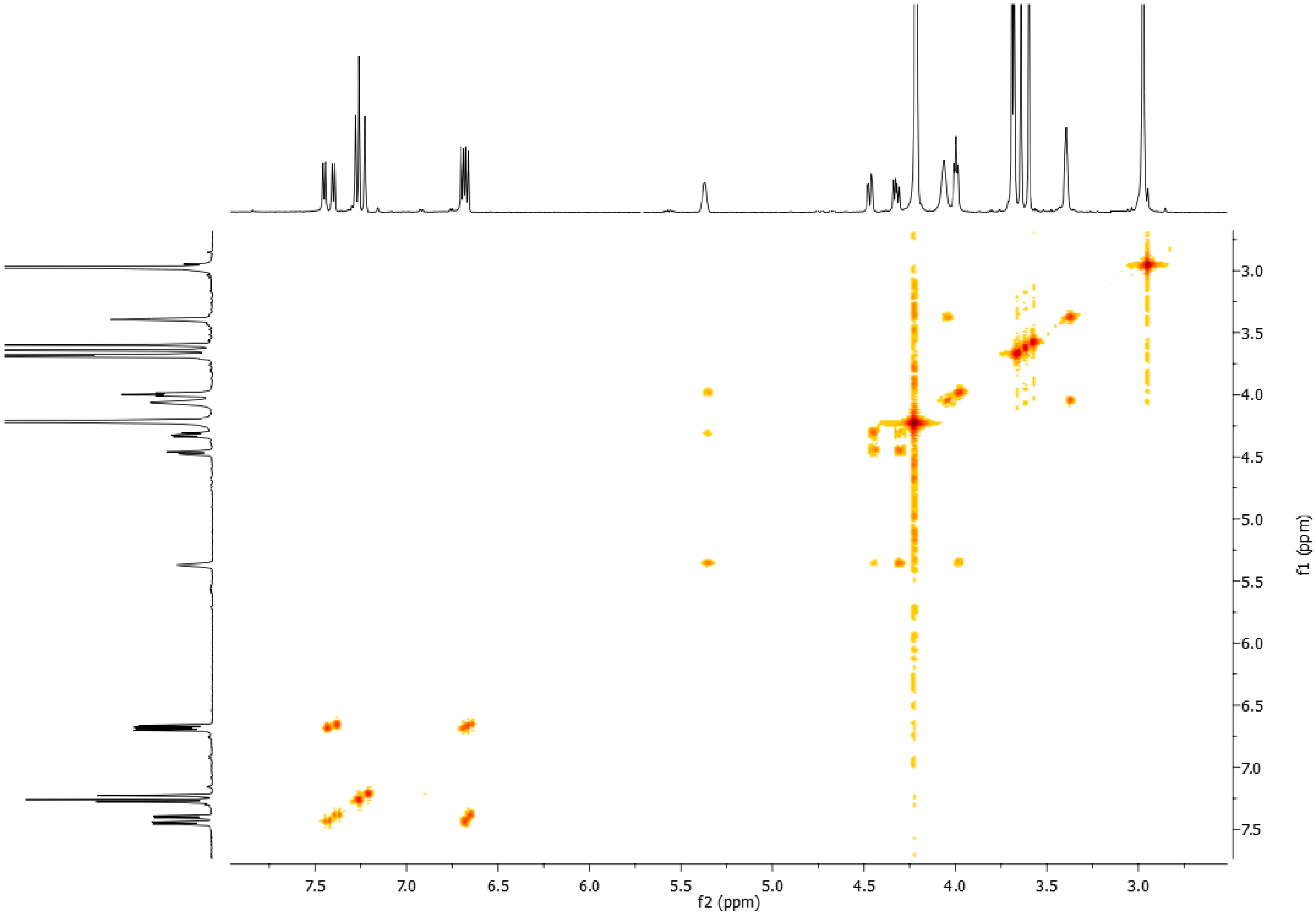

Figure S10: HSQC spectrum of 3b.

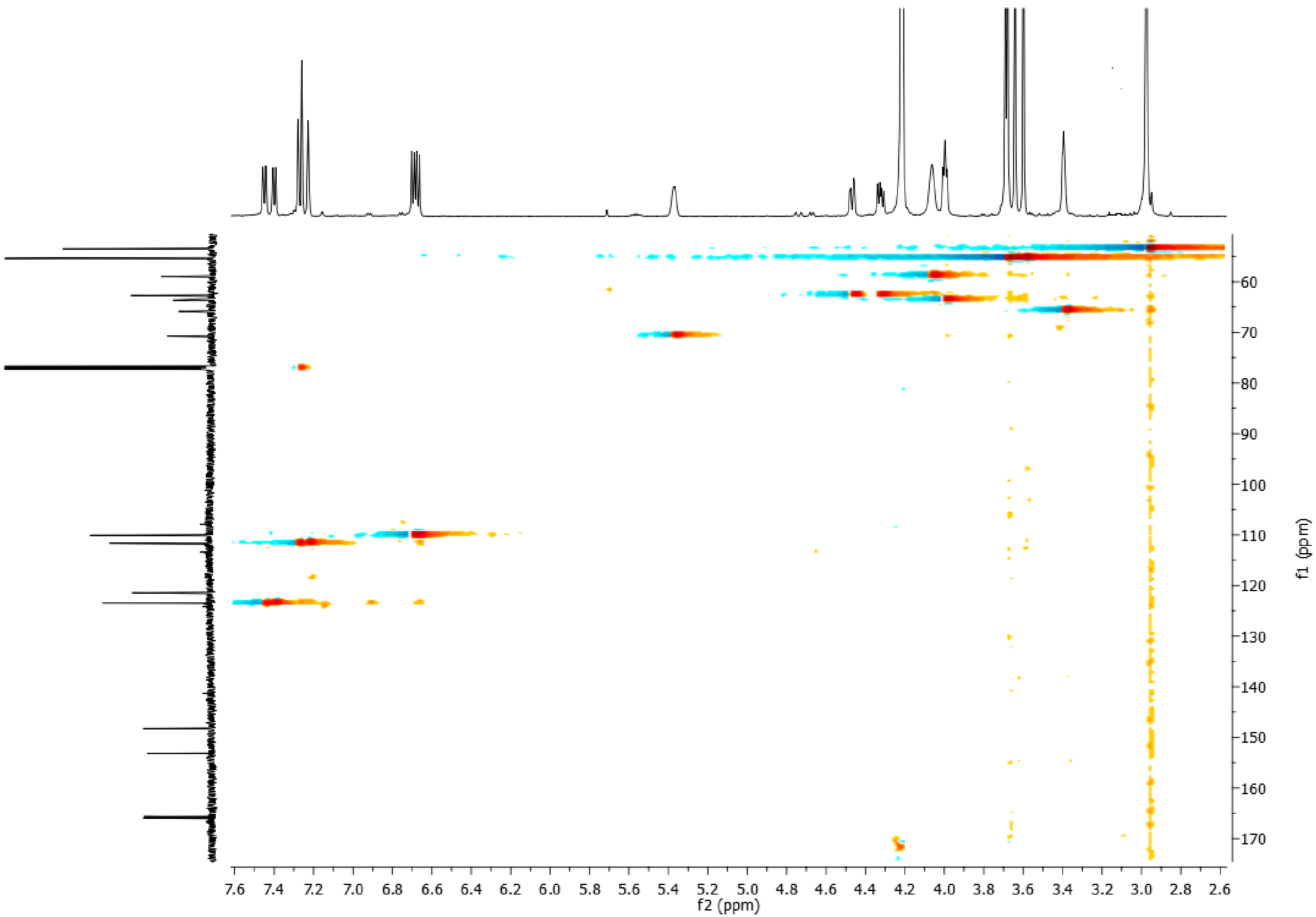

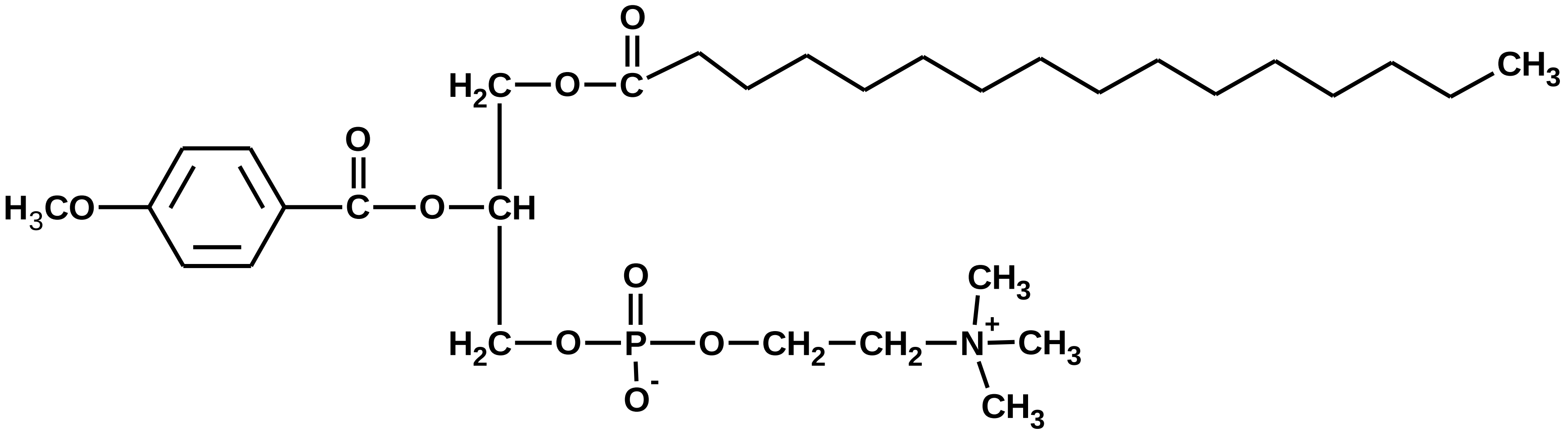

1-palmitoyl-2-anisoyl-*sn*-glycero-3-phosphocholine (**5a**)

Figure S11: ^{1}H NMR spectrum of 5a.

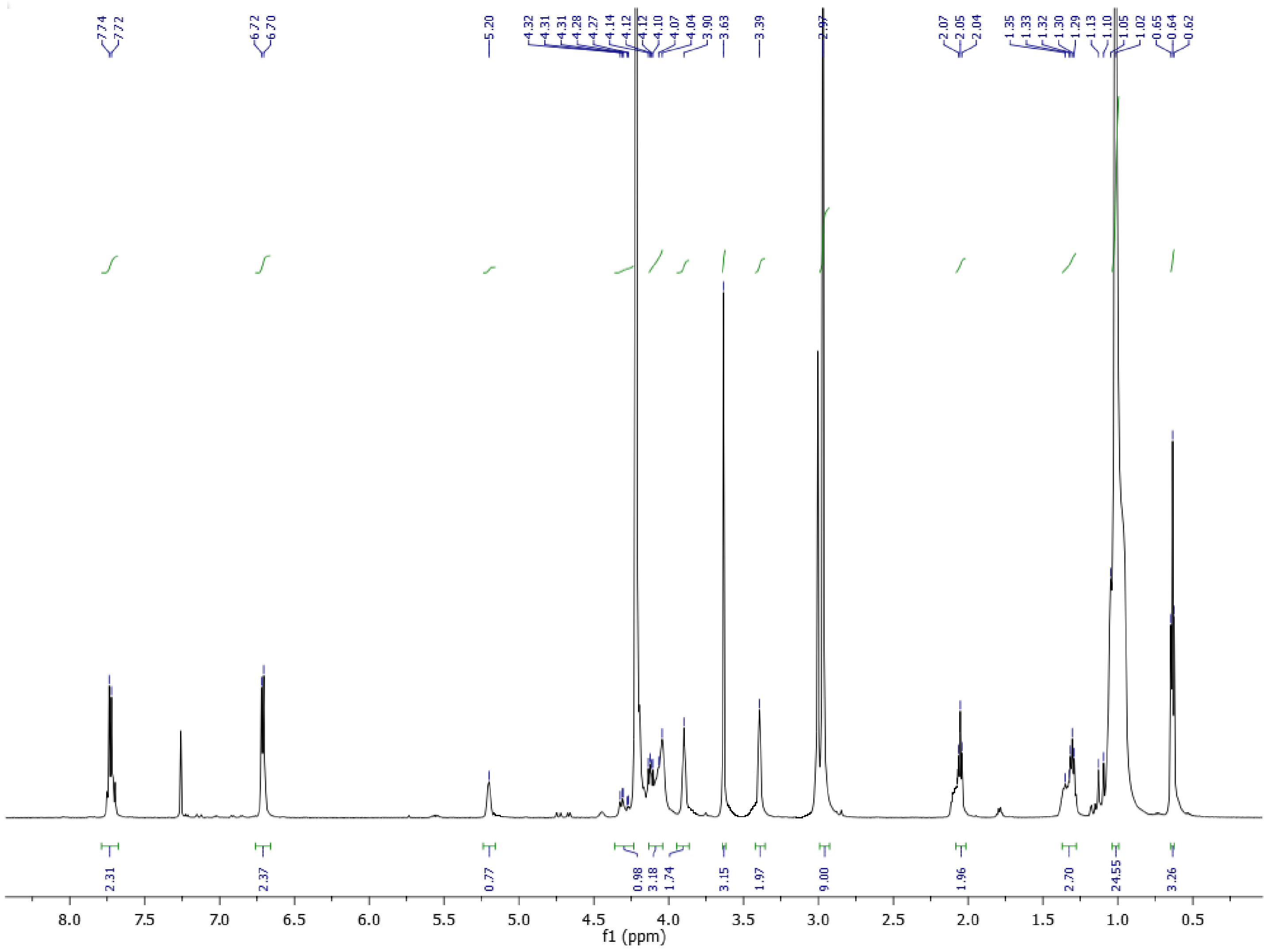

Figure S12: ^{13}C NMR spectrum of 5a.

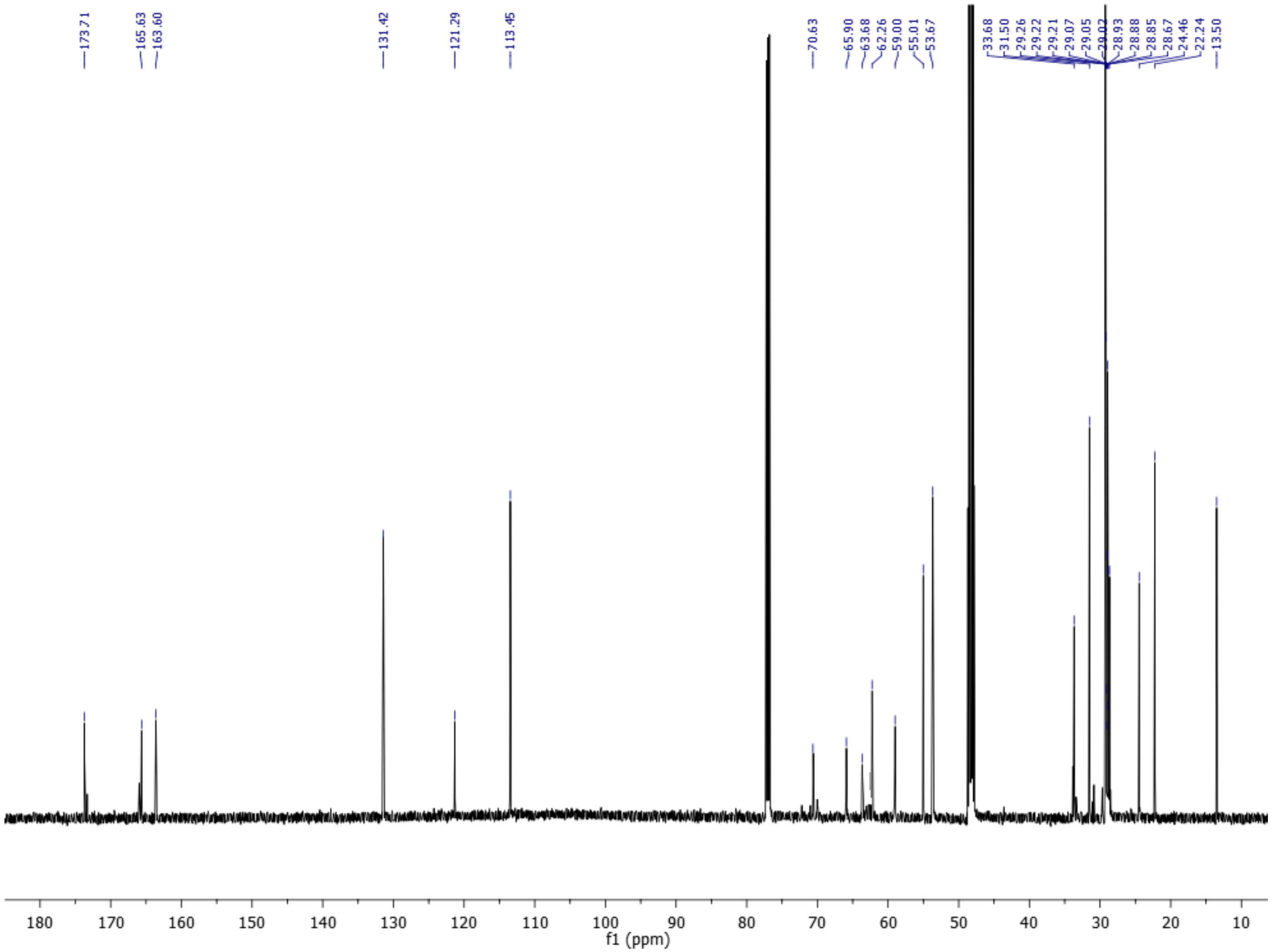

Figure S13: ^{31}P NMR spectrum of 5a.

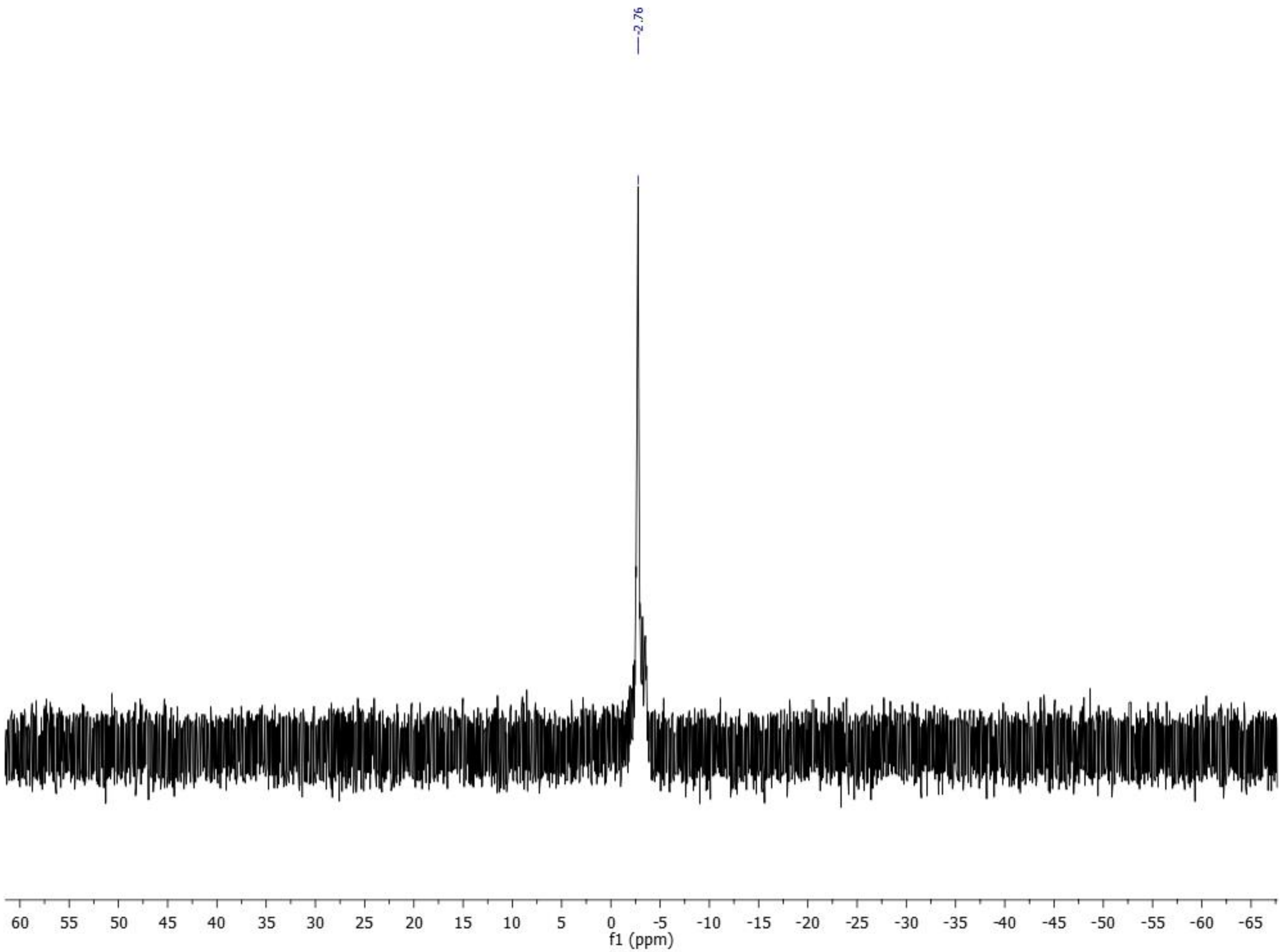

Figure S14: ^{1}H – ^{1}H COSY spectrum of 5a.

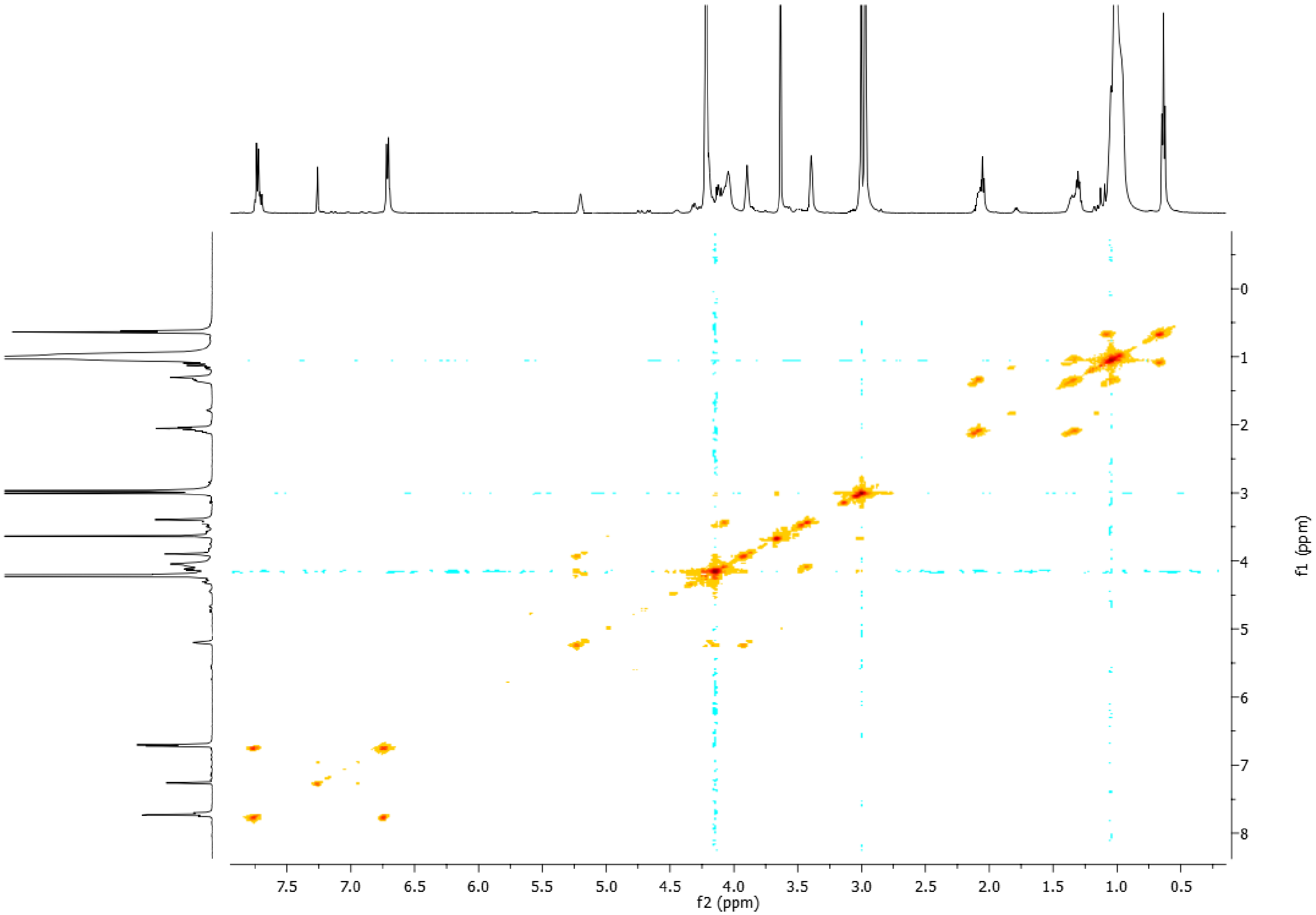

Figure S15: HSQC spectrum of 5a.

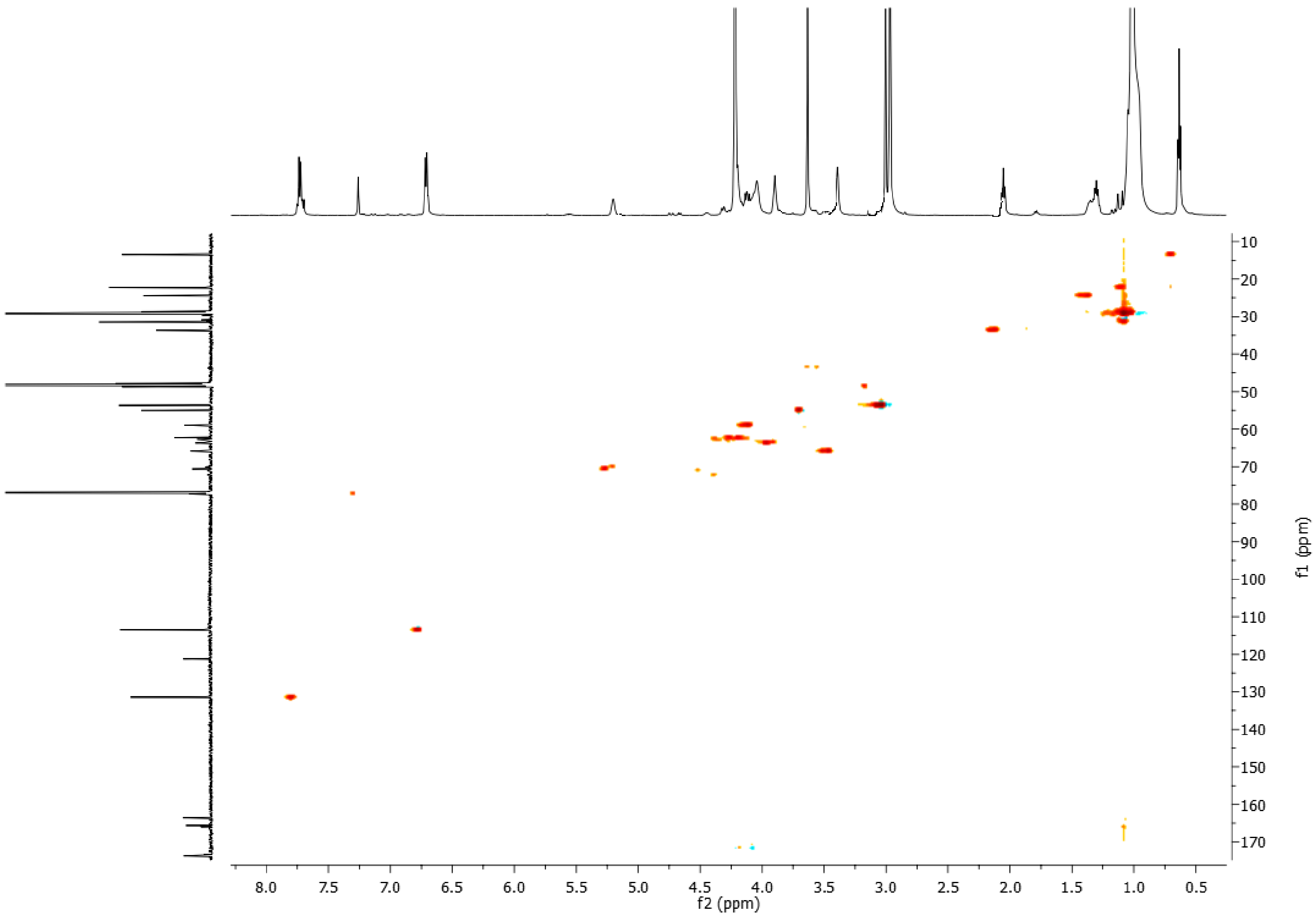

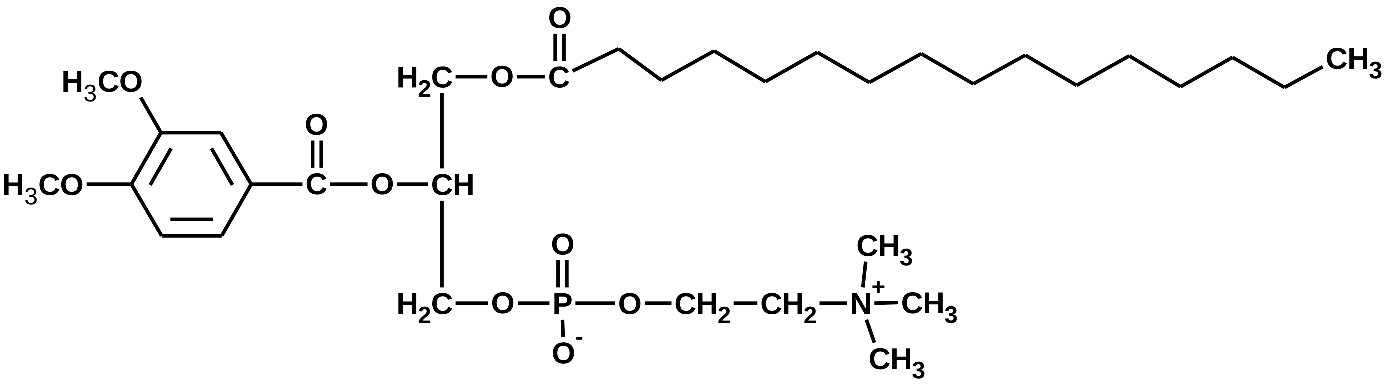

1-palmitoyl-2-veratroyl-*sn*-glycero-3-phosphocholine (**5b**)

Figure S16: ^{1}H NMR spectrum of 5b.

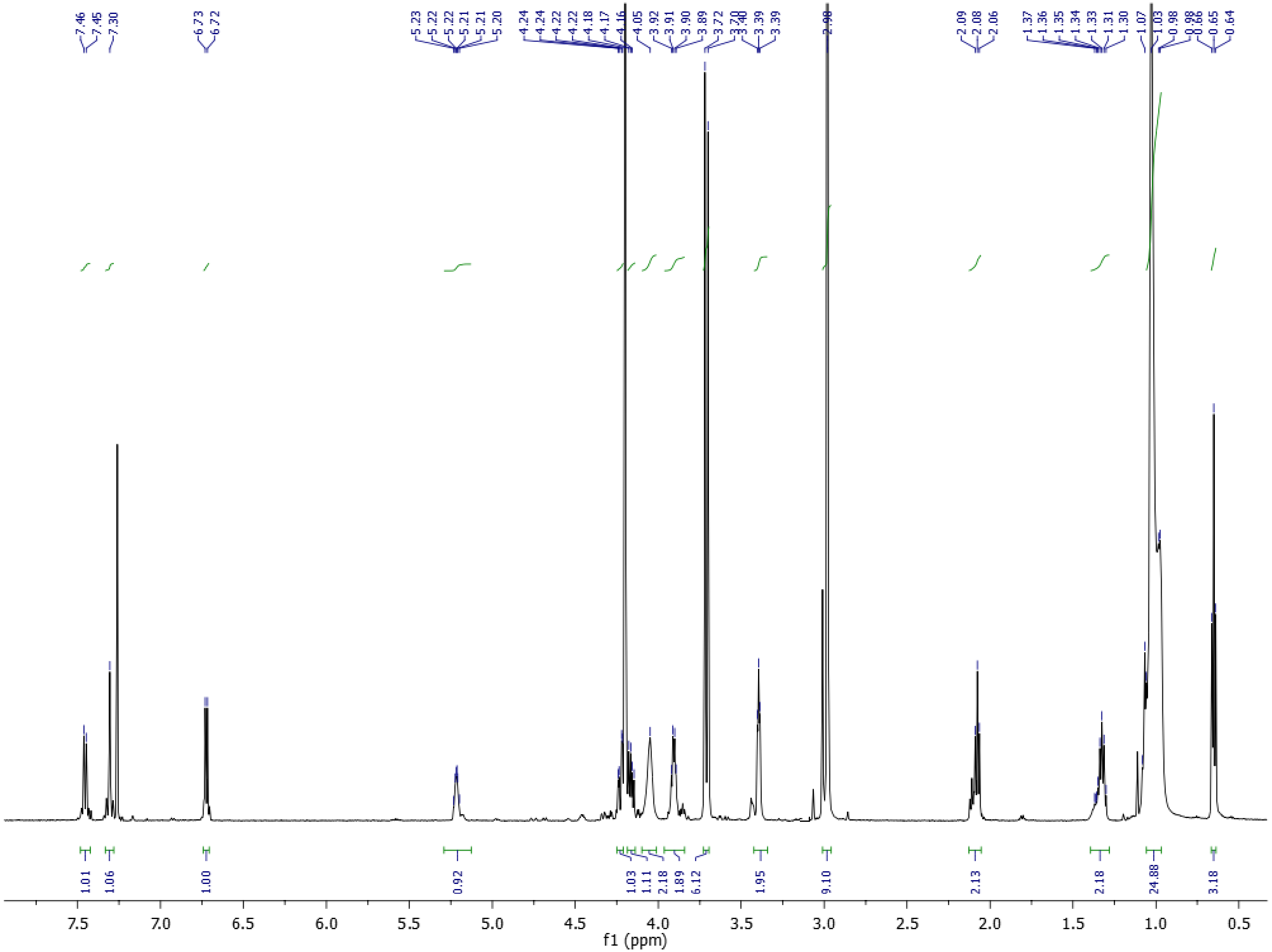

Figure S17: ^{13}C NMR spectrum of 5b.

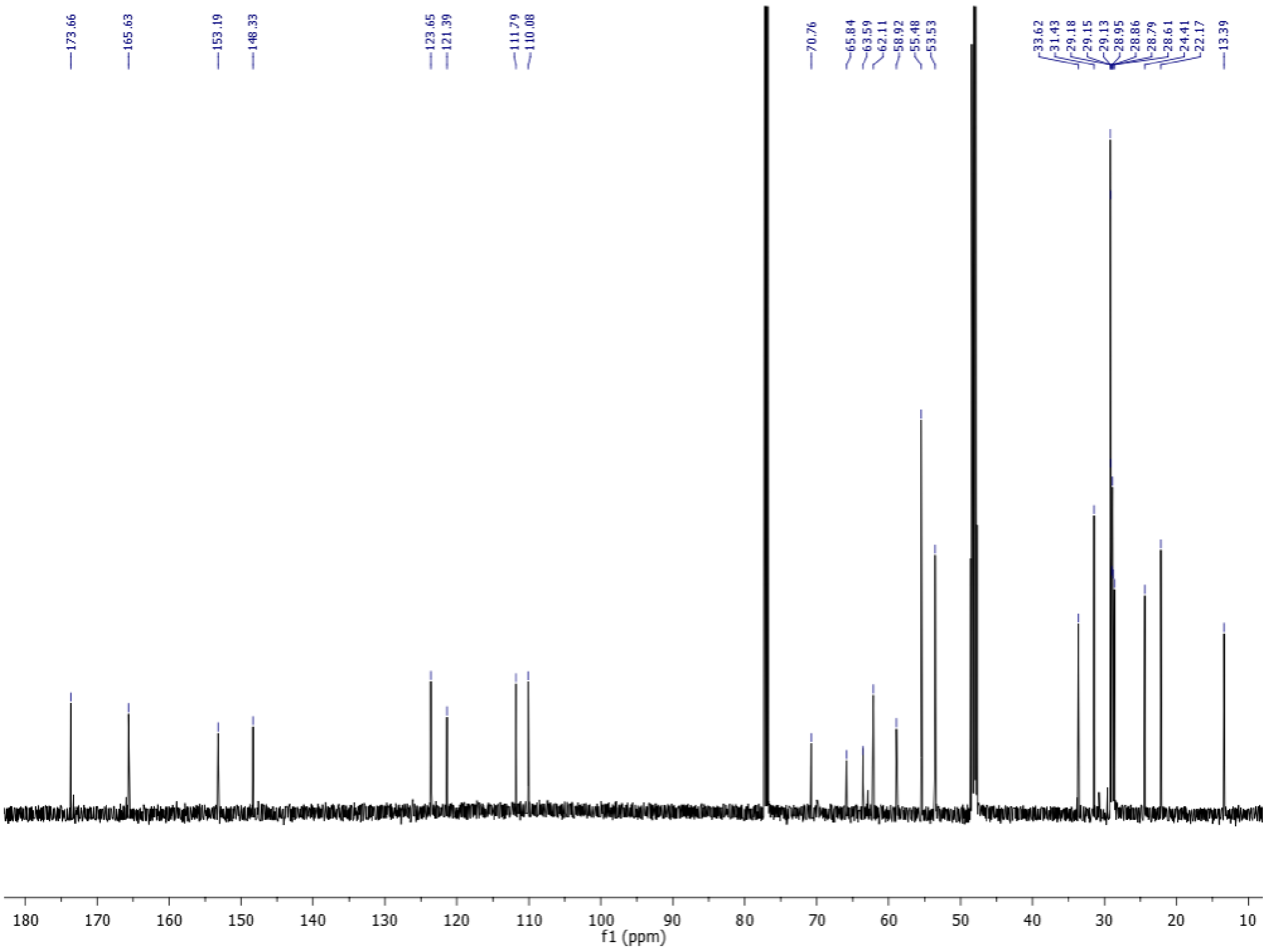

Figure S18: ^{31}P NMR spectrum of 5b.

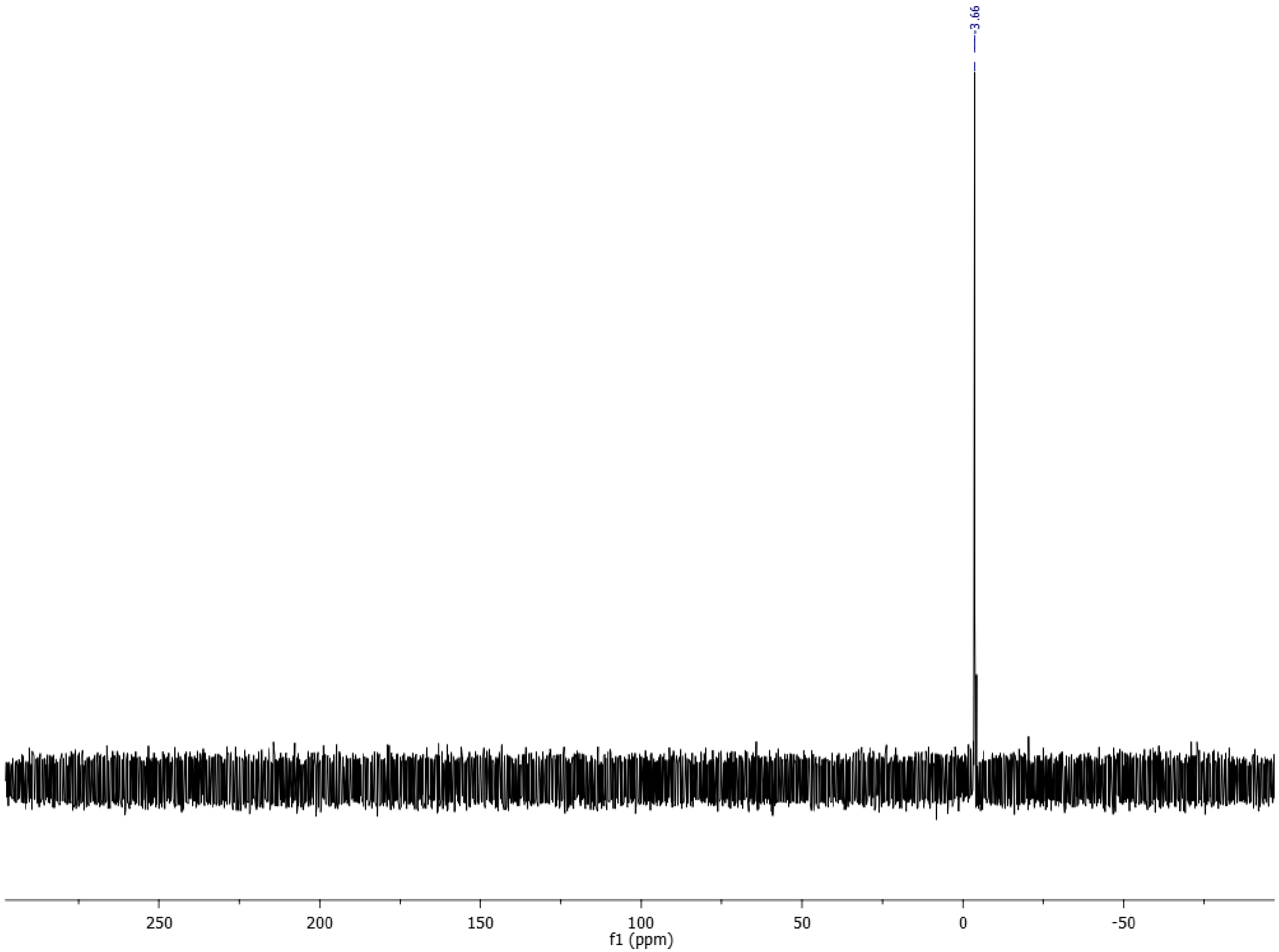

Figure S19: ^{1}H – ^{1}H COSY spectrum of 5b.

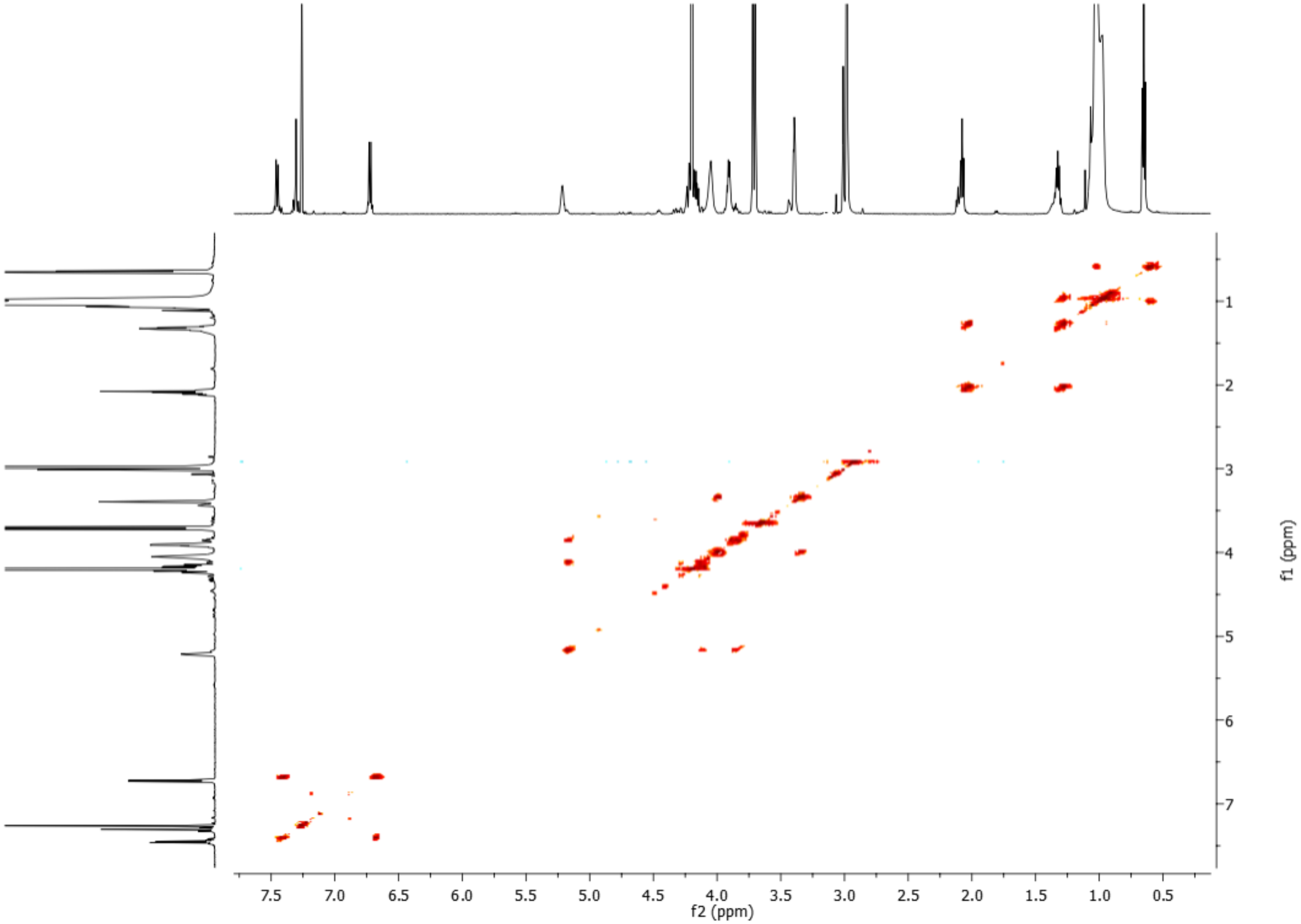

Figure S20: HSQC spectrum of 5b.

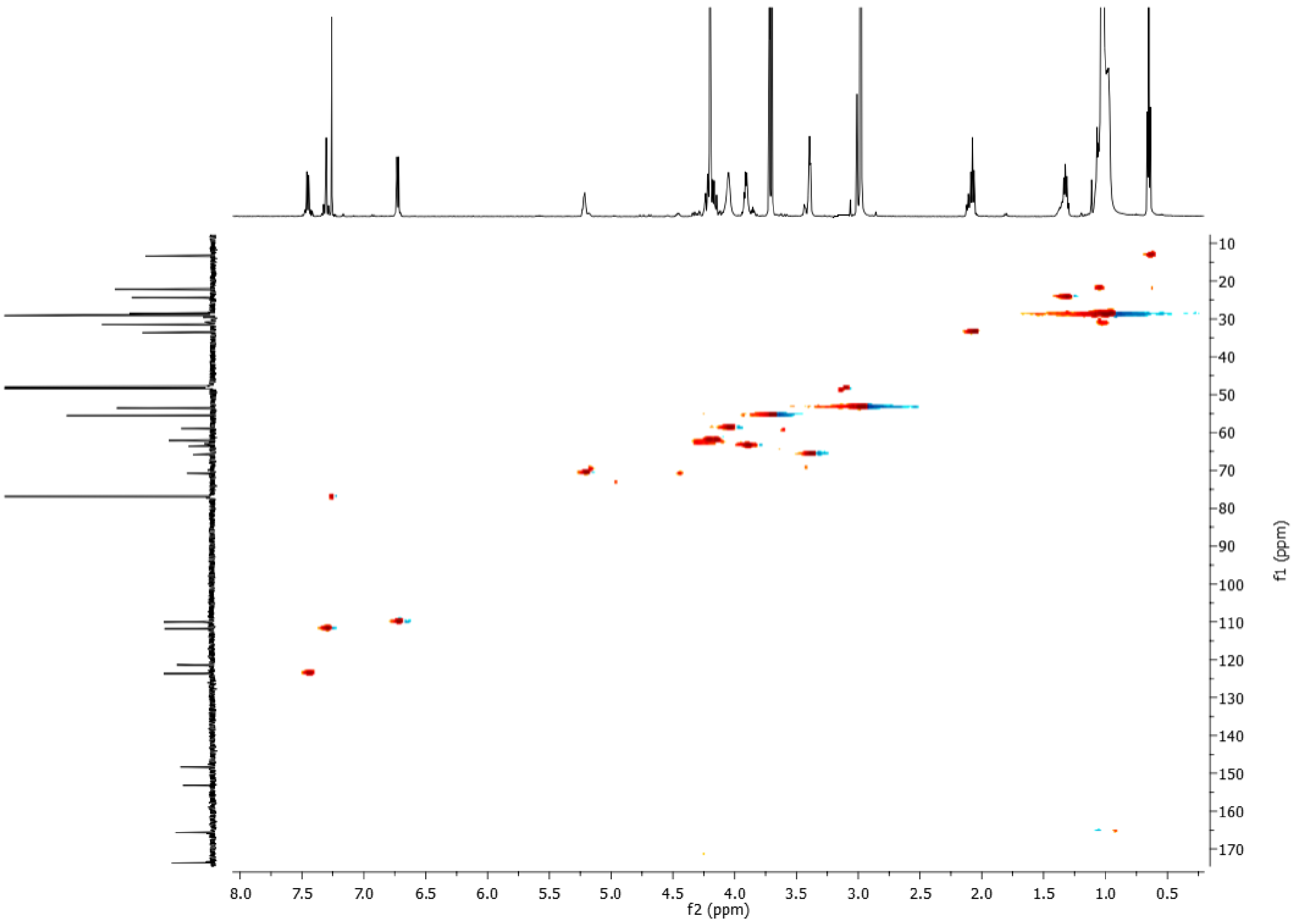

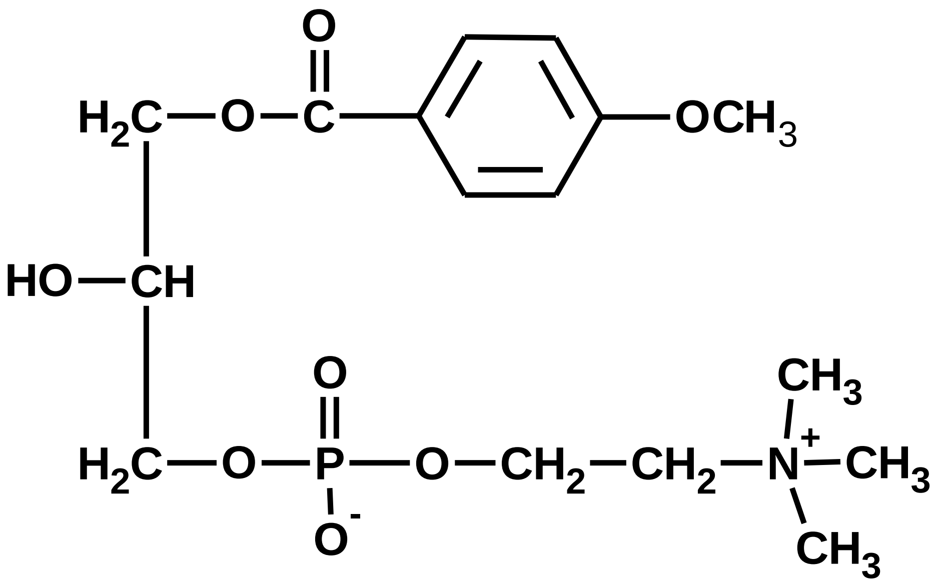

1-anisoyl-2-hydroxy-*sn*-glycero-3-phosphocholine (**7a**)

Figure S21: ^{1}H NMR spectrum of 7a.

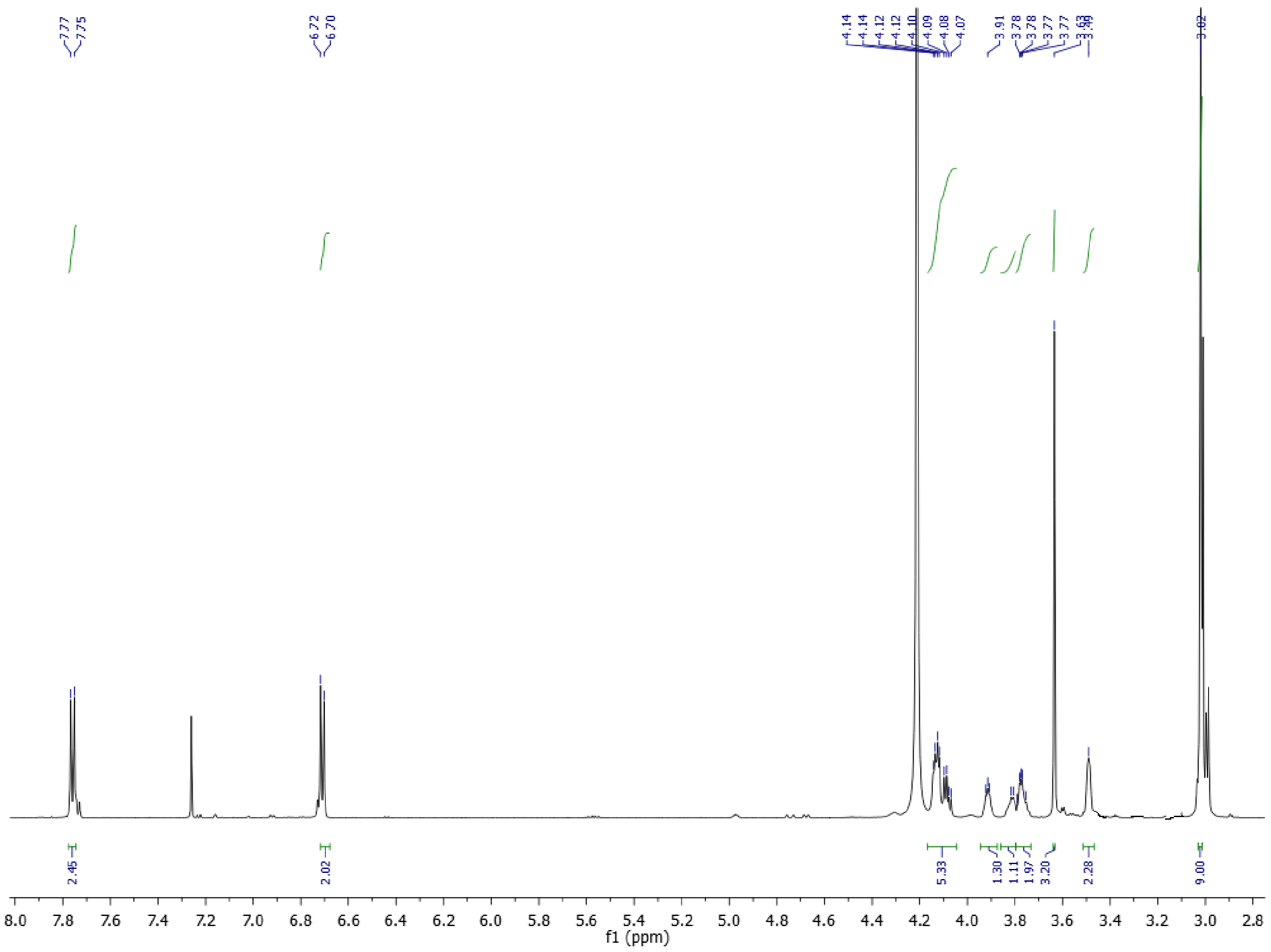

Figure S22: ^{13}C NMR spectrum of 7a.

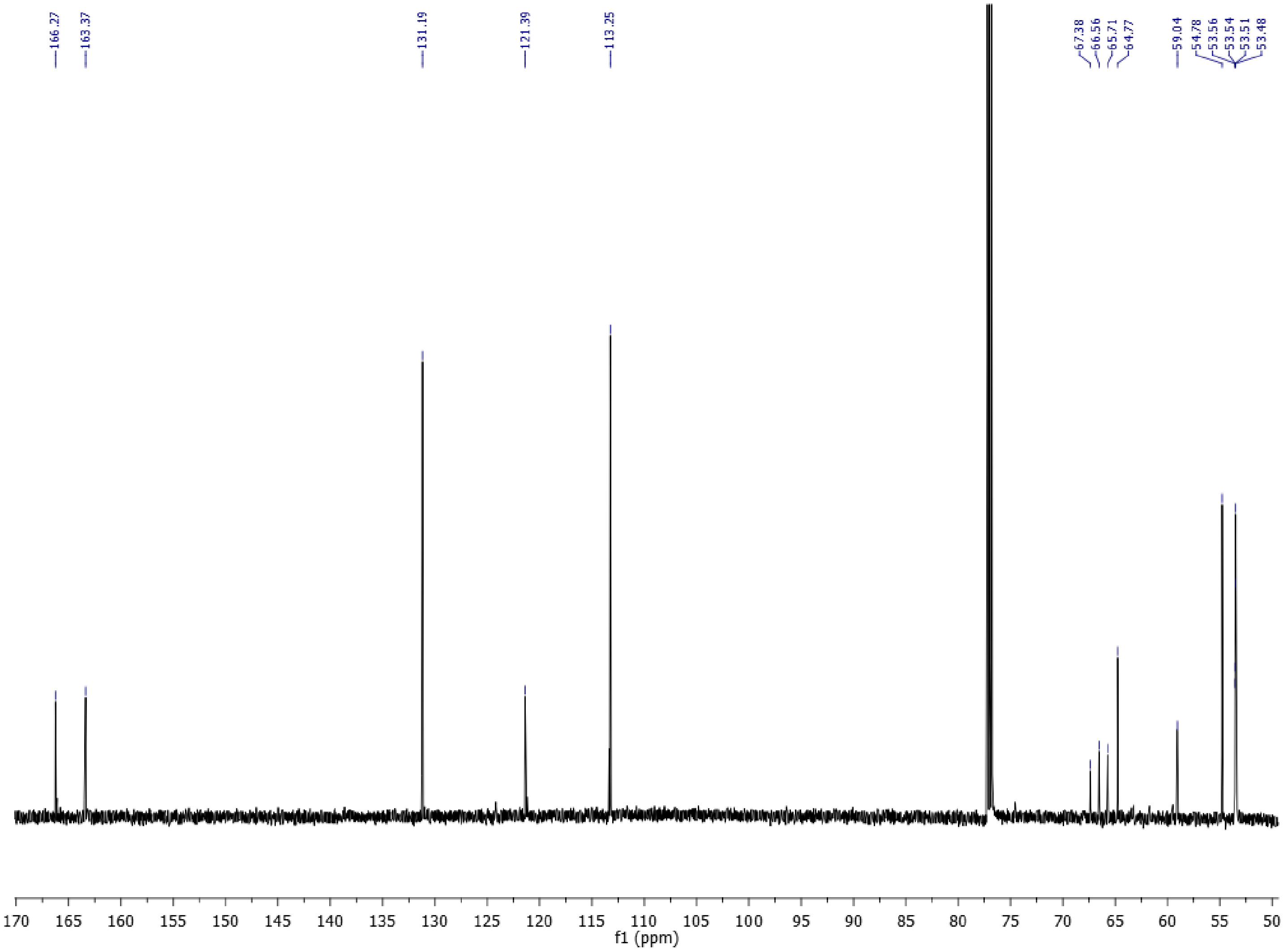

Figure S23: ^{31}P NMR spectrum of 7a.

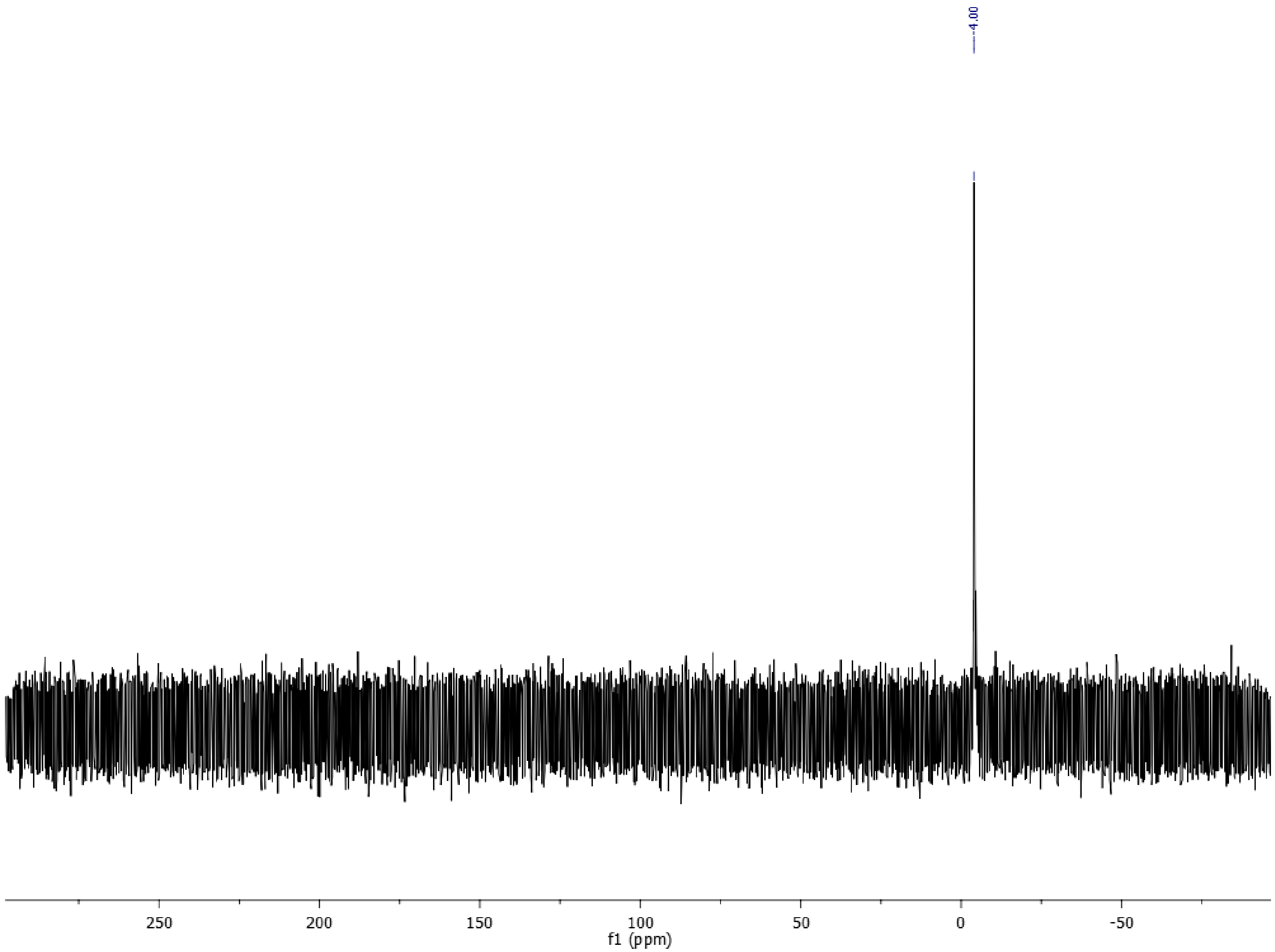

Figure S24: ^{1}H – ^{1}H COSY spectrum of 7a.

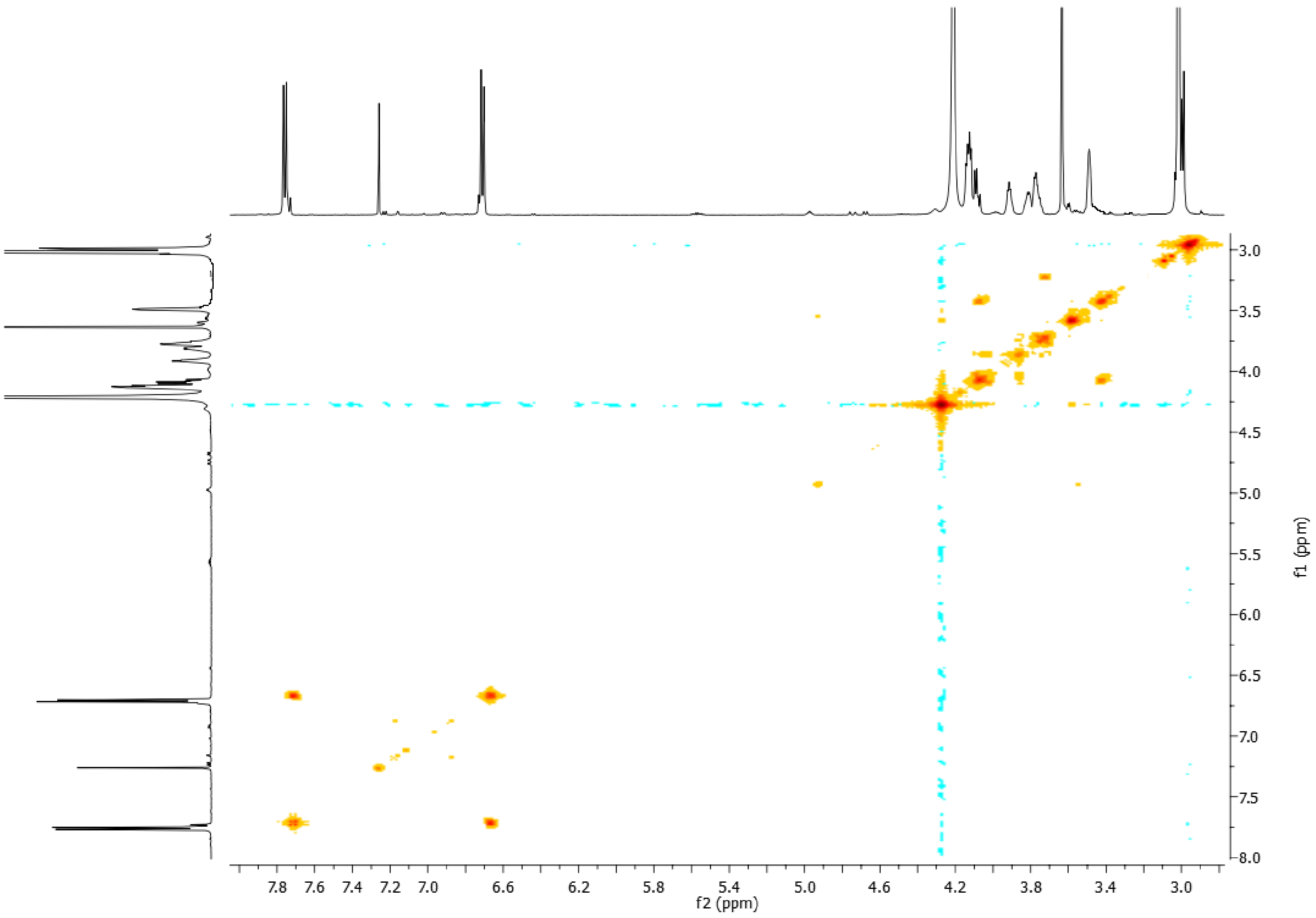

Figure S25: HSQC spectrum of 7a.

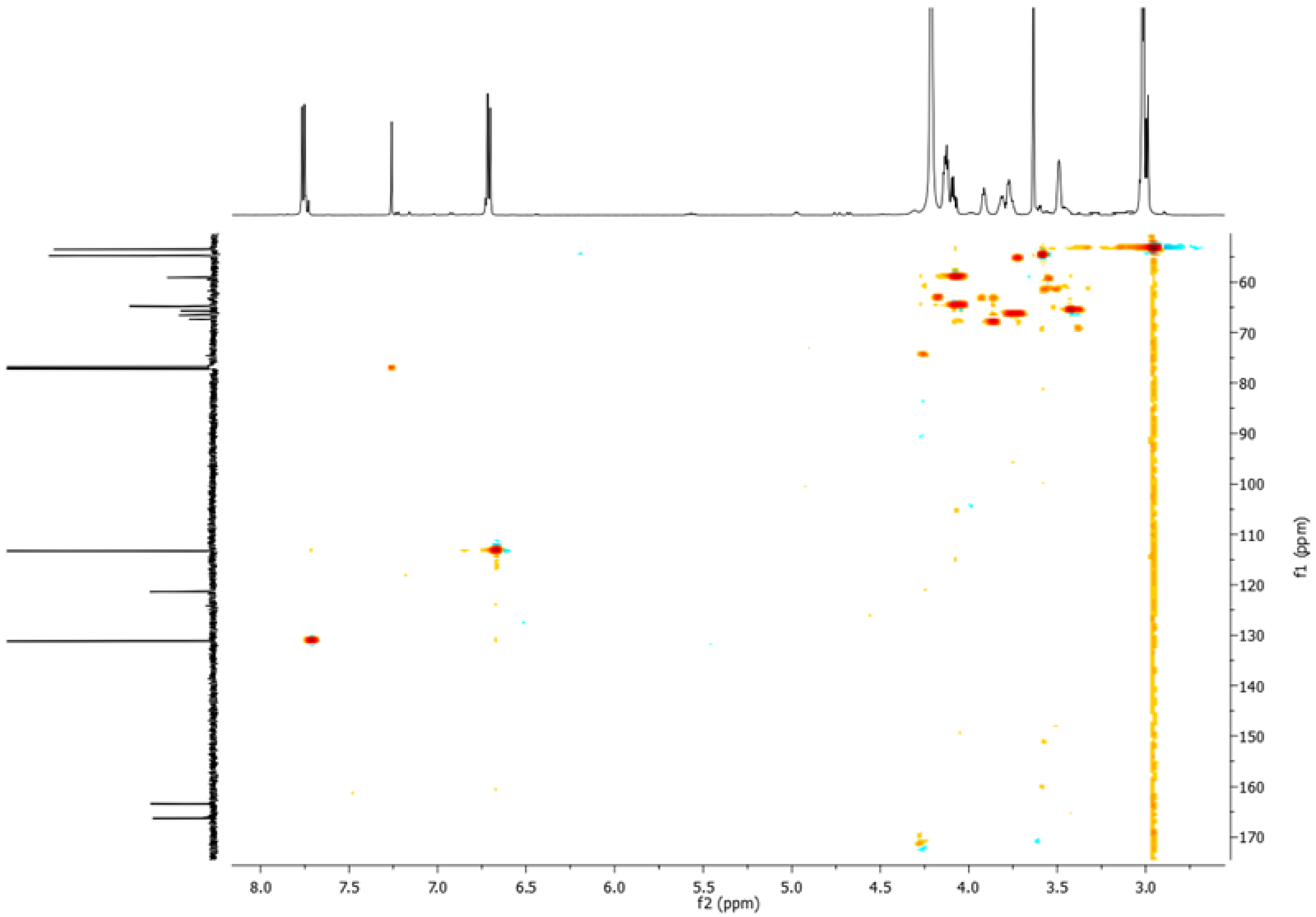

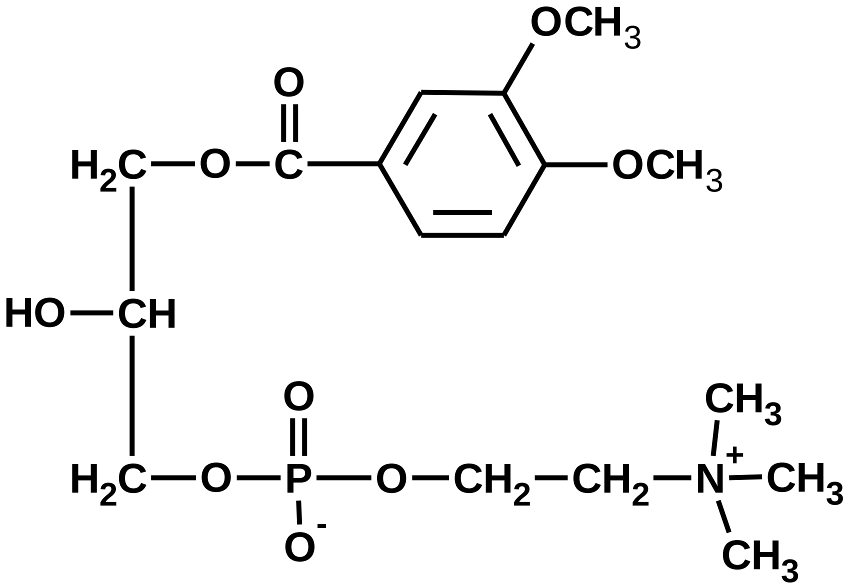

1-veratroyl-2-hydroxy-*sn*-glycero-3-phosphocholine (**7b**)

Figure S26: ^{1}H NMR spectrum of 7b.

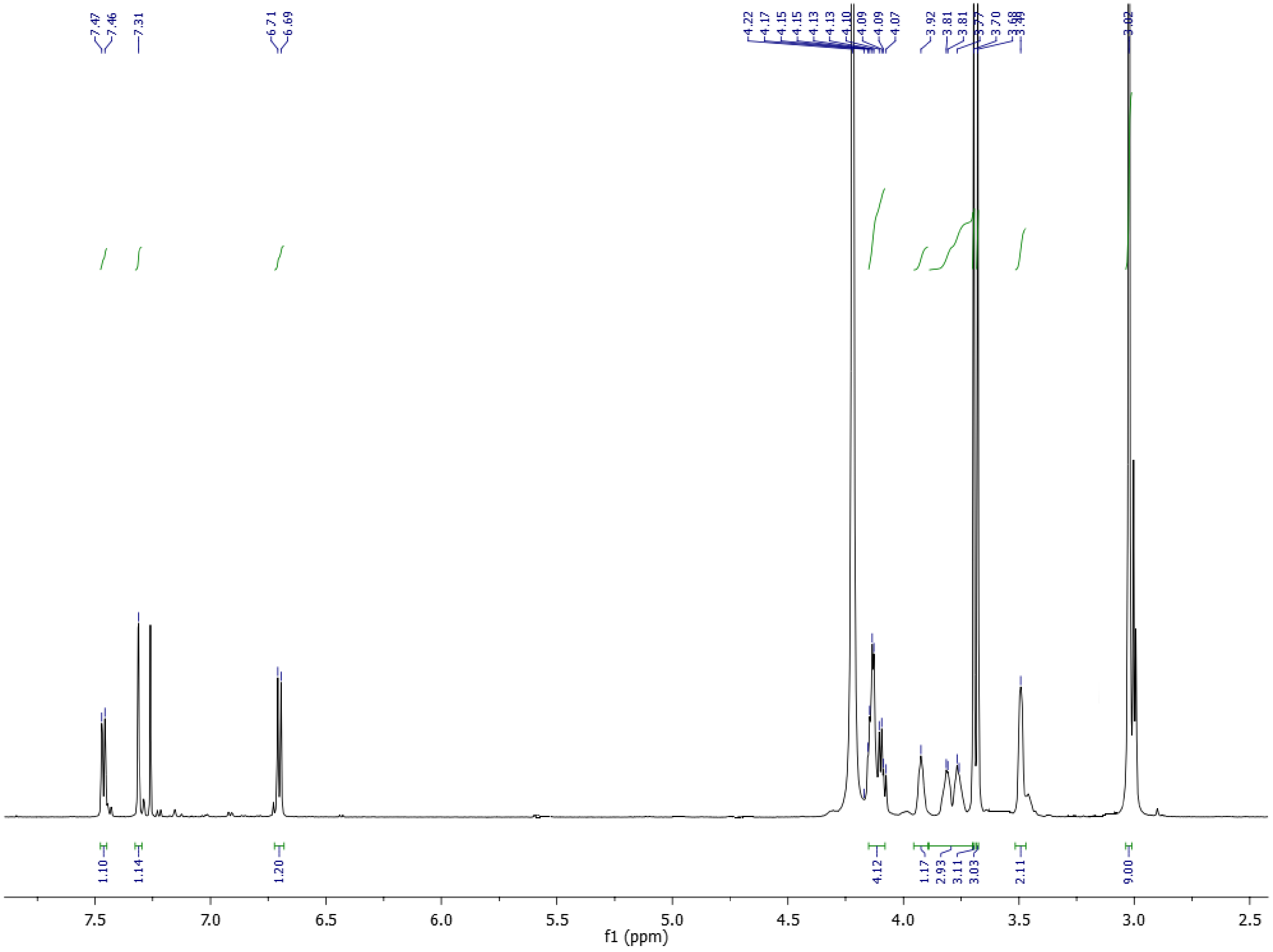

Figure S27: ^{13}C NMR spectrum of 7b.

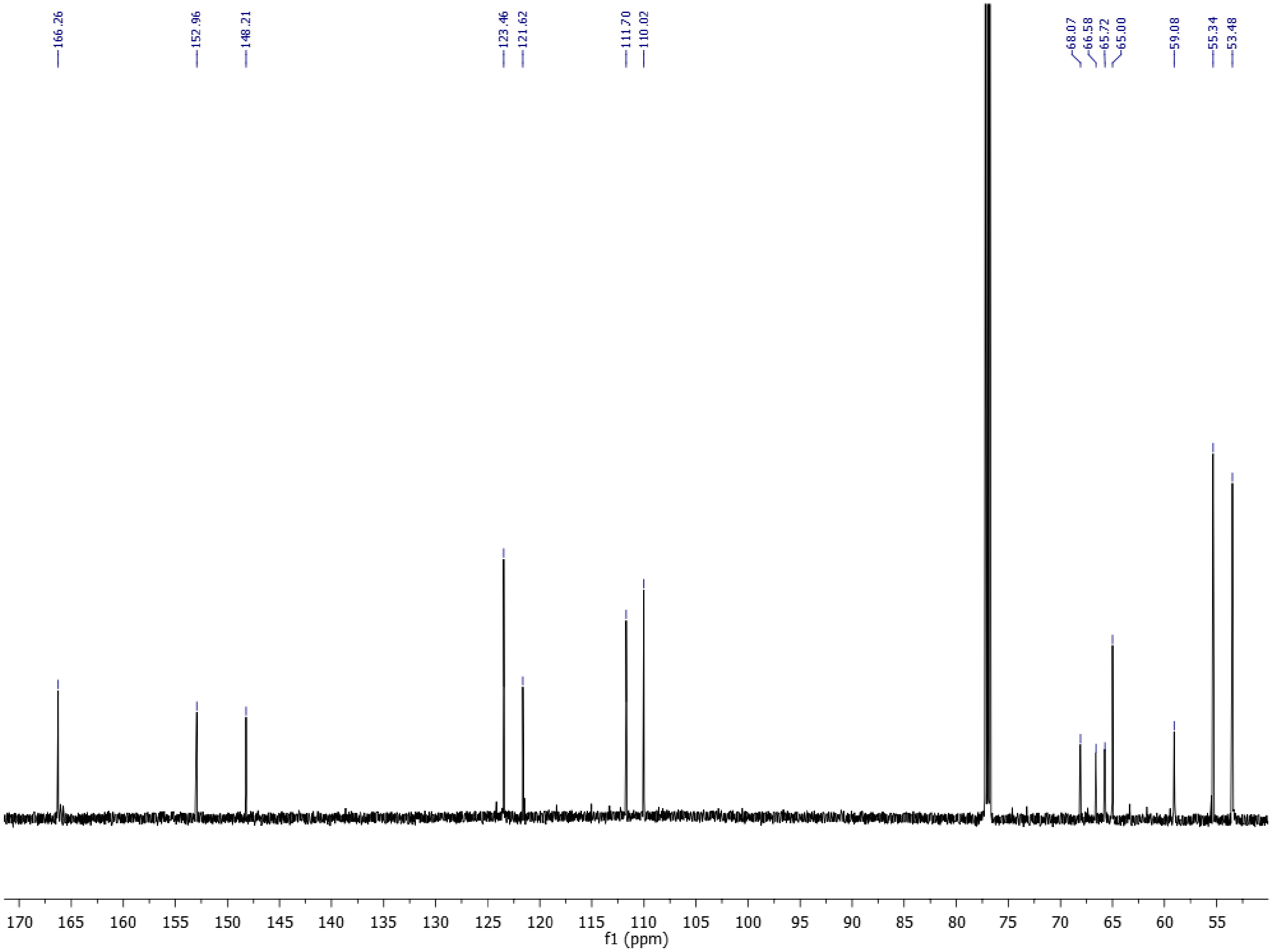

Figure S28: ^{31}P NMR spectrum of 7b.

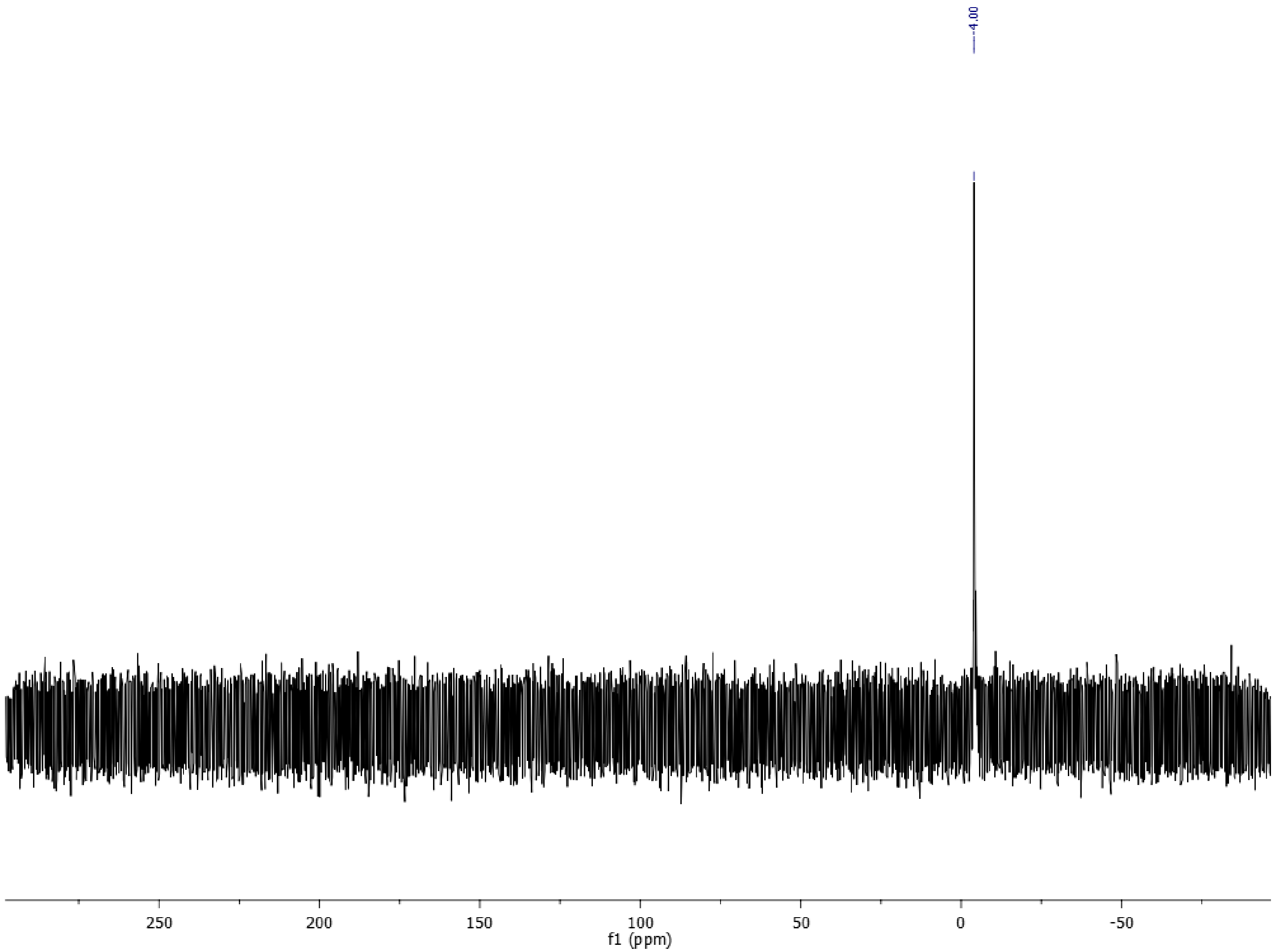

Figure S29: ^1H – ^1H COSY spectrum of 7b.

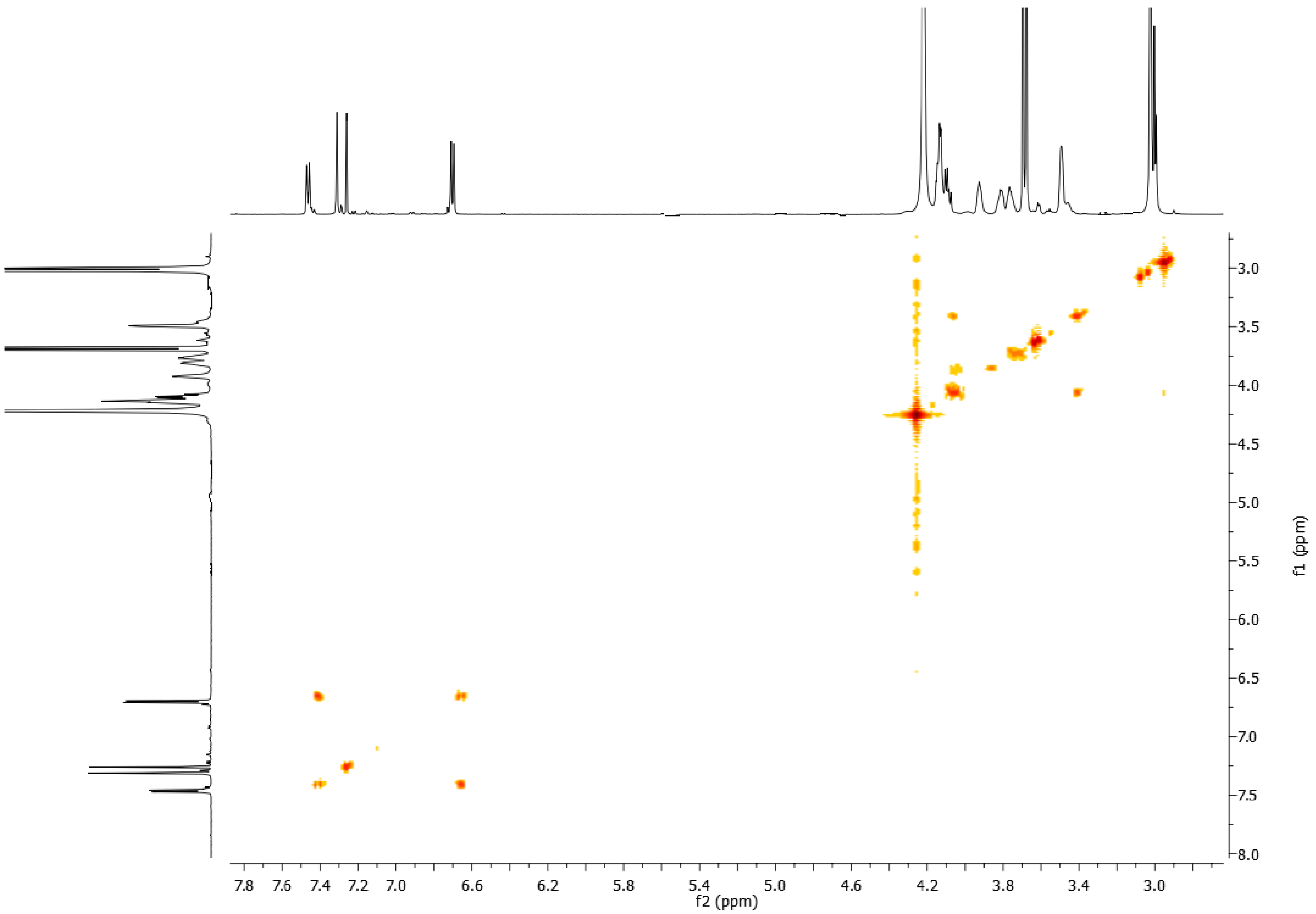

Figure S30: HSQC spectrum of 7b.

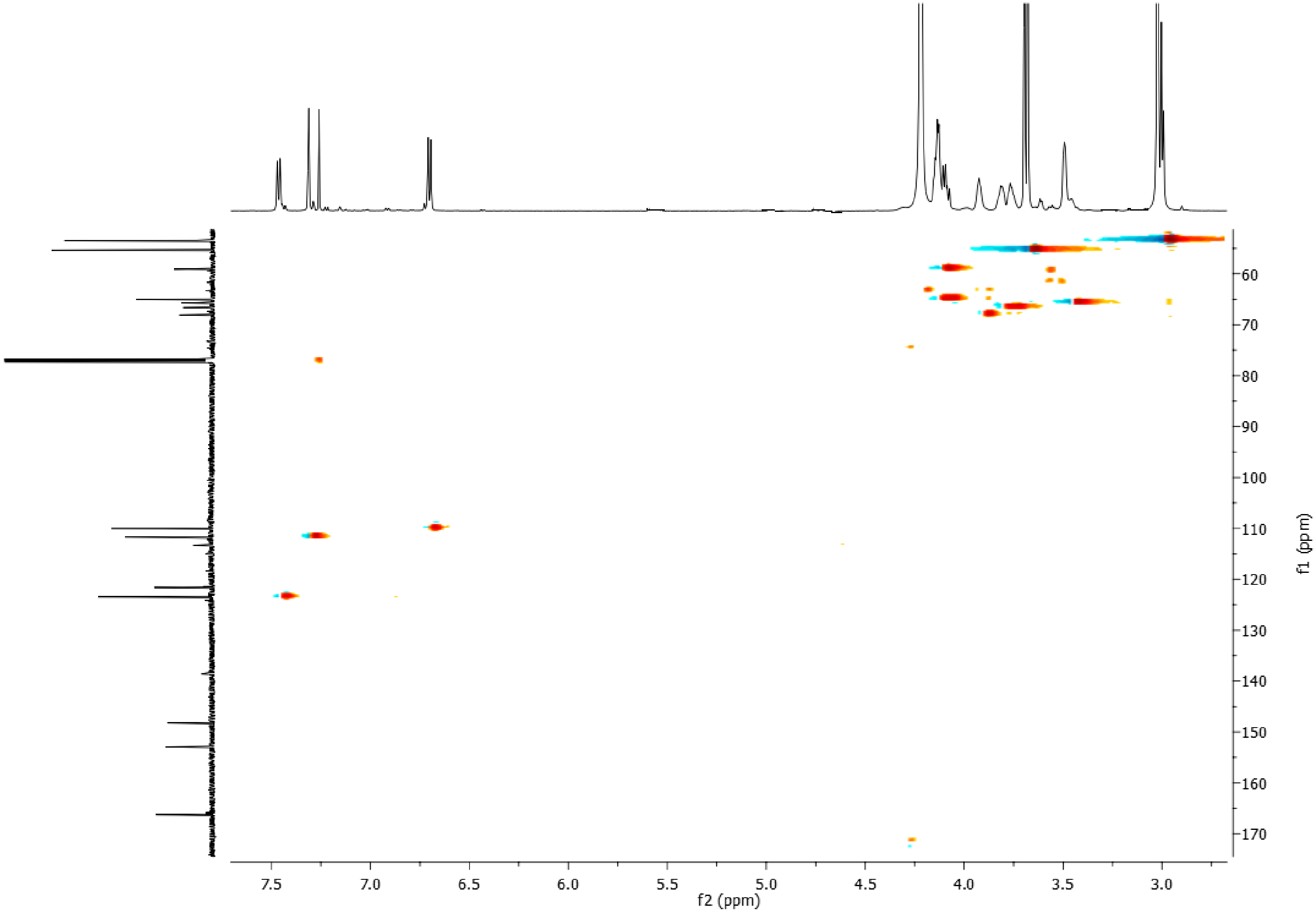

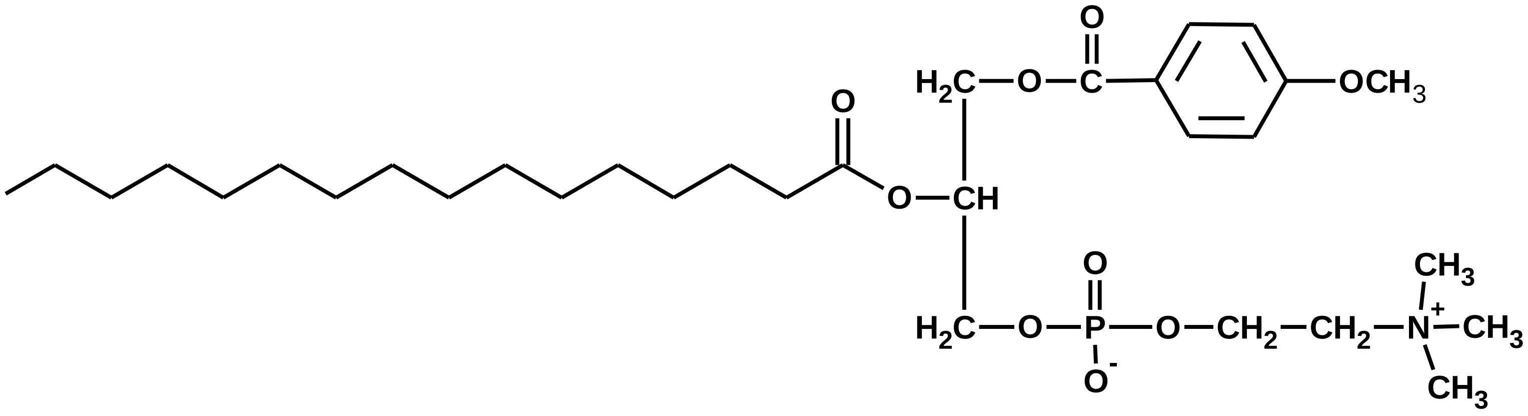

1-anisoyl-2-palmitoyl-*sn*-glycero-3-phosphocholine (**8a**)

Figure S31: ^{1}H NMR spectrum of 8a.

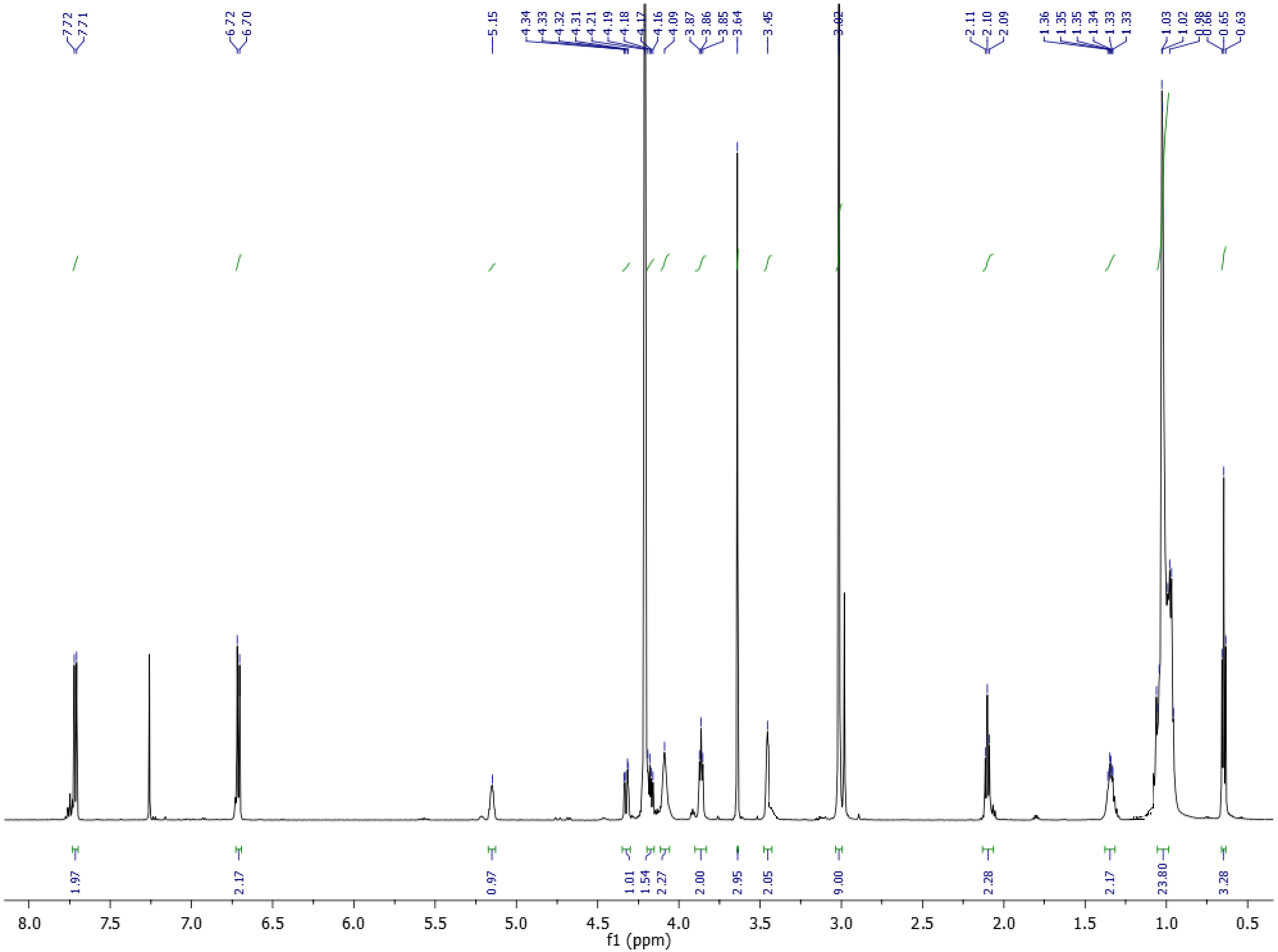

Figure S32: ^{13}C NMR spectrum of 8a.

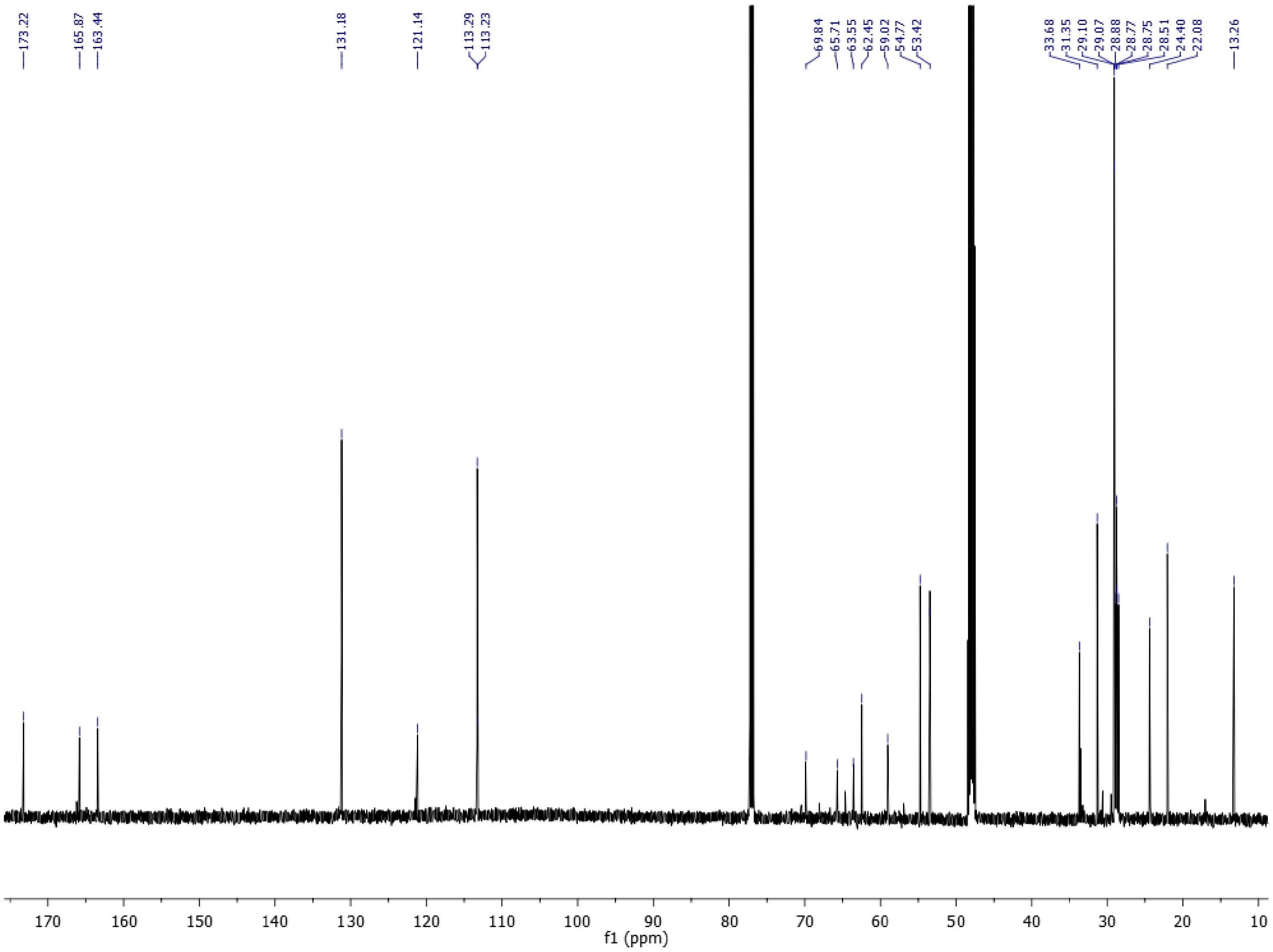

Figure S33: ^{31}P NMR spectrum of 8a.

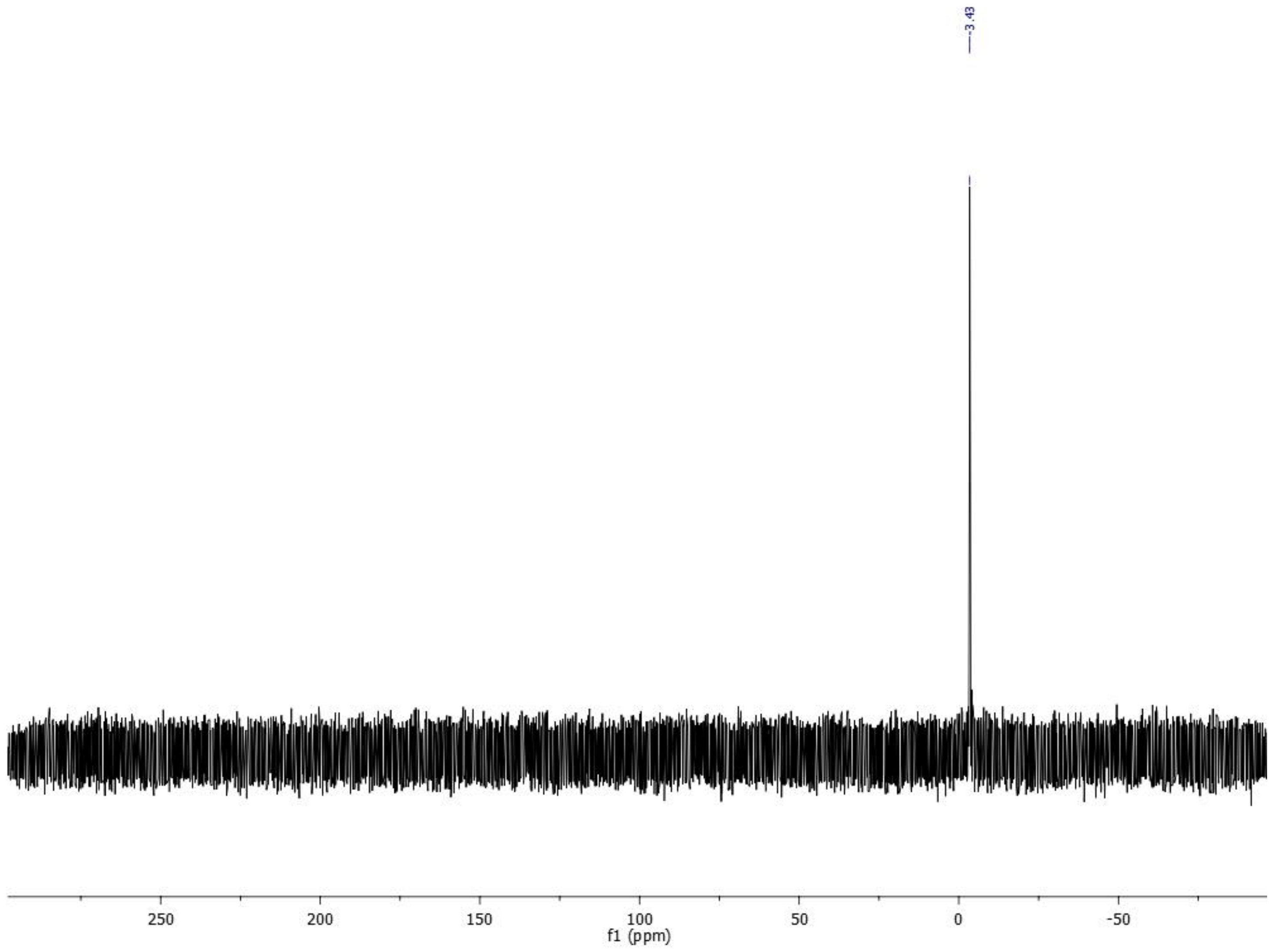

Figure S34: ^{1}H – ^{1}H COSY spectrum of 8a.

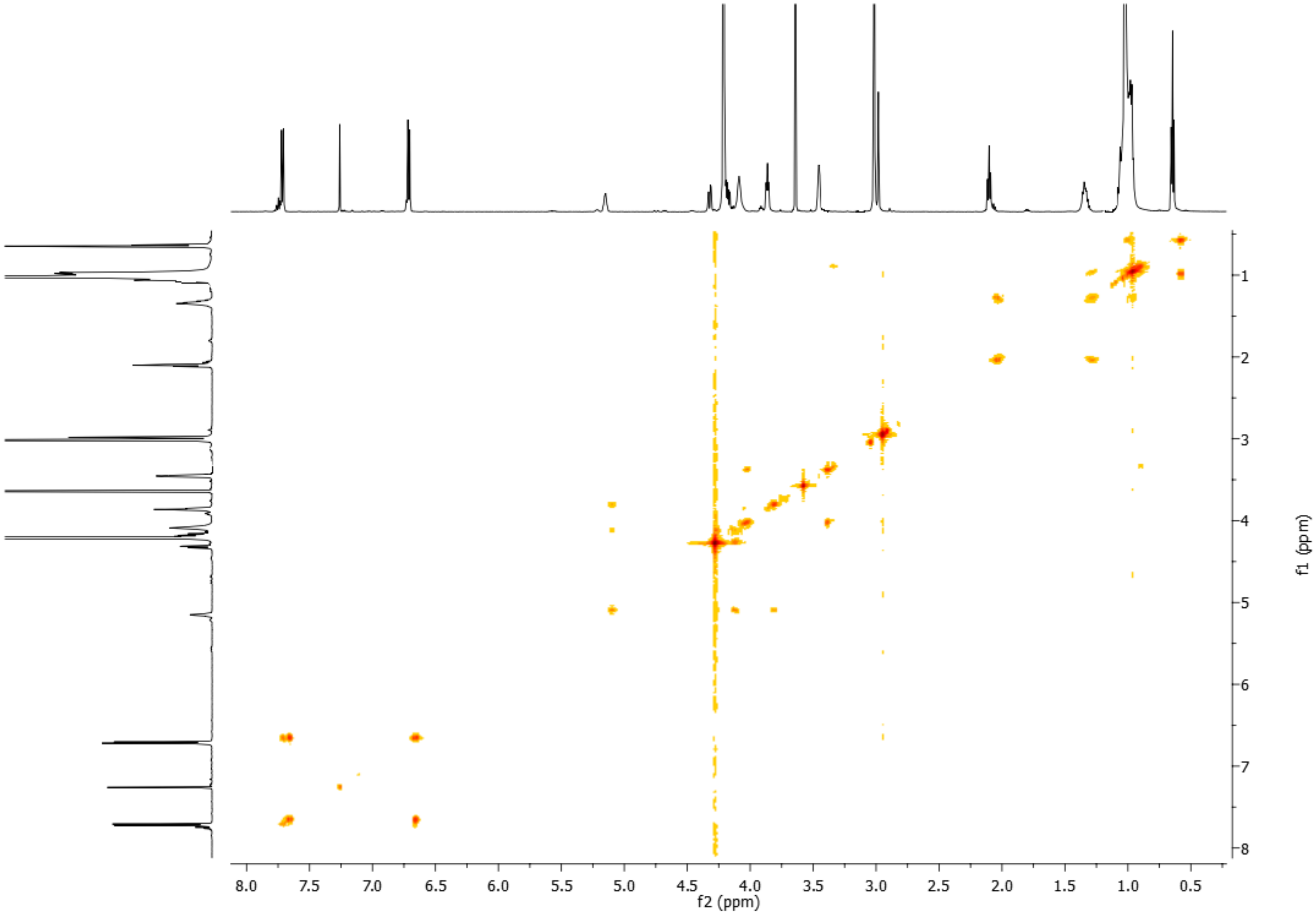

Figure S35: HSQC spectrum of 8a.

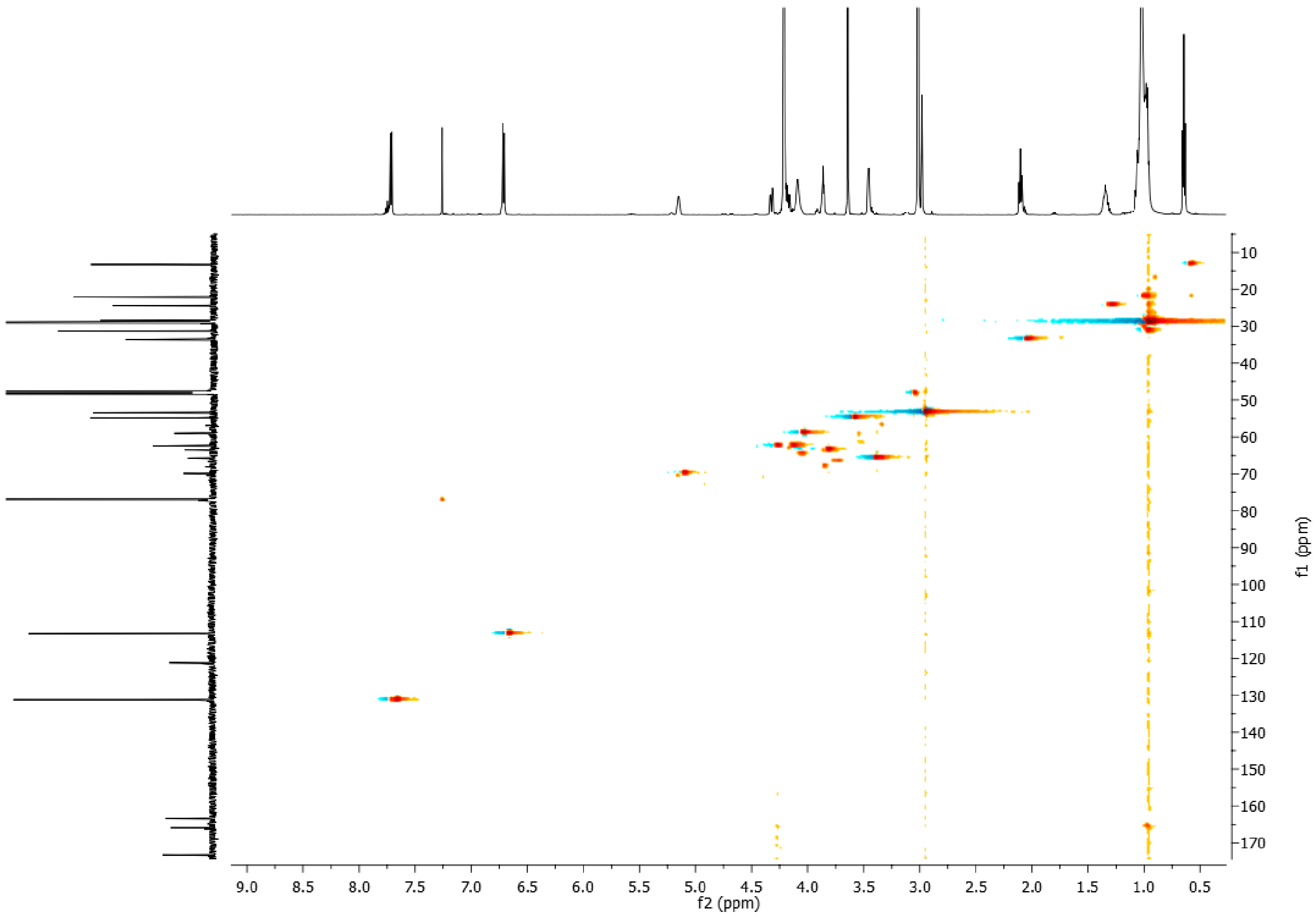

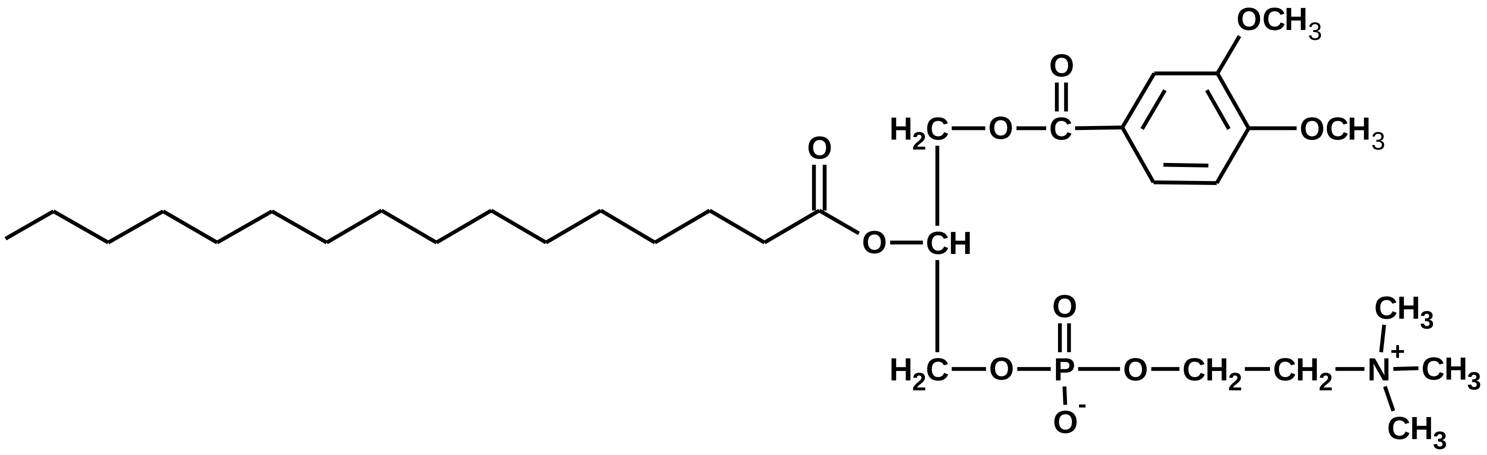

1-veratroyl-2-palmitoyl-*sn*-glycero-3-phosphocholine (**8b**)

Figure S36: ^{1}H NMR spectrum of 8b.

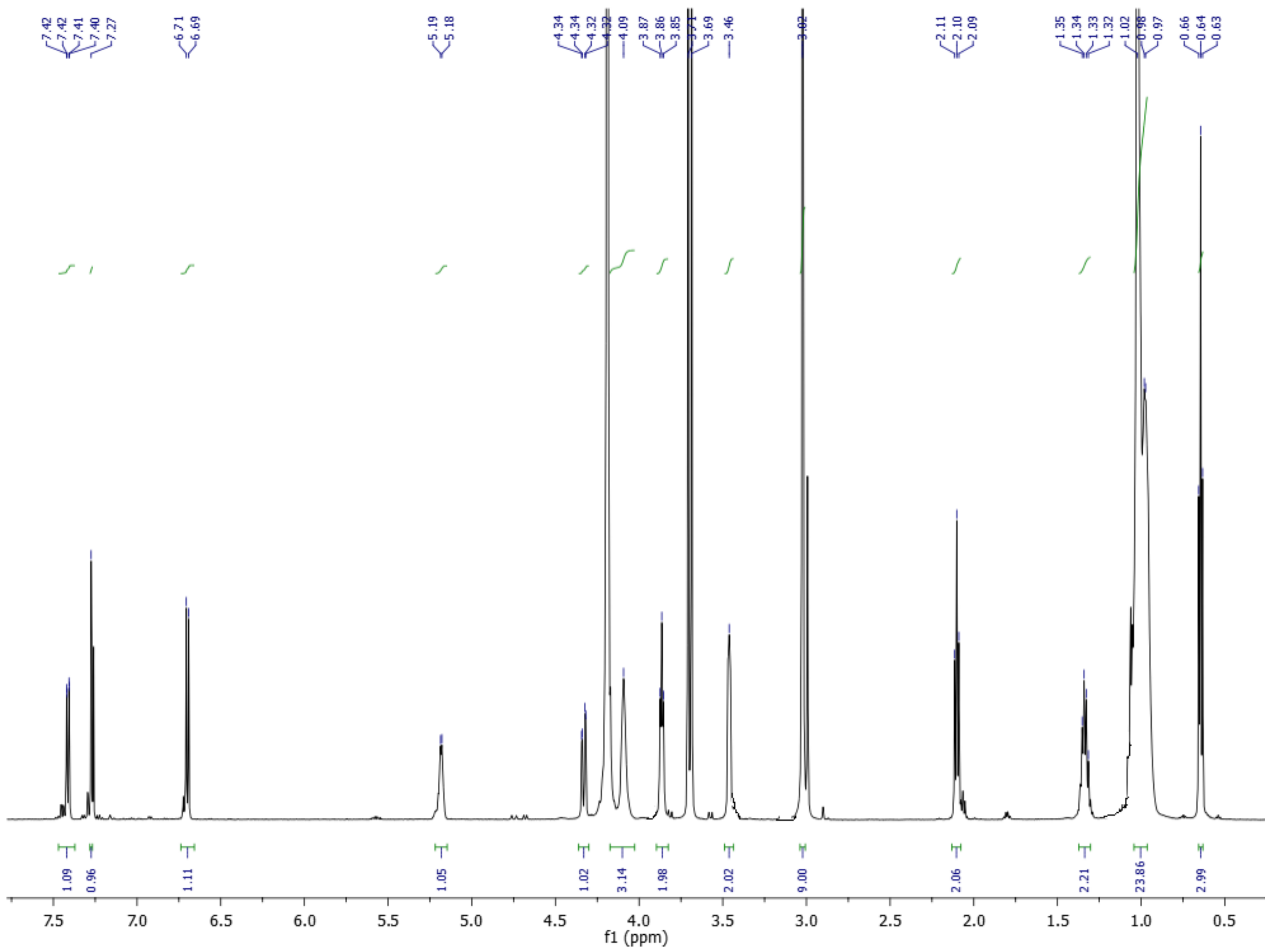

Figure S37: ^{13}C NMR spectrum of 8b.

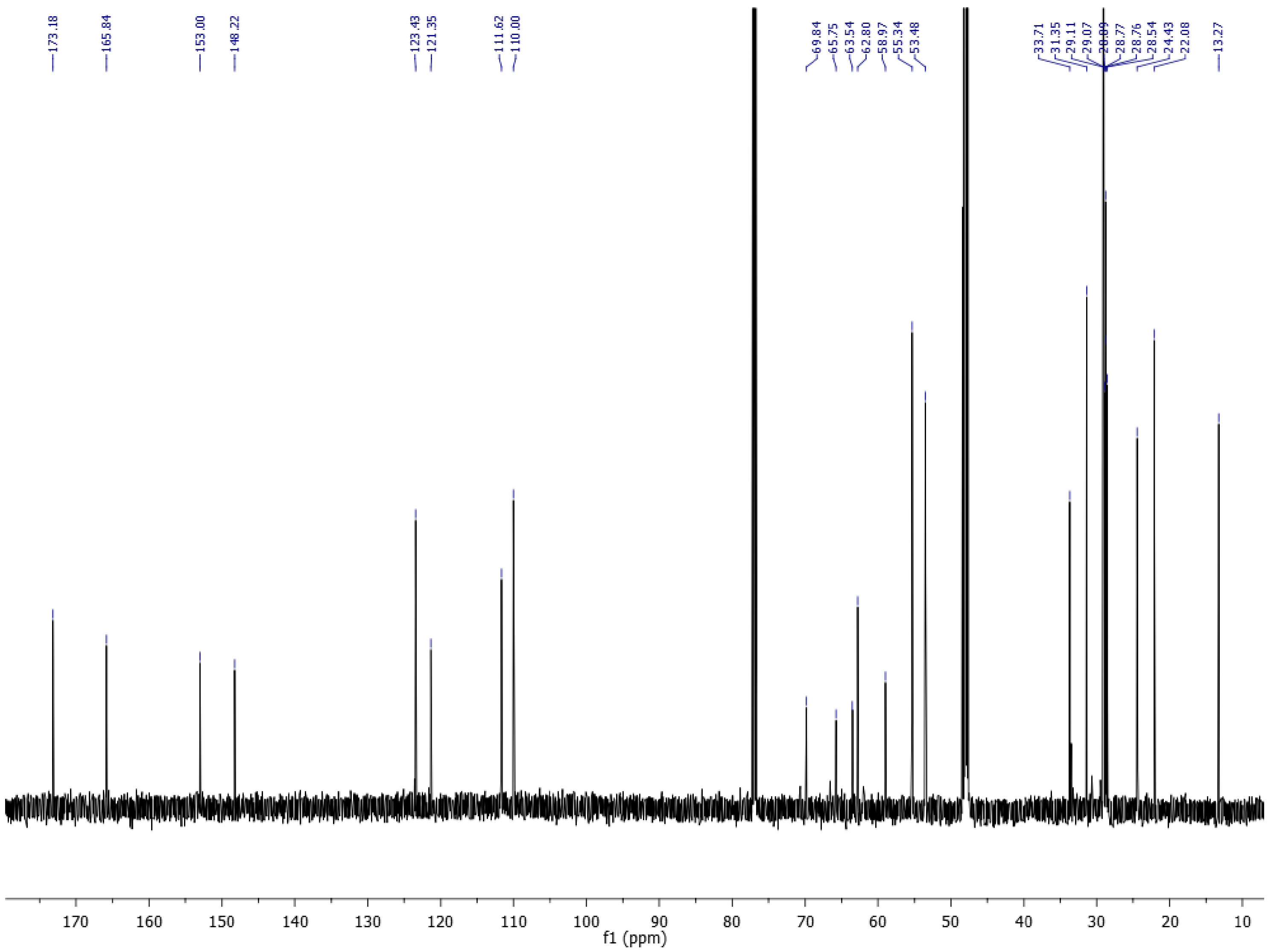

Figure S38: ^{31}P NMR spectrum of 8b.

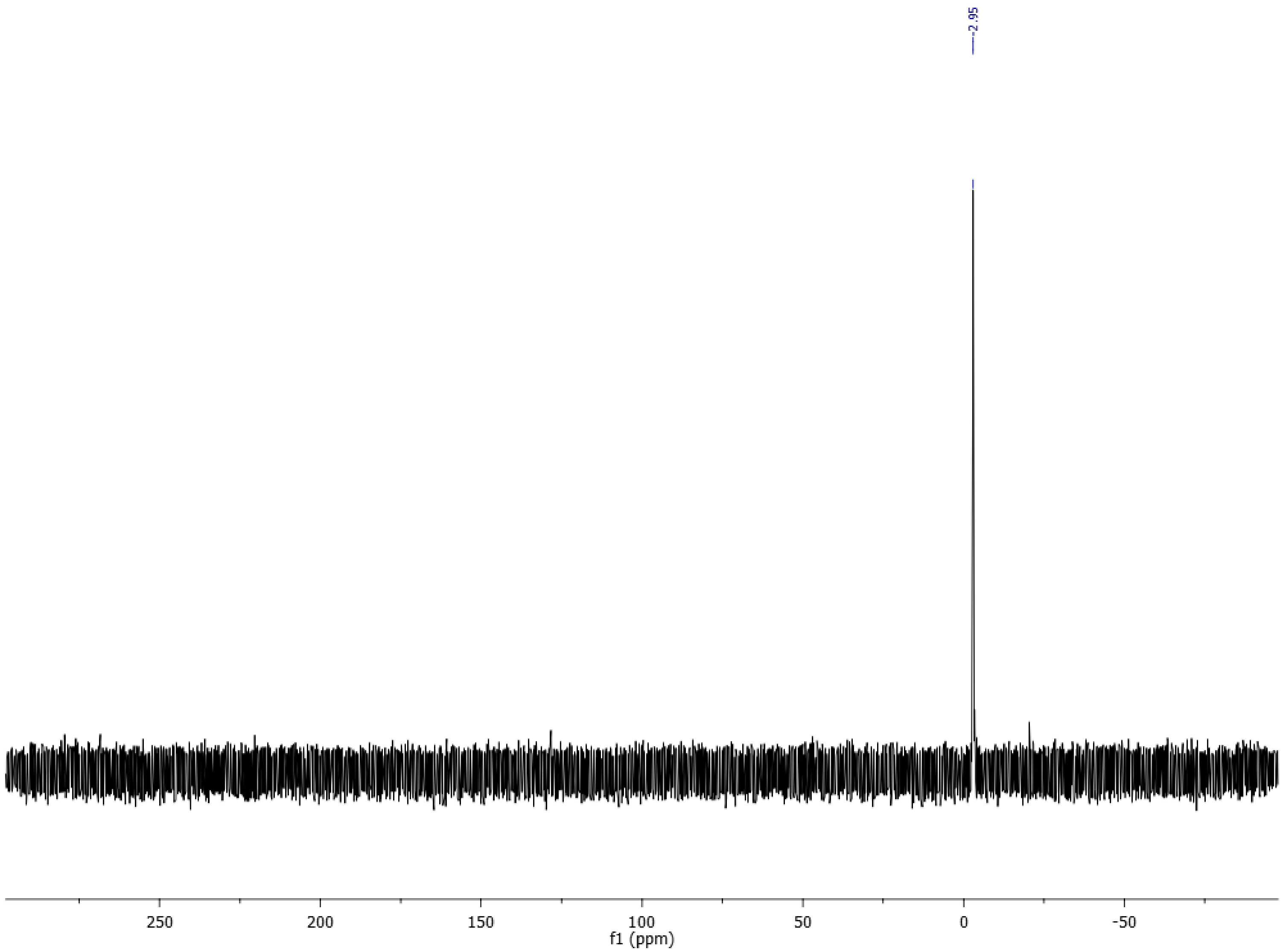

Figure S39: ^{1}H – ^{1}H COSY spectrum of 8b.

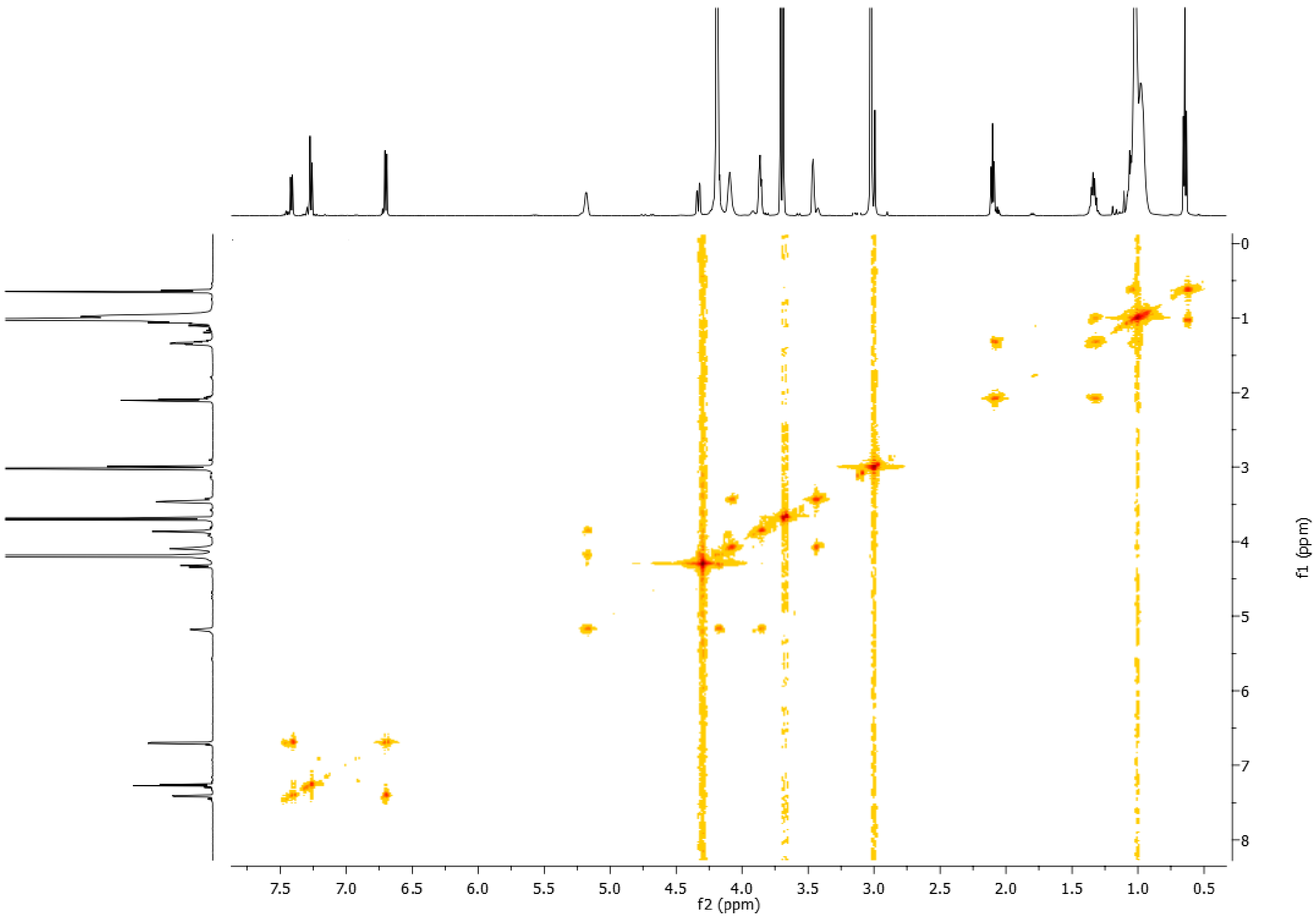

Figure S40: HSQC spectrum of 8b.

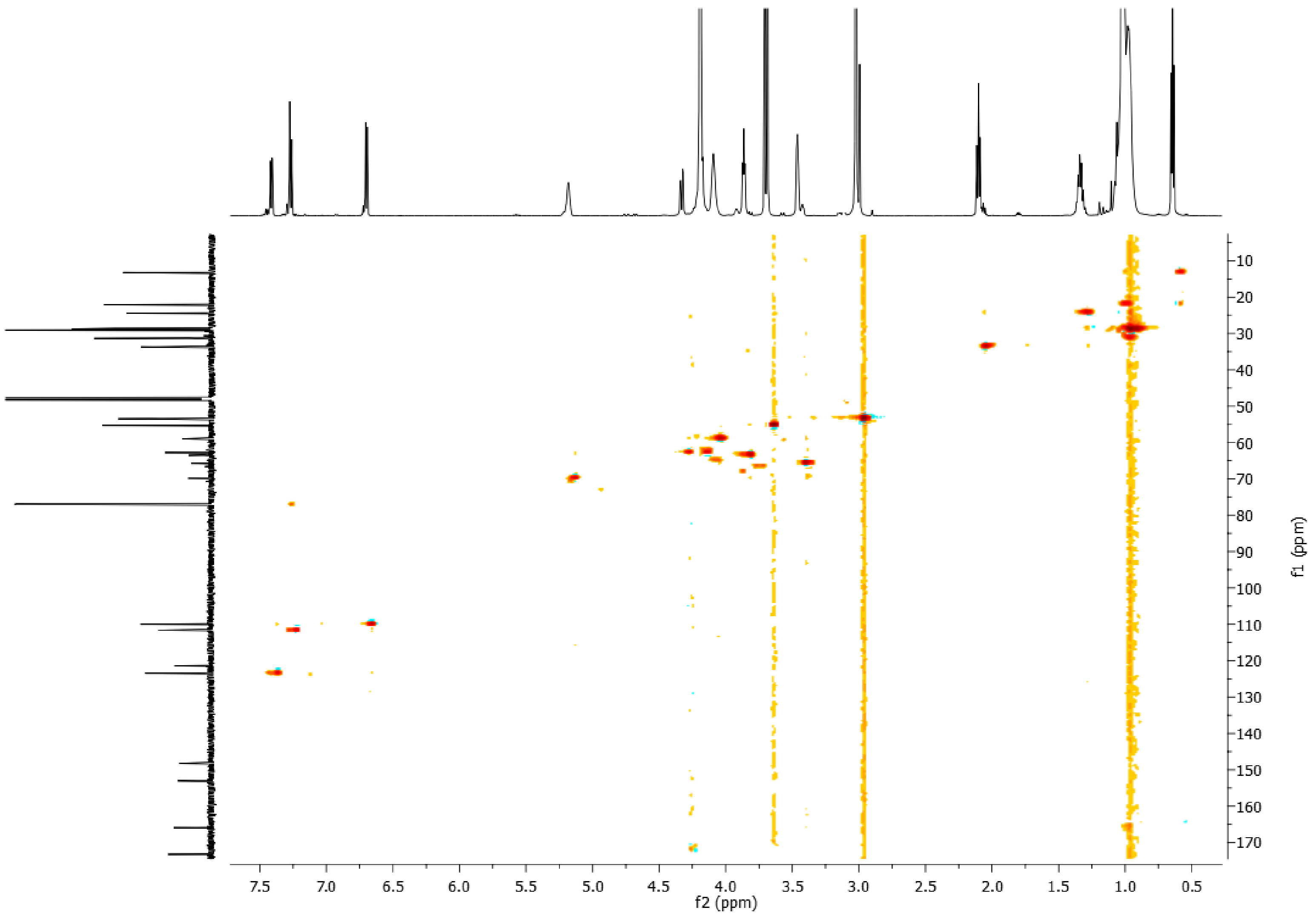

Supplement: Supplementary file 1 [file molecules-23-02022-s001.pdf]
